# Introducing Depth Information Into Generative Target Tracking

**DOI:** 10.3389/fnbot.2021.718681

**Published:** 2021-09-01

**Authors:** Dongyue Sun, Xian Wang, Yonghong Lin, Tianlong Yang, Shixu Wu

**Affiliations:** ^1^School of Mechanical Engineering, Hunan University of Science and Technology, Xiangtan, China; ^2^Changsha Shi-Lang Technology Co., Ltd., Changsha, China

**Keywords:** target tracking, confusion from similar background, introduction of depth information, data source, density distribution of dataset

## Abstract

Common visual features used in target tracking, including colour and grayscale, are prone to failure in a confusingly similar-looking background. As the technology of three-dimensional visual information acquisition has gradually gained ground in recent years, the conditions for the wide use of depth information in target tracking has been made available. This study focuses on discussing the possible ways to introduce depth information into the generative target tracking methods based on a kernel density estimation as well as the performance of different methods of introduction, thereby providing a reference for the use of depth information in actual target tracking systems. First, an analysis of the mean-shift technical framework, a typical algorithm used for generative target tracking, is described, and four methods of introducing the depth information are proposed, i.e., the thresholding of the data source, thresholding of the density distribution of the dataset applied, weighting of the data source, and weighting of the density distribution of the dataset. Details of an experimental study conducted to evaluate the validity, characteristics, and advantages of each method are then described. The experimental results showed that the four methods can improve the validity of the basic method to a certain extent and meet the requirements of real-time target tracking in a confusingly similar background. The method of weighting the density distribution of the dataset, into which depth information is introduced, is the prime choice in engineering practise because it delivers an excellent comprehensive performance and the highest level of accuracy, whereas methods such as the thresholding of both the data sources and the density distribution of the dataset are less time-consuming. The performance in comparison with that of a state-of-the-art tracker further verifies the practicality of the proposed approach. Finally, the research results also provide a reference for improvements in other target tracking methods in which depth information can be introduced.

## Introduction

Video target tracking refers to the continuous tracking of the state of a target in a sequence of frames subsequent to the given initial position and scale information of the target. This is the basis for high-level vision tasks, such as a visual measurement, visual navigation, and visual serving (Karakostas et al., 2020; Nousi et al., 2020a,b; Shen et al., 2020; Wang et al., 2020). In practical applications, similar-looking backgrounds and target-scale changes may lead to a failure of the target tracking (Kashiani and Shokouhi, 2019; Makhura and Woods, 2019; Zhou et al., 2020), which remains a challenging task.

The existing target tracking methods are roughly divided into two categories: discriminant and generative methods (Chen et al., 2020). Discriminant methods that address tracking by discriminating the foreground from the background of an image are closely related to target detection (Li and Zheng, 2020), image segmentation (Bhandari et al., 2020; Guan et al., 2021), and other technologies. The boosting method (Yang et al., 2016), support vector machine (Feng et al., 2018), and deep neural network (Tong et al., 2019; Jain et al., 2020) are all good approaches for discriminant target tracking. Generative tracking methods rely on certain tracking strategies to determine the optimal solution from many candidate targets. They can be divided into methods based on the kernel density estimation, methods based on subspace, and methods based on a sparse representation (Dash and Patra, 2019; Luo et al., 2019; Wang and Zhang, 2019; Yan et al., 2019; Chen et al., 2020). These methods improve the respective characteristics and scope of the applications. In general, the discriminant methods are more robust, whereas the generative methods are independent from the training samples and are easy to implement and apply in real-time.

Common visual features used in target tracking, including colour, grayscale, derivative histogram, optical flow, texture, and key points, are prone to failure in confusingly similar-looking backgrounds. This problem will not be eradicated if no additional information source for the target tracking is introduced (Hu et al., 2017; Kang et al., 2018; Guan et al., 2020). In the field of computer vision, depth information refers to the position information of the target in the direction perpendicular to the image plane, which cannot be obtained by traditional monocular cameras. In recent years, three-dimensional visual information acquisition technologies such as time-of-flight (TOF) cameras, RGB-D cameras, and light detection and ranging (LiDAR) have gradually become popular. This means that, in addition to traditional appearance information, the conditions allowing the depth information to be widely used in target tracking have become available. This has drawn the attention of scholars focusing on how to introduce depth information into a target tracking algorithm and improve its performance. For example, Wang Y. et al. (2020) proposed a robust fusion-based RGB-D tracking method that integrates depth data into a visual object tracker to achieve the robust tracking of a target. In addition, Xiao et al. (2019) proposed a new tracking method that uses a kernel support vector machine (SVM) online learning classifier to detect and track a specific target in a single RGB-D sensor. Cao et al. (2015) also proposed a method that uses depth information obtained from a moving binocular camera to detect and recover occlusions. Experimental results demonstrate the robustness of the proposed 3D tracking method based on a comparison with several state-of-the-art tracking methods. Wu et al. (2015) proposed a robust fused tracker that utilises the depth information to achieve accurate 3D hand tracking even under extremely complex scenes. Existing research on video target tracking into which depth information is introduced has mostly focused on discriminant methods and seldom on generative approaches. It is pertinent to further expand and deepen research in this area.

A mean-shift is a typical generative method for tracking targets based on a kernel density estimation. It follows a certain similarity measure criterion to calculate the degree to which every region of a data source image matches the visual features of the tracked target. The calculation results are presented in the form of the density distribution of the two-dimensional dataset, and then starts from the initial position to locate the target by finding the local extrema. Although new methods of target tracking have emerged one after the other, the mean-shift algorithm, owing to its strong practicability and good comprehensive performance, is still widely used in actual systems, and many studies have been devoted to further improving its performance. For example, Hu et al. (2018) proposed an anti-occlusion video target tracking based on a prediction-algorithm-based strategy and re-matching to address the problem where the mean-shift algorithm is likely to fail in the case of a target occlusion. The results showed that the method takes credit for the strong capability of anti-occlusion and reliability tracking during the video target tracking process. In addition, Iswanto et al. (2019) combined the mean-shift algorithm with both particle and Kalman filtering and obtained a good overall performance. Ap and Na (2020) reduced the computational complexity of the mean-shift algorithm by applying speeded-up robust features (SURF) and re-projection techniques. Moreover, Sliti et al. (2017) proposed four adaptive scale and orientation mean shift trackers, which proved to be more stable and less prone to drifting away from the target than purely coloured or feature-based trackers.

To improve the performance of the generative target tracking methods widely used in actual systems, relevant studies conducted both at home and abroad based on the mean-shift approach, which is a typical technology framework of generative target tracking, were referenced. In this article, four target tracking methods based on the similarity measure criteria of the histogram back projection into which the depth information is introduced are proposed, i.e., a thresholding of the data source, thresholding of the density distribution of the dataset, weighting of the data source, and weighting of the density distribution of the dataset. Experimental research has verified at length the validity of the improvement methods and arrived at a discussion regarding their characteristics. To the best of our knowledge, a more comprehensive discussion of the possible ways to introduce depth information into generative target tracking methods based on a kernel density estimation as well as on the performance of different introduction methods has yet to be conducted.

## Mean-Shift Target Tracking Method

To apply the mean-shift algorithm for target tracking, one must first calculate the degree to which every area of an image matches the visual features of the target being tracked using a certain method, and then present the calculation result in the form of the density distribution of the two-dimensional dataset. A histogram back projection is a common method for constructing the density distribution of a two-dimensional dataset. To do so, one should rely on certain digital features (e.g., grey or in colour) of digital image pixels as the source of the data structure for the histogram, assuming that there are a total number of *t* pixels within the tracked target, and that the coordinates of each pixel are expressed as (*x*_1_, *y*_1_), (*x*_2_, *y*_2_), …, (*x*_*t*_, *y*_*t*_). The target histogram with a quantisation level of *m* is *h* = {*h*_*u*_}, where *u* = 1, 2, ⋯ , *m* and

(1) $$h_{u}=\sum_{i=1}^{t}\delta \left[G\left(x_{i},y_{i}\right)-f_{u}\right].$$

In the equation, δ[•] is the Kronecker delta function, and *G*(*x*_*i*_, *y*_*i*_) is the digital eigenvalue of the pixel with coordinates (*x*_*i*_, *y*_*i*_). In addition, *f*_*u*_ is the value range covered by the digital feature at level *u*, in which the digital feature *G*(*x, y*) at (*x, y*) in frame *G* of the tracking image is within the interval *f*_*s*_. Image *P* of the histogram back projection is obtained by setting the grayscale at (*x, y*) to *P*(*x, y*):

(2) $$P(x,y)=\frac{h_{s}}{\max\left\{h_{1,}h_{2},\cdots ,h_{m}\right\}}\times Q,$$

where *Q* is the highest grayscale value of the back projection *P* (given that the back projection is usually in an 8-bit unsigned integer data structure, and the value of *Q* is 255).

With the two-dimensional dataset density distribution derived from the histogram back projection, the mean-shift tracking algorithm makes it possible to track the target by iteratively locating the region most similar to the target:

Step 1 - Based on the position in the last frame, place the initial search window somewhere in the density distribution of the two-dimensional dataset.
Step 2 - Calculate the centroid coordinates (*x*_*c*_, *y*_*c*_) of the search window:
  (3) $$x_{c}=\frac{M_{10}}{M_{00}},$$
  (4) $$y_{c}=\frac{M_{01}}{M_{00}},$$
  where *M*_00_ is the zeroth moment of the search window; *M*_10_, and *M*_01_ are the first moment thereof:
  (5) $$M_{00}=\sum_{x_{j}}\sum_{y_{j}}P(x_{j},y_{j}),$$
  (6) $$M_{10}=\sum_{x_{j}}\sum_{y_{j}}x_{j}\times P(x_{j},y_{j}),$$
  (7) $$M_{01}=\sum_{x_{j}}\sum_{y_{j}}y_{j}\times P(x_{j},y_{j}),$$
  where *x*_*j*_ and *y*_*j*_ are the coordinates of the spot within the search window.

Step 3 - Move the centre of the search window to the position of the centroid along the mean-shift vector.
Step 4 - If the displacement *s* of the centre of the search window is less than the threshold ε or the number of iterations *n* is greater than the threshold *N*, the current search window position is output as the target location and the iteration ends; otherwise, return to step 2.

**Figure 1:**
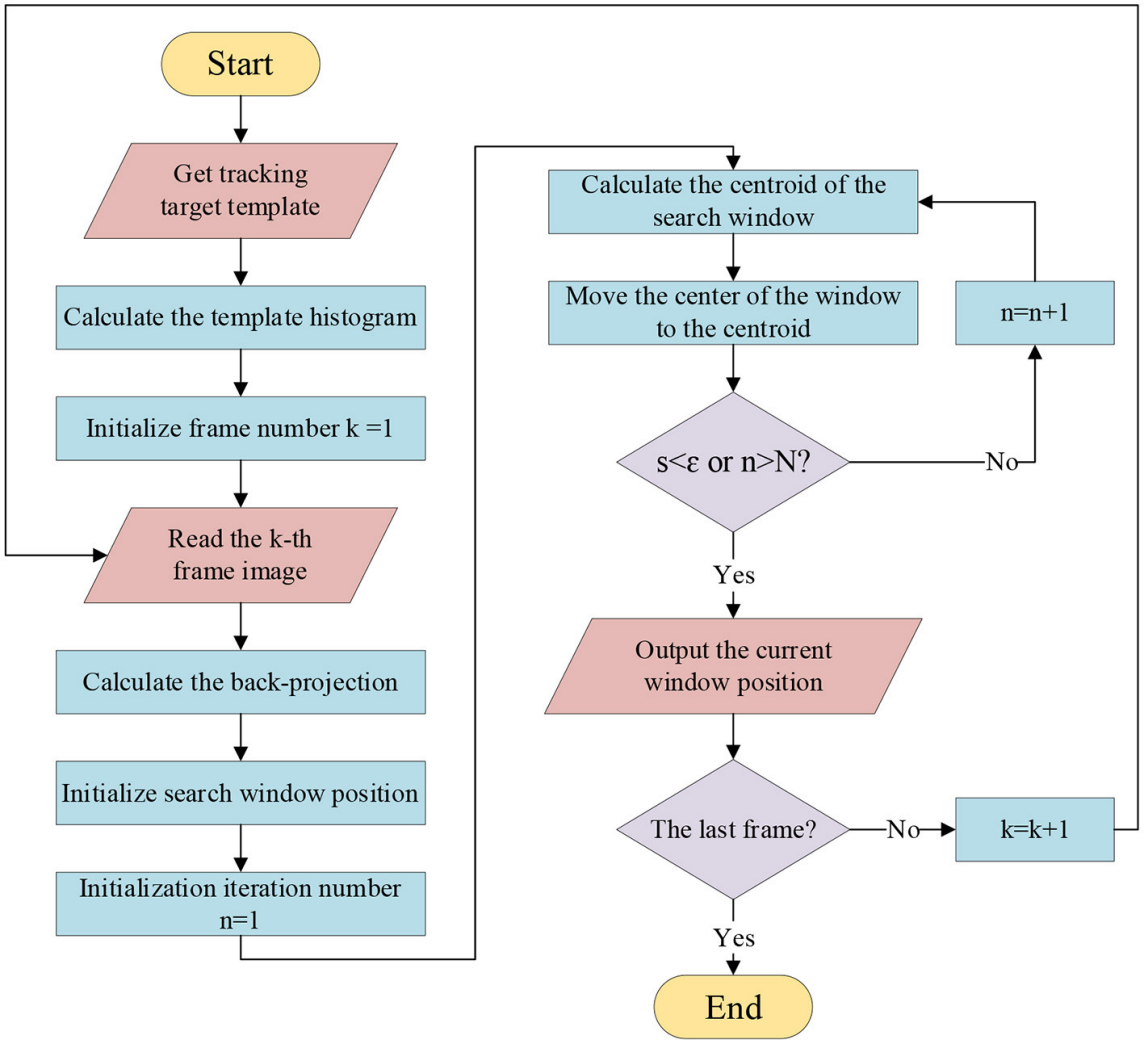
Algorithm flow of the mean-shift using back projection.

In summary, the algorithm flow by which the mean-shift method tracks a target in a sequence of frames is shown in Figure 1.

## Methods for Introducing Depth Information

Although the technology to acquire three-dimensional visual information has been proven, the introduction of depth information to improve traditional target tracking methods promises better robustness. In the mean-shift tracking framework, depth information can be introduced at the data source level or at the density distribution level of the dataset. In terms of the fusion method, one may set the range of the possible presence of the target based on depth information or use the depth information to weight the data. Based on the above, this article proposes four methods, into which depth information is introduced to improve the mean-shift algorithm.

### Introducing Depth Information Into the Thresholding of the Data Source

The basic idea of the algorithm is to exclude the area where the depth value is excessively different from the depth of the target at the last moment from the candidate area where the target is located, thereby improving the performance of the target tracking algorithm. In the methods described in section Mean-Shift Target Tracking Method, depth information is introduced into the thresholding of *G* by the following formula once the grayscale image (the data source) to be tracked becomes available:

(8) $$TG\left(x,y\right)=\{\begin{array}{cc} G\left(x,y\right) & \;T_{1}<D\left(x,y\right)<T\\ 0 & \;other\text{s} \end{array},$$

where *D*(*x, y*) is the grayscale value of the point (*x, y*) in the depth image *D* corresponding to *G*; *T*_2_ and *T*_1_ are the high and low depth thresholds, respectively. In the algorithm, the grayscale image *TG*, thresholded after the introduction of the depth information, takes the place of the original grayscale image *G* used in the method described in section Mean-Shift Target Tracking Method, thereby serving as the data source for the calculation of the back projection.

Compared with the traditional mean-shift target tracking method, this algorithm adds no more than 2 × M × N comparison operations in a single tracking (when the detected image size is M pixels × N pixels).

### Introducing Depth Information Into the Thresholding of the Density Distribution of the Dataset

The basic idea of this algorithm is the same as that of the algorithm described in section Introducing Depth Information Into the Thresholding of the Data Source. In the method described in section Mean-Shift Target Tracking Method, depth information is introduced into the thresholding of *P* using the following formula once back projection *P* (the density distribution of the dataset) of the grayscale image to be tracked becomes available:

(9) $$TP\left(x,y\right)=\{\begin{array}{cc} P\left(x,y\right) & \;T_{1}<D\left(x,y\right)<T\\ 0 & \;others \end{array},$$

where *D*(*x, y*), *T*_2_, and *T*_1_ each have the same meaning as in (3). In this algorithm, the original back projection *P* is replaced by the back-projection TP threshold after the introduction of the depth information.

This algorithm needs the same additional computing resources as the method described in section Introducing Depth Information Into the Thresholding of the Data Source.

### Introducing Depth Information Into the Weighting of the Data Source

The basic idea of the algorithm is to use depth information to assign different weight coefficients to different areas of a tracking image. The closer the depth is to the previous depth of the target, the larger the weight coefficient, and vice versa. If the mean grayscale value of the area where the target was located in the depth image at the last moment is *MF*, and the grayscale value of the spot with coordinates (*x, y*) in the depth image *D* at this moment is *D*(*x, y*), then the weight coefficient *C*(*x, y*) at (*x, y*) is

(10) $$C\left(x,y\right)=\frac{1}{K\left|D\left(x,y\right)-MF\right|+1},$$

where *K* is the environmental coefficient, to which a value may be assigned according to the characteristics of the specific application because the larger *K* is, the more obviously the weight coefficient varies with the depth of the pixel location. Once the weight coefficient of each spot in the tracked image is available, the image to be tracked *G* (the data source) is weighted by the following formula:

(11) WG(x,y)=G(x,y)×C(x,y).

With this algorithm, using the method described in section Mean-Shift Target Tracking Method, *G* is replaced with a weighted grayscale, *WG*.

Compared with the traditional mean-shift target tracking method, this algorithm adds M × N addition operations, M × N subtraction operations, and 2 × M × N multiplication operations for one tracking (when the detected image size is M pixels × N pixels).

### Introducing Depth Information Into the Weighting of the Density Distribution of the Dataset

The basic idea of this algorithm is the same as that of the algorithm described in section Introducing Depth Information Into the Weighting of the Data Source. Once the weight coefficient of each spot in the tracking image is available based on Equation (11), the back projection *P* obtained by the method in section Introduction is weighted by the following formula:

(12) WP(x,y)=P(x,y)×C(x,y).

In this algorithm, using the method described in section Mean-Shift Target Tracking Method, *P* is replaced with the weighted back projection *WP*.

This algorithm requires the same additional computing resources as the method described in section Introducing Depth Information Into the Weighting of the Data Source.

## Experiment and Analysis

### Experiment Apparatus and Scenario

This study focuses on how to improve the performance of generative target tracking methods into which depth information is introduced; however, most of the existing mainstream datasets on the available target tracking do not provide depth information. Therefore, this article addresses how the proposed improvement methods are verified using test images acquired experimentally. The image acquisition apparatus for the experiment was a German camera (Basler tof640-20gm_ 850-nm type TOF) with a pixel resolution of 640 × 480, a depth measurement range of 0–13 m, and a measurement accuracy of ±10 mm. The tracking algorithm was developed and implemented on a PC (Intel Cool i5-4200u processor, 8 GB of RAM) operating on a VS2015. In the algorithm, the threshold of the window displacement distance ε at the end of the iterations is set to 1 pixel, and the threshold of the number of iterations *N* is 10.

**Figure 2:**
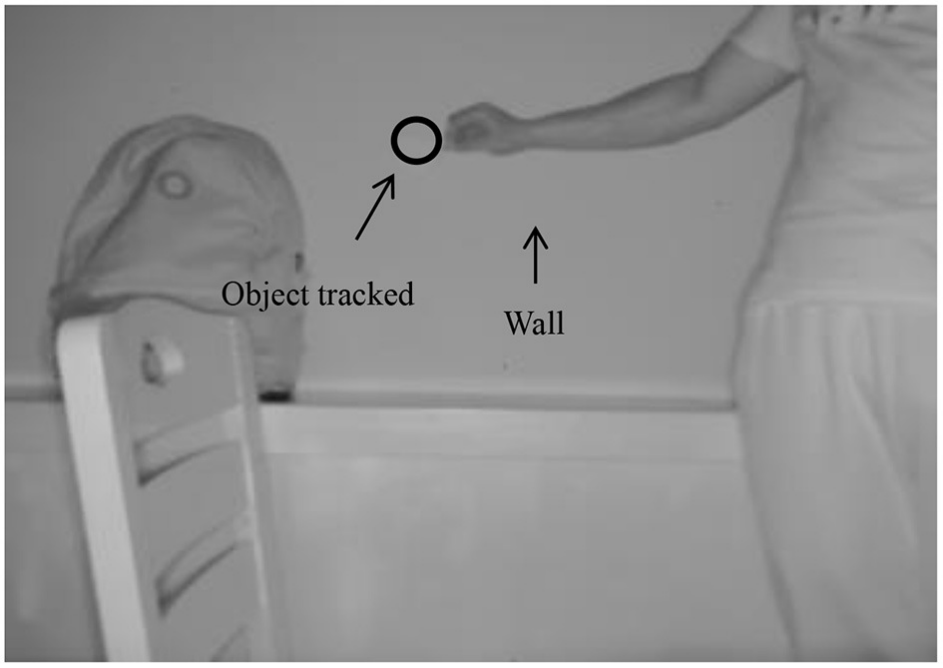
The 28th frame.

The experimental scenario was designed such that a white round cover acted as the object tracked in a colour extremely close to that of the wall, which can be regarded as a confusingly similar-looking background. To fully verify the robustness of the algorithm, during the experiment, the tracked object also had a significant displacement in the depth direction (the minimum distance between the tracked object and the wall at the initial moment was 104 mm, and then gradually increased to 209 mm). For the experiment, three-dimensional vision data at 81 moments were acquired on a continuous basis. The 28th frame of the grayscale image is shown in Figure 2, where the TOF camera is 1,462 mm away from the wall.

### Parameter Value and Method Validity

This section begins with a discussion of how the parameter values are assigned to each method. The high and low depth thresholds must be determined before introducing depth information into the thresholding of the data source (hereinafter, referred to as Method 1), and the depth information is introduced into the thresholding of the density distribution of the dataset (hereinafter, referred to as Method 2). The values are assigned to the two thresholds above based on the *priori* information of the displacement of the object tracked in the depth direction and its distance from the TOF camera under the application scenario. During this experiment, the distance between the TOF camera and the tracked object was between 1,253 and 1,358 mm. Considering that a certain margin is required in actual applications, distances of 1,044 and 1,462 mm are assigned to *T*_2_ and *T*_1_.

The environmental coefficient *K* must be determined before introducing the depth information into the weighting of the data source (hereinafter, referred to as Method 3) and introduces depth information into the weighting of the density distribution of the dataset (hereinafter, referred to as Method 4). After many attempts, it was found that a *K* value of ~1 in the designed experimental scenario is reasonable.

In addition, the mean-shift method and the four improvement methods in this study all require a quantisation level *m* of the histogram. The grayscale information of each pixel in the frames collected in the experiment is stored as uint8, which accommodates up to a total of 256 shades of grayscale, *m*, and thus the value can theoretically be taken from the interval (Nousi et al., 2020a; 256). Table 1 shows the validity of each method when 10 different values of *m* are within the theoretical interval. It can be seen that, for the listed values assigned to *m*, the original mean-shift algorithm is invalid, whereas the four improvement methods described in this article are effective, at least under certain conditions, and are capable of improving the validity of the basic algorithm to a certain extent; to be specific, Methods 2 and 4 are valid in all cases, and it is more practical to introduce depth information at the level of density distribution of the dataset because it is less difficult to adjust the parameters; in a number of cases of value assignment to *m*, Methods 1 and 3 are invalid, suggesting that the practicality of introducing depth information at the data source level is poor.

**Table 1:** The relationship between the value of parameter m and the validity of the method.

**Histogram** **quantization** **series**	**Method validity**				
	**Traditional** **Mean-shift**	**Method 1**	**Method 2**	**Method 3**	**Method 4**
2	Invalid	Invalid	Valid	Valid	Valid
19	Invalid	Valid	Valid	Valid	Valid
39	Invalid	Valid	Valid	Invalid	Valid
59	Invalid	Valid	Valid	Invalid	Valid
99	Invalid	Valid	Valid	Valid	Valid
150	Invalid	Invalid	Valid	Valid	Valid
199	Invalid	Invalid	Valid	Valid	Valid
219	Invalid	Invalid	Valid	Invalid	Valid
239	Invalid	Invalid	Valid	Invalid	Valid
256	Invalid	Invalid	Valid	Invalid	Valid

**Figure 3:**
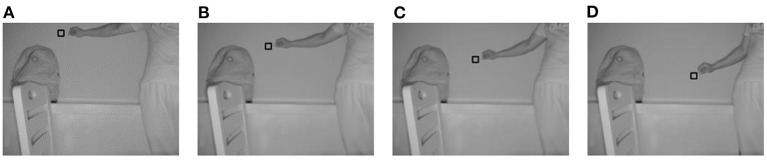
Tracking results from the method of thresholding the data source into which depth information is introduced. **(A)** The 10th frame; **(B)** the 30th frame; **(C)** the 50th frame; **(D)** the 80th frame.

**Figure 4:**
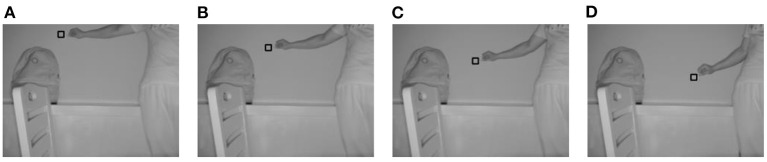
Tracking results from the method of thresholding the density distribution of the dataset into which depth information is introduced. **(A)** The 10th frame; **(B)** the 30th frame; **(C)** the 50th frame; **(D)** the 80th frame.

**Figure 5:**
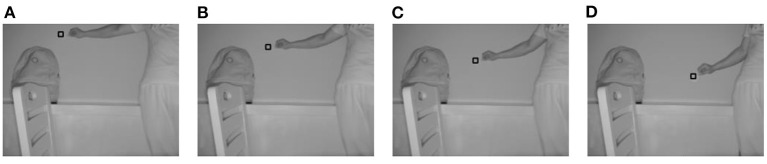
Tracking results from the method of weighting the data source into which depth information is introduced. **(A)** The 10th frame; **(B)** the 30th frame; **(C)** the 50th frame; **(D)** the 80th frame.

**Figure 6:**
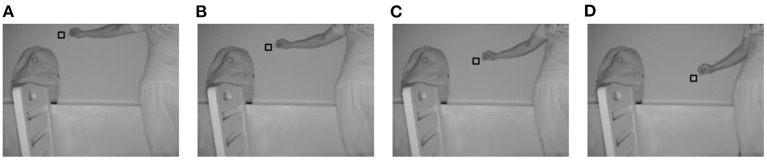
Tracking results from the method of weighting the density distribution of the dataset into which depth information is introduced. **(A)** The 10th frame; **(B)** the 30th frame; **(C)** the 50th frame; **(D)** the 80th frame.

**Figure 7:**
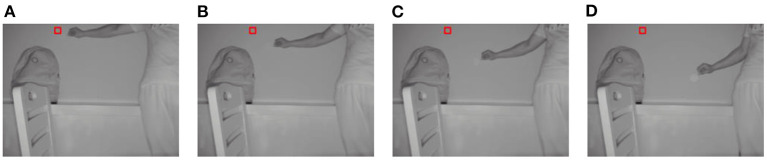
Tracking results from the mean-shift algorithm. **(A)** The 10th frame; **(B)** the 30th frame; **(C)** the 50th frame; **(D)** the 80th frame.

When *m* is set to 19, the four improvements in this study are valid. For a convenient comparison of the algorithm during the subsequent part of the experiment, *m* was set to 19 on a unified basis for all methods. Figures 3–6 show the tracking effects of the four improvement methods, and Figure 7 shows those of the traditional mean-shift algorithm.

### Real-Timeliness of Tracking

The mean times spent on the single tracking tasks by the traditional mean-shift algorithm and by the four improved methods based on the conducted experiment are shown in Table 2. The mean time in the case of the mean-shift algorithm is 16.9 ms, whereas those in the case of the four improved algorithms herein are higher because they involve more calculations. Methods 1 and 2 spent similar times on average, i.e., ~50% more than the standard algorithm. Methods 3 and 4 require a similar amount of time on average, much longer than the first two methods, and more than twice the standard algorithm. Nevertheless, even the longest time-consuming method, which requires 35.5 ms for the four single tracking tasks, can still meet the real-time requirement for most applications.

**Table 2:** Time consumed by each method in single tracking tasks.

**Method names**	**Time-consuming** **(ms)**
Mean-shift	16.9
Method 1	26.1
Method 2	25.5
Method 3	34.1
Method 4	35.5

### Tracking Accuracy

**Figure 8:**
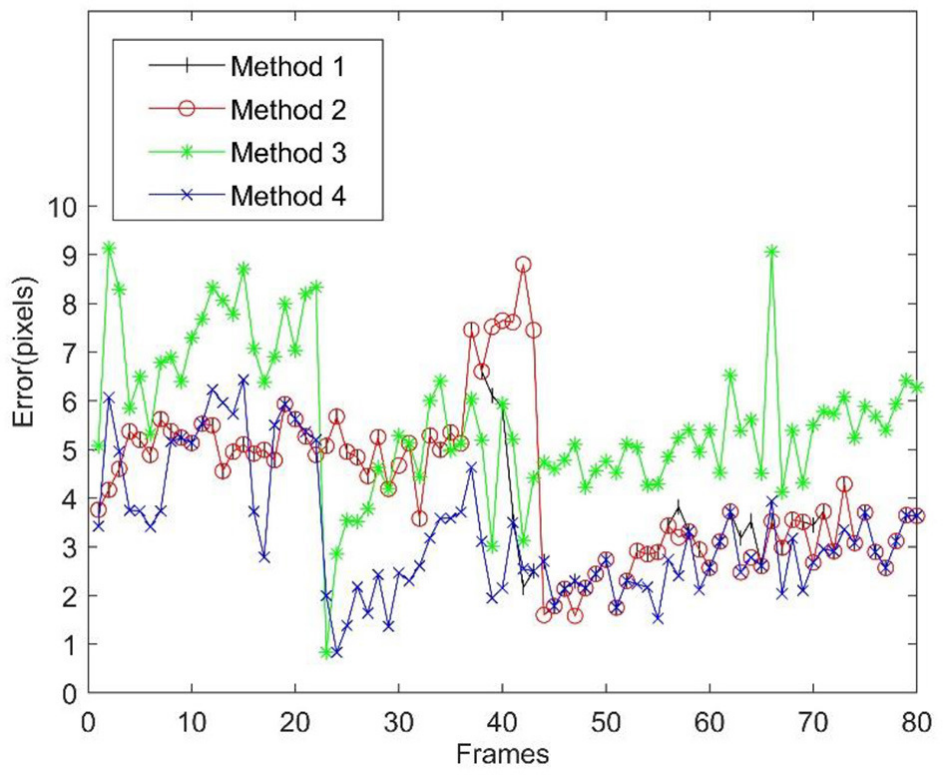
Tracking errors of improvement algorithm.

It was impossible to accurately locate the target during the experiment. To quantitatively evaluate the tracking accuracy, the accuracy evaluation system proposed in Hoover et al. (1996) is adopted in this study to manually capture the target edge and determine the position of the target centre (Wang et al., 2018) using the least square method as the true value. The tracking error is then identified as the distance between the centre position of the target obtained by each tracking method and the true value. The tracking error at each moment is shown in Figure 8, and the error statistics are listed in Table 3. It is clear from Figure 8 and Table 3 that Method 4 achieves the highest tracking accuracy, with a peak error of 6.43 pixels, followed by Method 2 and Method 1. Method 3, with the lowest tracking accuracy, had a peak error of 9.14 pixels, a mean absolute error (MAE) of 5.61 pixels, and a ratio of peak error to target size of <34% (the pixel size of the target in the experiment image varies with the distance between the target and camera, and ranges from 27.3 to 34.2 pixels in terms of diameter); however, the method ensures that the target tracked will not be lost.

**Table 3:** Tracking error statistics (unit: pixel).

**Method** **names**	**Peak** **error**	**Error standard** **deviation**	**Mean absolute** **error**
Method 1	7.46	1.27	4.08
Method 2	8.80	1.56	4.25
Method 3	9.14	1.52	5.61
Method 4	6.43	1.32	3.28

### Comparison of Comprehensive Performance

The comprehensive performance and characteristics of each improvement method are summarised in Table 4. Method 4, with an excellent performance in terms of the difficulty in parameter adjustment and the tracking accuracy, albeit with a higher time consumption, is the prime choice in engineering practise. Method 3 achieves the lowest tracking accuracy and has no obvious advantages. Methods 1 and 2 consume less time and are therefore suitable for occasions in which there is little confusion at the same depth of the target and a high requirement for real timeliness.

**Table 4:** Comparison of the comprehensive performance of the improvement methods.

**Method** **names**	**Difficulty of** **parameter** **adjustment**	**Time-consuming**	**Tracking** **accuracy**
Method 1	Higher	Less	Higher
Method 2	Low	Less	Higher
Method 3	Higher	More	Minimum
Method 4	Low	More	Highest

### Comparison With the State-of-the-Art Tracker

**Figure 9:**
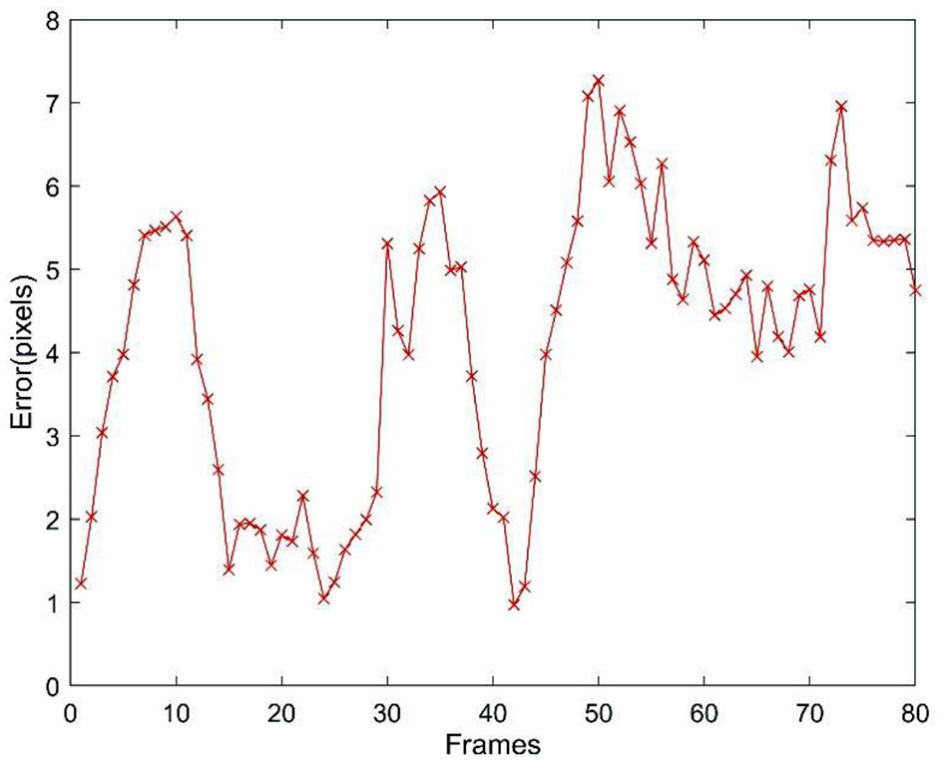
Tracking errors of the SiamFC network.

Finally, to further verify the effectiveness of the proposed methods, a comparison was conducted with the latest method, i.e., a fully convolutional siamese (SiamFC) approach (Gao et al., 2019), which is an object tracking method based on deep learning. Because the hardware platform running the mean-shift and improved algorithms described in this paper has a low configuration and cannot realise the training and tracking of the network, it was replaced by a mobile workstation with the following specifications: an i5-11400H CPU, 16 GB of RAM, and an RTX 3050 GPU using python3.6 and PyTorch. Then, the GOT-10k dataset was used to finish the training of the SiamFC network, and the error curve of the target tracking for the image sequence mentioned in section Experiment Apparatus and Scenario is shown in Figure 9. The peak error is 7.27 pixels and the MAE is 4.11 pixels. The tracking accuracy of the SiamFC network is similar to that of Method 1, but lower than that of Method 4. In addition, the mean tracking time of 81 frames is 19.6 ms, which is slightly higher than that of the four improved algorithms. Because the configuration of the hardware platform running the SiamFC network is obviously better than that running the improved algorithm, a slightly higher time consumption does not mean that the improved algorithms require more computing resources. The improved algorithms, which use significantly lower hardware configurations, also obtain a roughly equivalent tracking performance (in terms of the tracking accuracy, some of the improved methods proposed in this paper are even higher) to the state-of-the-art tracker under the designed experimental scenario; therefore, the improved methods described in this paper have practical value.

## Conclusion

Generative target tracking algorithms represented by the mean-shift improve independence on training samples, easy implementation and real-timeliness etc. and get widely applied in engineering practise. However, these methods were developed before 3D visual information acquisition apparatuses could be put into popular use. Therefore, common visual features used in target tracking, only including colour and grayscale, and visual features derived from these two types of information, are prone to failure in a confusingly similar-looking background. To deal with the problem above, this study, based on the mean-shift, a typical framework for generative target tracking, proposes four improved methods into which depth information is introduced, i.e., the thresholding of the data source, thresholding of the density distribution of the dataset applied, weighting of the data source, and weighting of the density distribution of the dataset. The experimental study includes a detailed analysis of the parameter values, validity, tracking real-timeliness and accuracy of each method. The experimental results showed that the four methods can improve the validity of the basic method to a certain extent and meet the requirements of real-time target tracking in a confusingly similar background. The method of weighting the density distribution of the dataset, into which depth information is introduced, is the prime choice in engineering practise because it delivers an excellent comprehensive performance and the highest level of accuracy, whereas methods such as the thresholding of both the data sources and the density distribution of the dataset are less time-consuming, therefore more suitable for occasions where there is little confusion at the same depth of the target and a high requirement for real-timeliness. The performance in comparison with that of a state-of-the-art tracker further verifies the practicality of the proposed approach. Finally, the research results also provide a reference for improvements in other target tracking methods in which depth information can be introduced.

In the target tracking framework of this study, the essence of the mean-shift algorithm is actually data clustering. Some new clustering algorithms (Hu et al., 2019, 2020) also have the potential to be applied to target tracking. The next step is to further improve the performance of target tracking in light of the latest research progress of clustering techniques.

## Data Availability Statement

The original contributions presented in the study are included in the article/Supplementary Material, further inquiries can be directed to the corresponding author.

## Author Contributions

DS: writing—original draft, data curation, and visualization. XW: supervision, conceptualization, writing—review, and editing. YL: methodology, software, investigation, and writing—original draft. TY: validation. SW: resources. All authors contributed to the article and approved the submitted version.

## Conflict of Interest

SW is employed by Changsha Shi-Lang Technology Co. The remaining authors declare that the research was conducted in the absence of any commercial or financial relationships that could be construed as a potential conflict of interest.

## Publisher's Note

All claims expressed in this article are solely those of the authors and do not necessarily represent those of their affiliated organizations, or those of the publisher, the editors and the reviewers. Any product that may be evaluated in this article, or claim that may be made by its manufacturer, is not guaranteed or endorsed by the publisher.
