# Supplementary figures and images for: Introducing Depth Information Into Generative Target Tracking (part 2 of 2)
Source: Front Neurorobot. 2021 Sep 1;15:718681. doi: 10.3389/fnbot.2021.718681 (PMC8442731; doi:10.3389/fnbot.2021.718681)

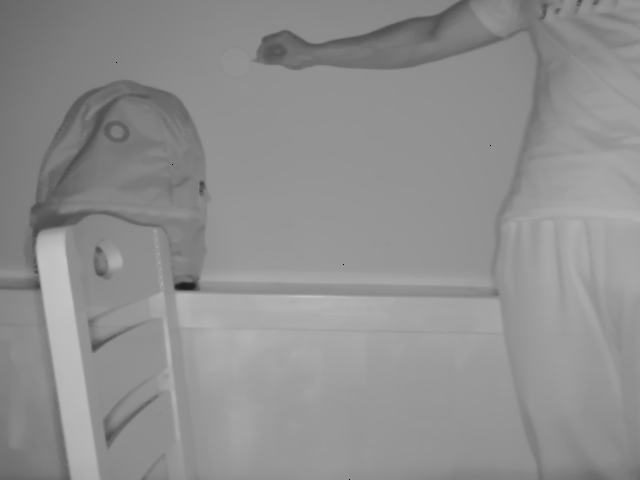

Supplement: Supplementary file 1 [file Data_Sheet_1.ZIP › The experimental data/grayscale image/tof640-20gm-22543413-0020-intensity.png]

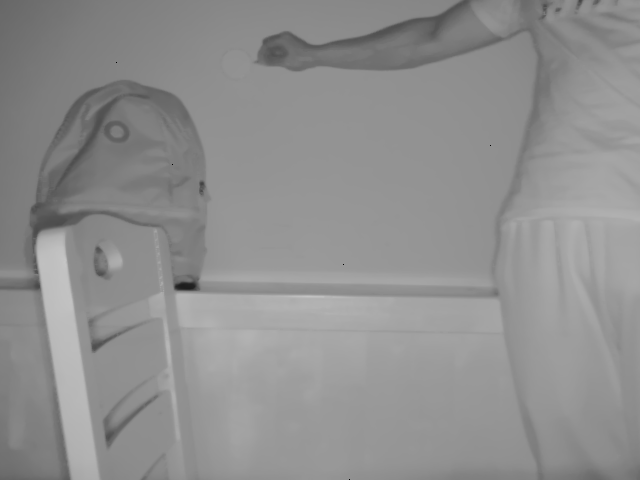

Supplement: Supplementary file 1 [file Data_Sheet_1.ZIP › The experimental data/grayscale image/tof640-20gm-22543413-0021-intensity.png]

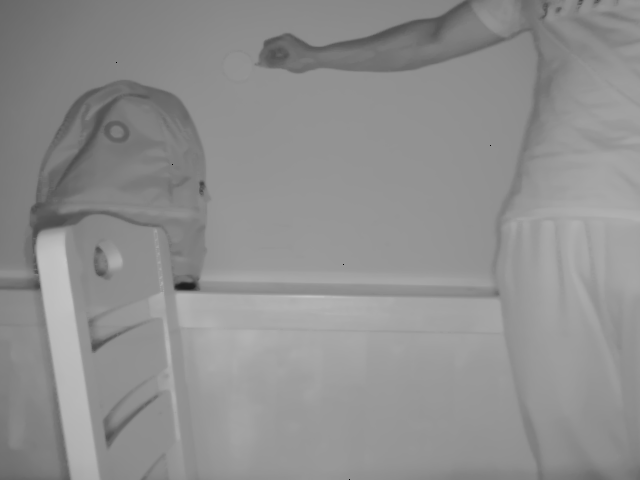

Supplement: Supplementary file 1 [file Data_Sheet_1.ZIP › The experimental data/grayscale image/tof640-20gm-22543413-0022-intensity.png]

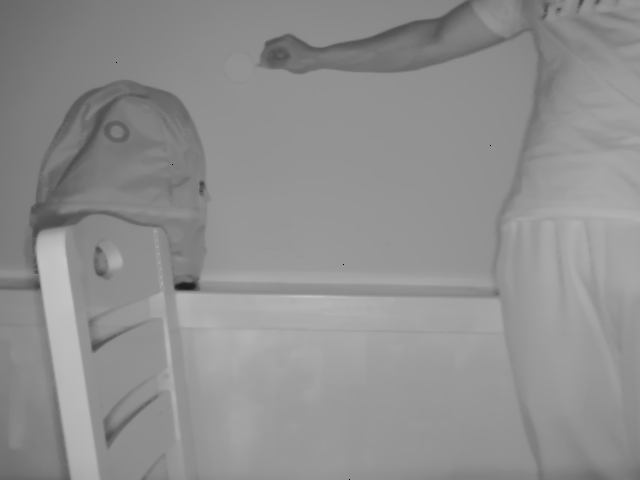

Supplement: Supplementary file 1 [file Data_Sheet_1.ZIP › The experimental data/grayscale image/tof640-20gm-22543413-0023-intensity.png]

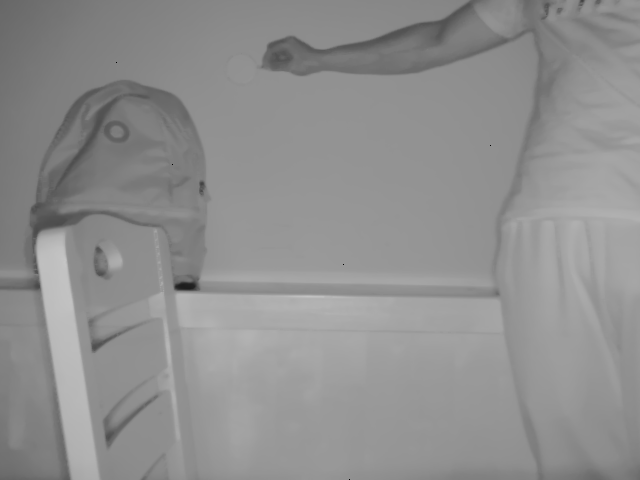

Supplement: Supplementary file 1 [file Data_Sheet_1.ZIP › The experimental data/grayscale image/tof640-20gm-22543413-0024-intensity.png]

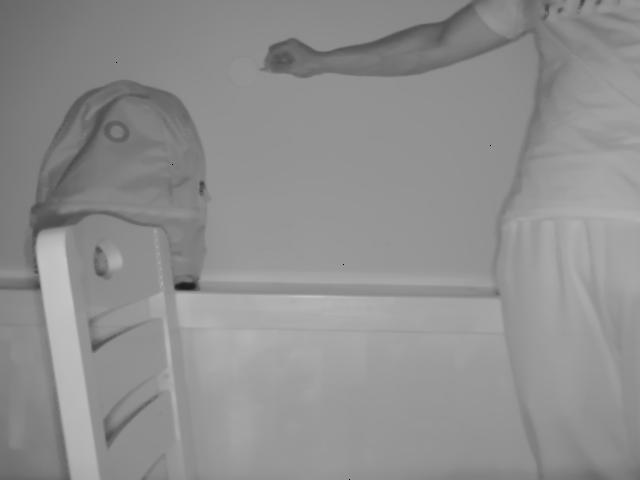

Supplement: Supplementary file 1 [file Data_Sheet_1.ZIP › The experimental data/grayscale image/tof640-20gm-22543413-0025-intensity.png]

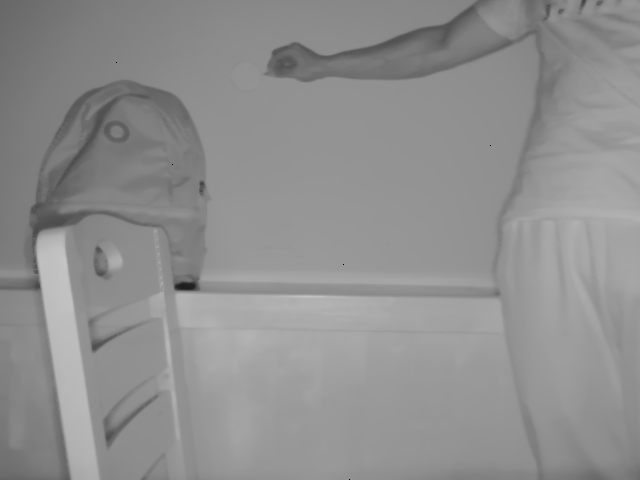

Supplement: Supplementary file 1 [file Data_Sheet_1.ZIP › The experimental data/grayscale image/tof640-20gm-22543413-0026-intensity.png]

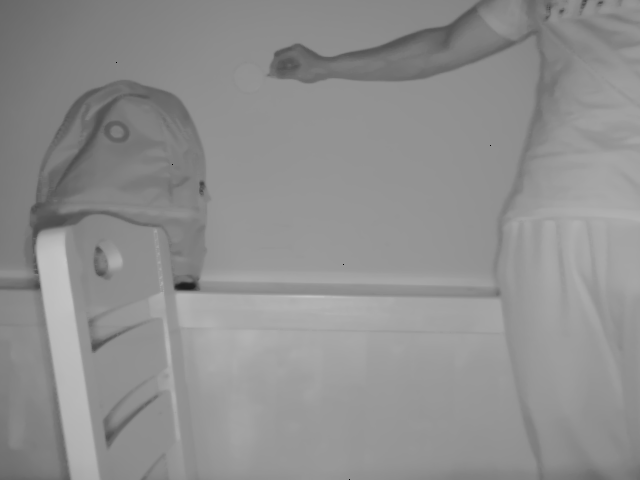

Supplement: Supplementary file 1 [file Data_Sheet_1.ZIP › The experimental data/grayscale image/tof640-20gm-22543413-0027-intensity.png]

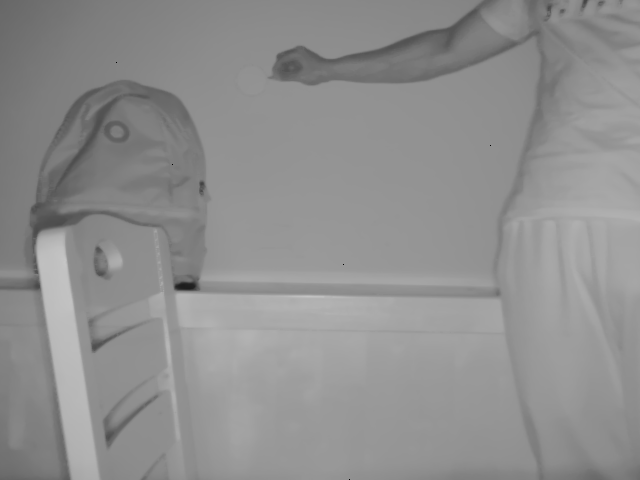

Supplement: Supplementary file 1 [file Data_Sheet_1.ZIP › The experimental data/grayscale image/tof640-20gm-22543413-0028-intensity.png]

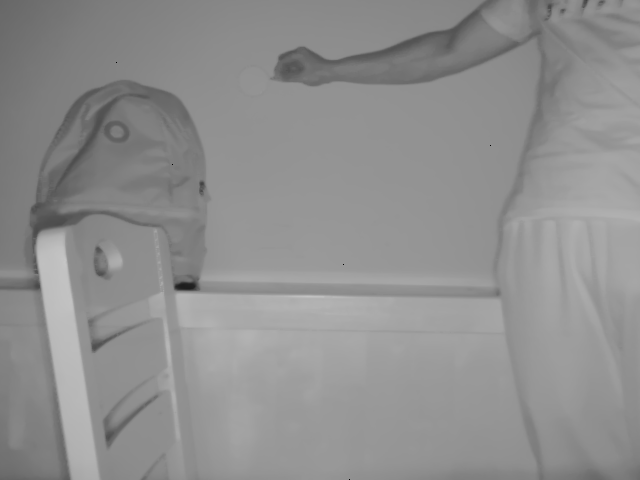

Supplement: Supplementary file 1 [file Data_Sheet_1.ZIP › The experimental data/grayscale image/tof640-20gm-22543413-0029-intensity.png]

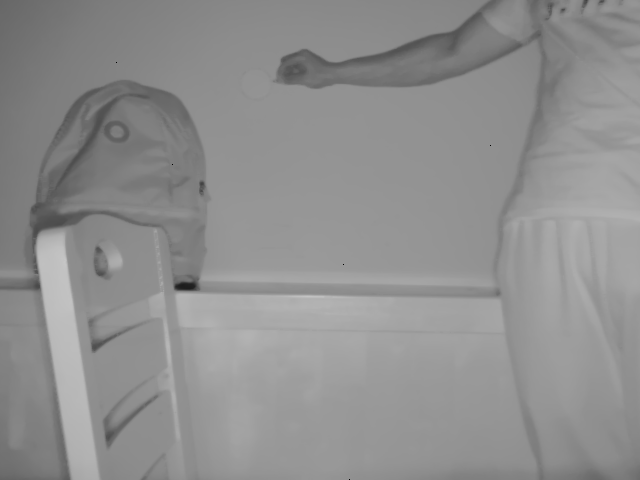

Supplement: Supplementary file 1 [file Data_Sheet_1.ZIP › The experimental data/grayscale image/tof640-20gm-22543413-0030-intensity.png]

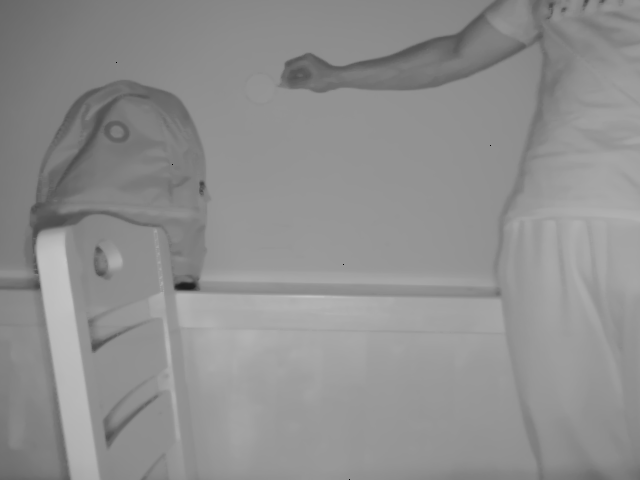

Supplement: Supplementary file 1 [file Data_Sheet_1.ZIP › The experimental data/grayscale image/tof640-20gm-22543413-0031-intensity.png]

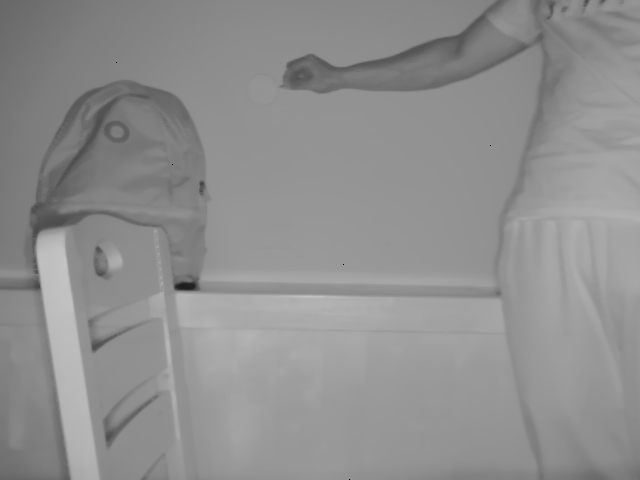

Supplement: Supplementary file 1 [file Data_Sheet_1.ZIP › The experimental data/grayscale image/tof640-20gm-22543413-0032-intensity.png]

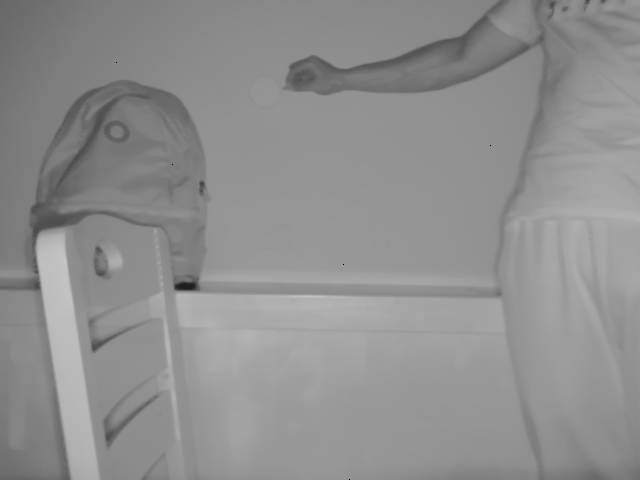

Supplement: Supplementary file 1 [file Data_Sheet_1.ZIP › The experimental data/grayscale image/tof640-20gm-22543413-0033-intensity.png]

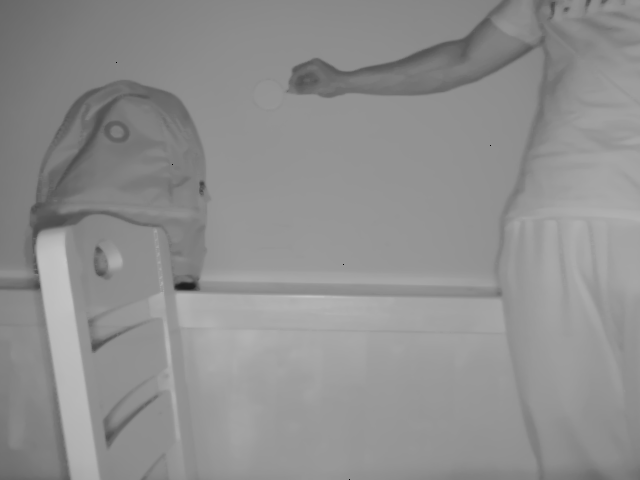

Supplement: Supplementary file 1 [file Data_Sheet_1.ZIP › The experimental data/grayscale image/tof640-20gm-22543413-0034-intensity.png]

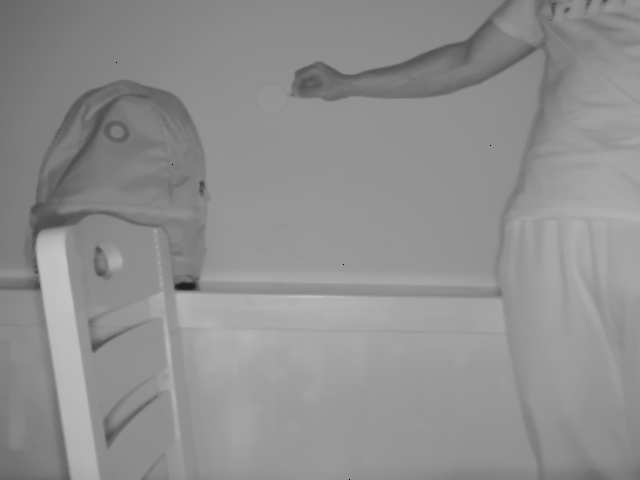

Supplement: Supplementary file 1 [file Data_Sheet_1.ZIP › The experimental data/grayscale image/tof640-20gm-22543413-0035-intensity.png]

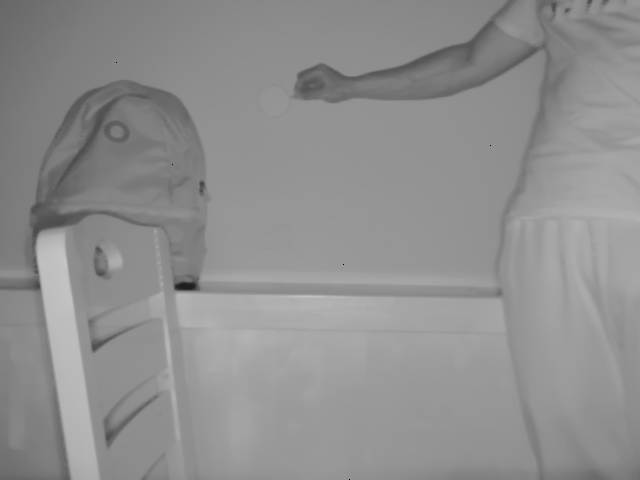

Supplement: Supplementary file 1 [file Data_Sheet_1.ZIP › The experimental data/grayscale image/tof640-20gm-22543413-0036-intensity.png]

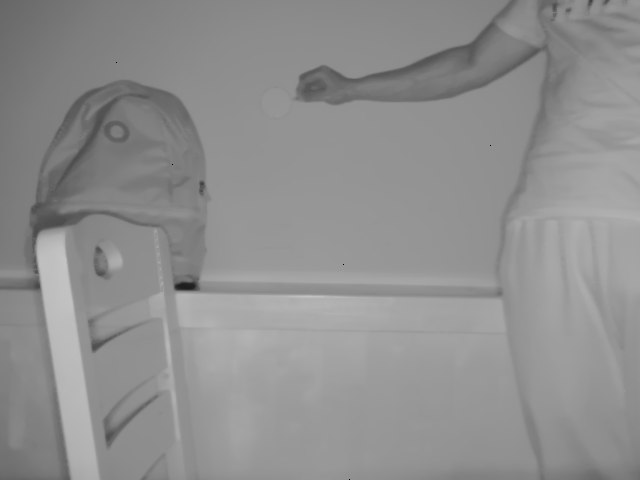

Supplement: Supplementary file 1 [file Data_Sheet_1.ZIP › The experimental data/grayscale image/tof640-20gm-22543413-0037-intensity.png]

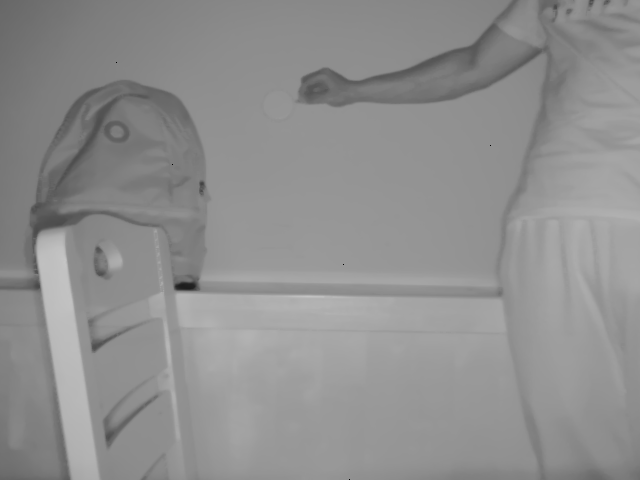

Supplement: Supplementary file 1 [file Data_Sheet_1.ZIP › The experimental data/grayscale image/tof640-20gm-22543413-0038-intensity.png]

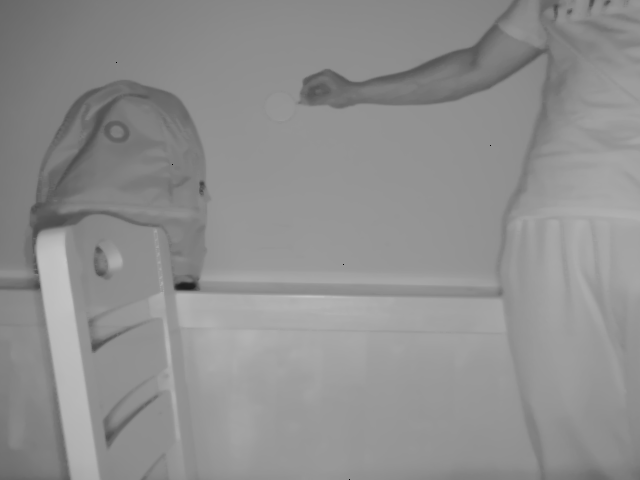

Supplement: Supplementary file 1 [file Data_Sheet_1.ZIP › The experimental data/grayscale image/tof640-20gm-22543413-0039-intensity.png]

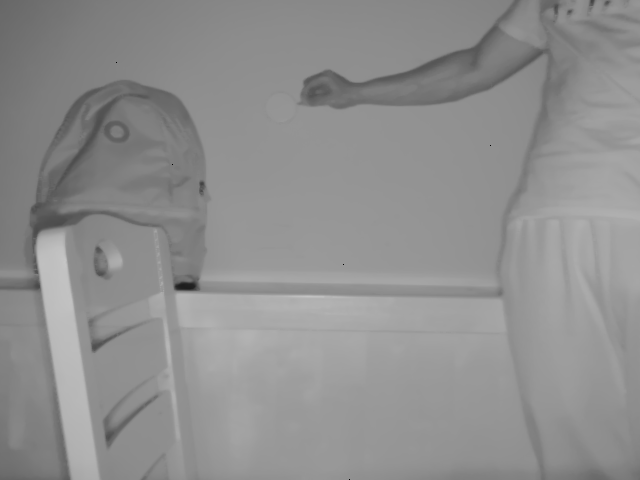

Supplement: Supplementary file 1 [file Data_Sheet_1.ZIP › The experimental data/grayscale image/tof640-20gm-22543413-0040-intensity.png]

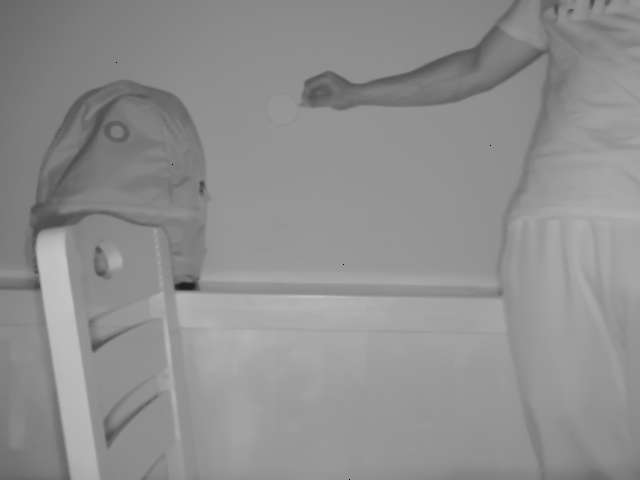

Supplement: Supplementary file 1 [file Data_Sheet_1.ZIP › The experimental data/grayscale image/tof640-20gm-22543413-0041-intensity.png]

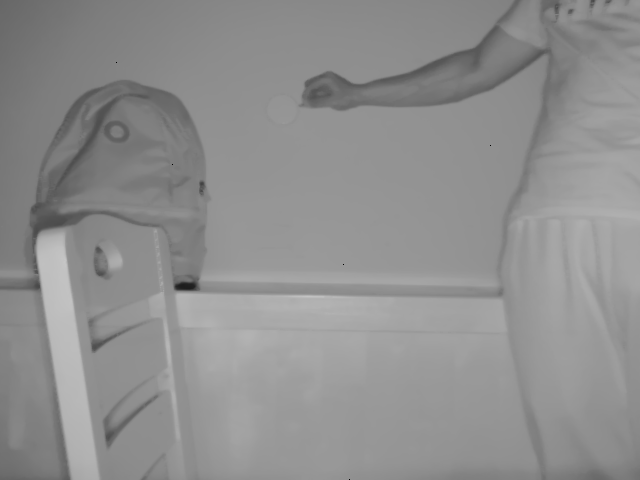

Supplement: Supplementary file 1 [file Data_Sheet_1.ZIP › The experimental data/grayscale image/tof640-20gm-22543413-0042-intensity.png]

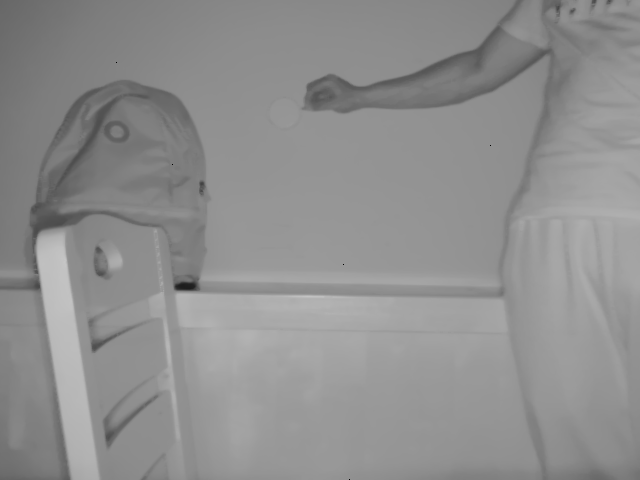

Supplement: Supplementary file 1 [file Data_Sheet_1.ZIP › The experimental data/grayscale image/tof640-20gm-22543413-0043-intensity.png]

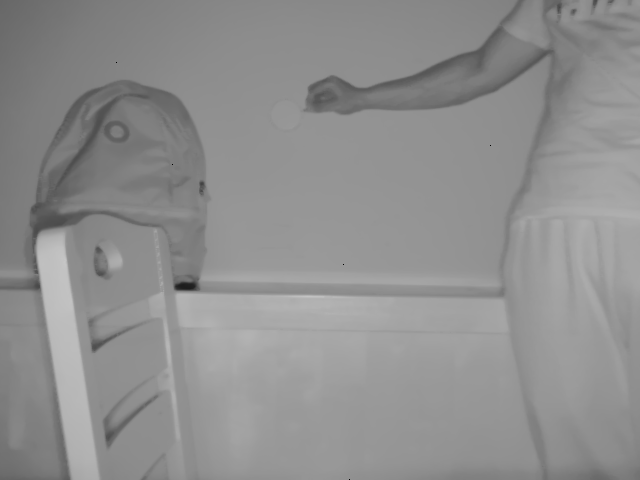

Supplement: Supplementary file 1 [file Data_Sheet_1.ZIP › The experimental data/grayscale image/tof640-20gm-22543413-0044-intensity.png]

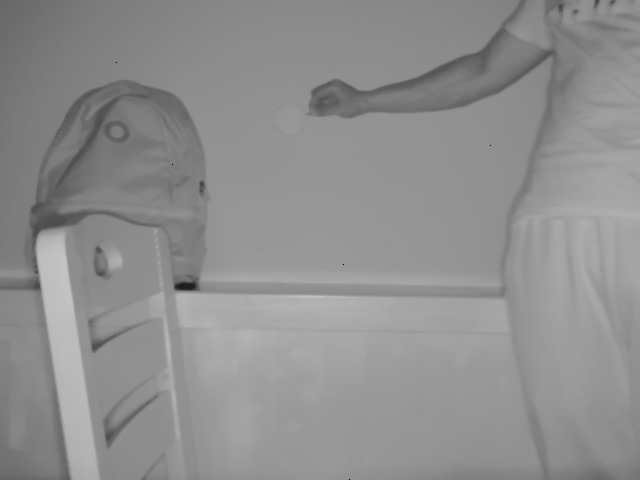

Supplement: Supplementary file 1 [file Data_Sheet_1.ZIP › The experimental data/grayscale image/tof640-20gm-22543413-0045-intensity.png]

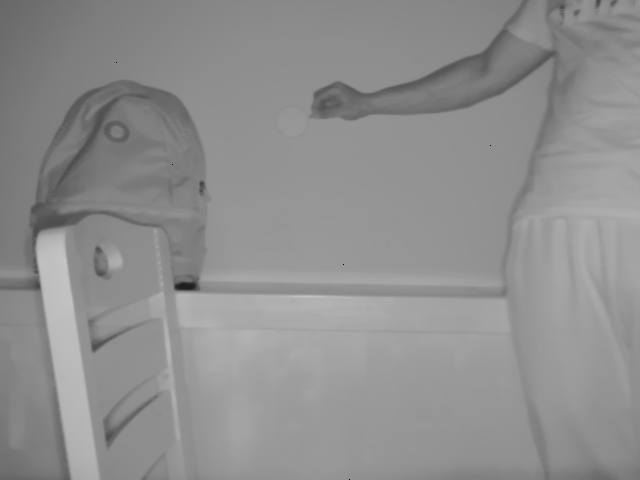

Supplement: Supplementary file 1 [file Data_Sheet_1.ZIP › The experimental data/grayscale image/tof640-20gm-22543413-0046-intensity.png]

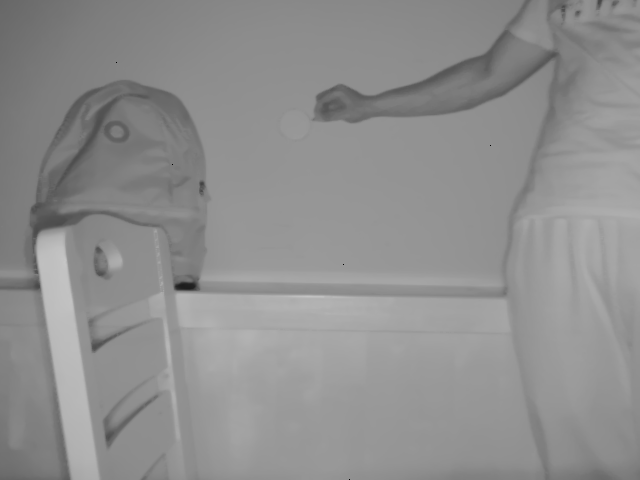

Supplement: Supplementary file 1 [file Data_Sheet_1.ZIP › The experimental data/grayscale image/tof640-20gm-22543413-0047-intensity.png]

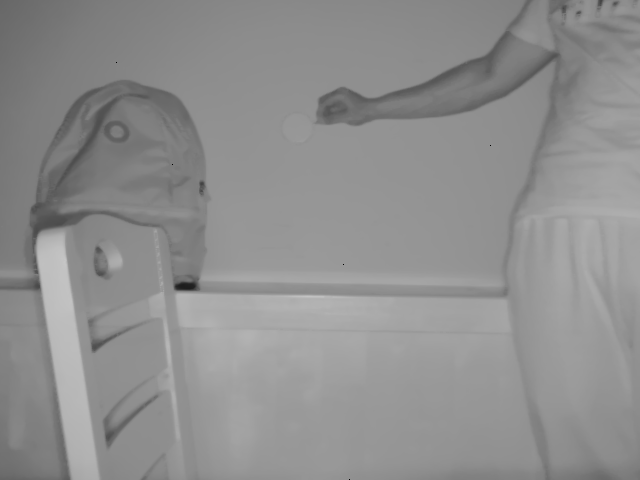

Supplement: Supplementary file 1 [file Data_Sheet_1.ZIP › The experimental data/grayscale image/tof640-20gm-22543413-0048-intensity.png]

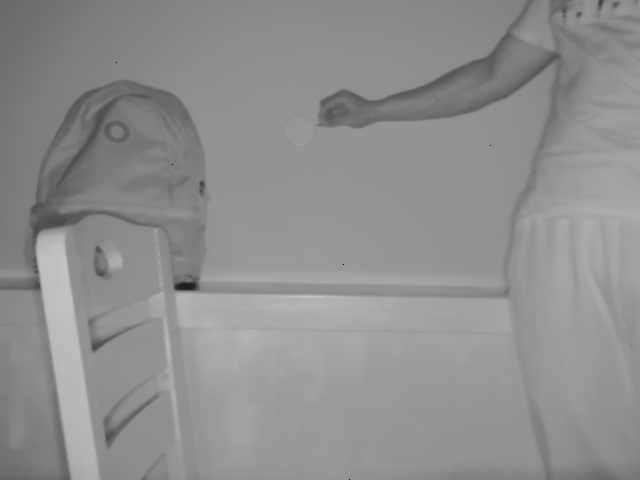

Supplement: Supplementary file 1 [file Data_Sheet_1.ZIP › The experimental data/grayscale image/tof640-20gm-22543413-0049-intensity.png]

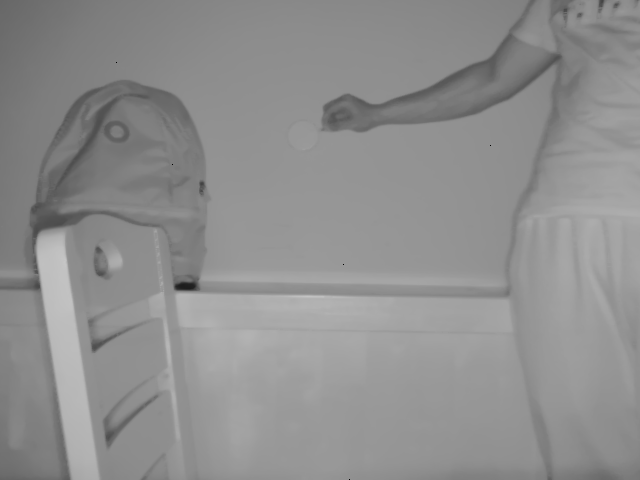

Supplement: Supplementary file 1 [file Data_Sheet_1.ZIP › The experimental data/grayscale image/tof640-20gm-22543413-0050-intensity.png]

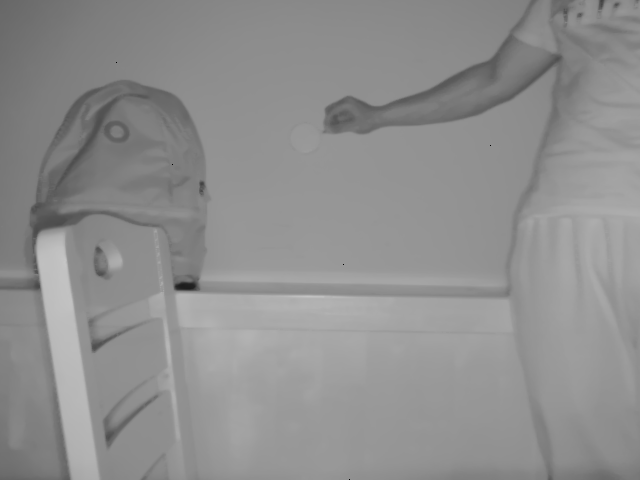

Supplement: Supplementary file 1 [file Data_Sheet_1.ZIP › The experimental data/grayscale image/tof640-20gm-22543413-0051-intensity.png]

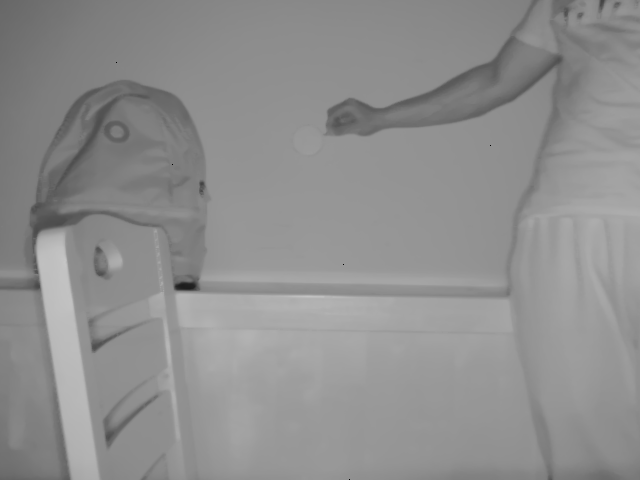

Supplement: Supplementary file 1 [file Data_Sheet_1.ZIP › The experimental data/grayscale image/tof640-20gm-22543413-0052-intensity.png]

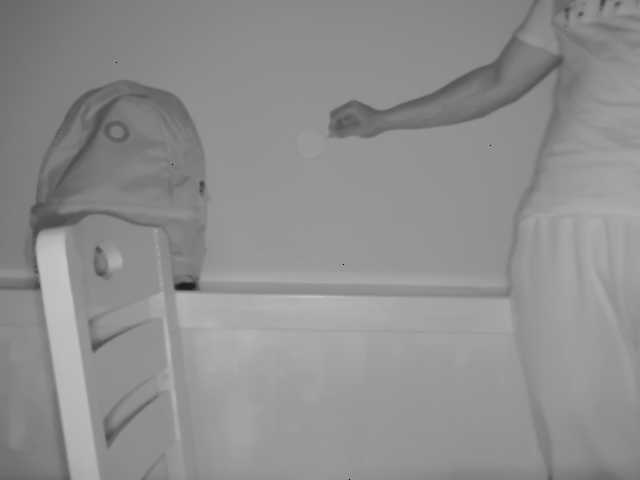

Supplement: Supplementary file 1 [file Data_Sheet_1.ZIP › The experimental data/grayscale image/tof640-20gm-22543413-0053-intensity.png]

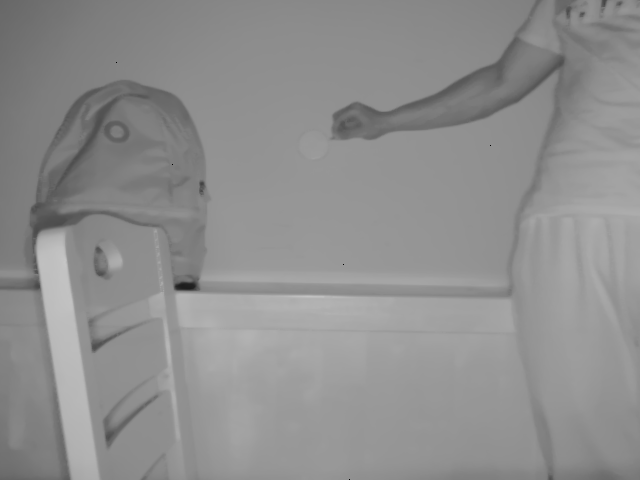

Supplement: Supplementary file 1 [file Data_Sheet_1.ZIP › The experimental data/grayscale image/tof640-20gm-22543413-0054-intensity.png]

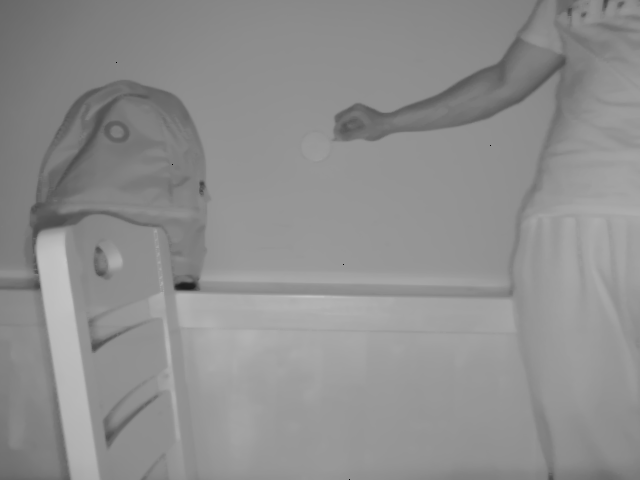

Supplement: Supplementary file 1 [file Data_Sheet_1.ZIP › The experimental data/grayscale image/tof640-20gm-22543413-0055-intensity.png]

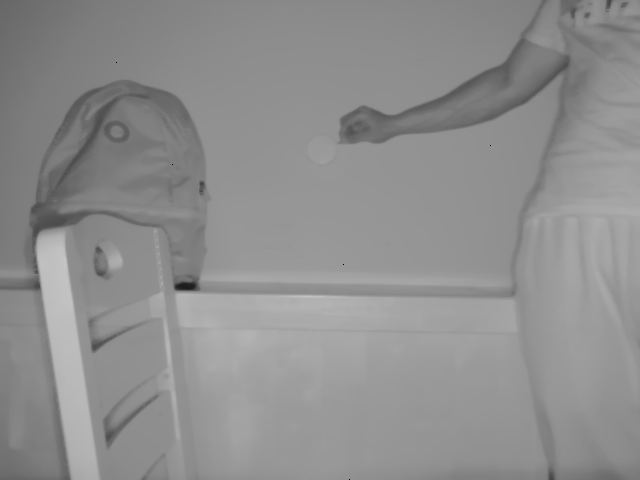

Supplement: Supplementary file 1 [file Data_Sheet_1.ZIP › The experimental data/grayscale image/tof640-20gm-22543413-0056-intensity.png]

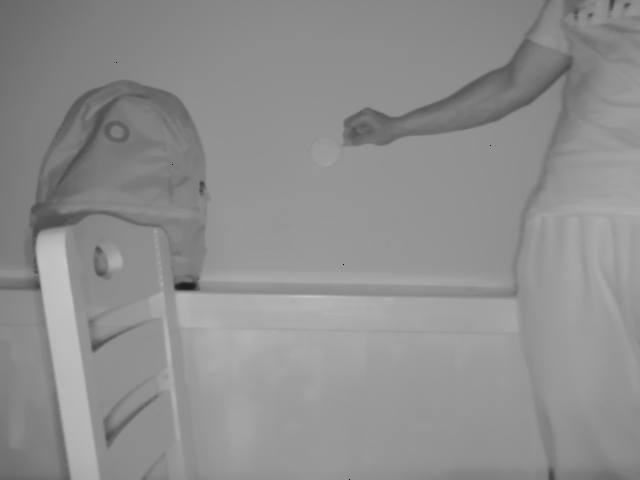

Supplement: Supplementary file 1 [file Data_Sheet_1.ZIP › The experimental data/grayscale image/tof640-20gm-22543413-0057-intensity.png]

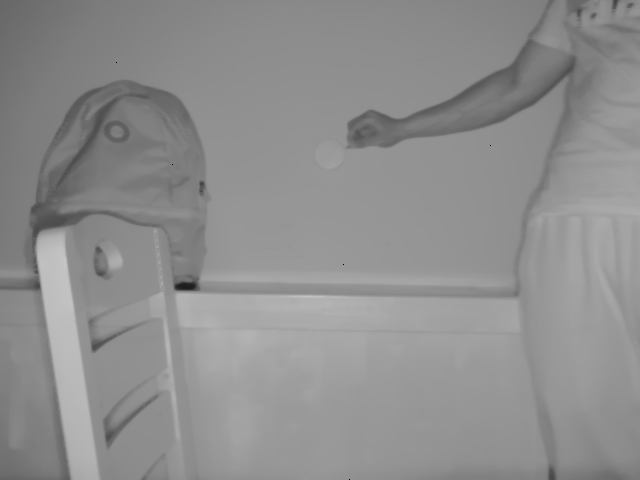

Supplement: Supplementary file 1 [file Data_Sheet_1.ZIP › The experimental data/grayscale image/tof640-20gm-22543413-0058-intensity.png]

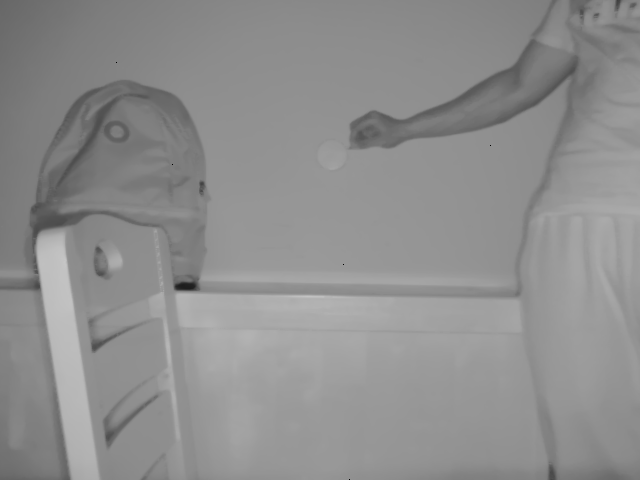

Supplement: Supplementary file 1 [file Data_Sheet_1.ZIP › The experimental data/grayscale image/tof640-20gm-22543413-0059-intensity.png]

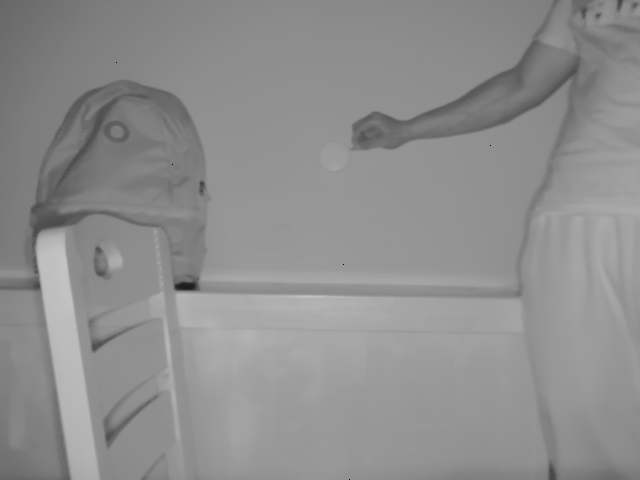

Supplement: Supplementary file 1 [file Data_Sheet_1.ZIP › The experimental data/grayscale image/tof640-20gm-22543413-0060-intensity.png]

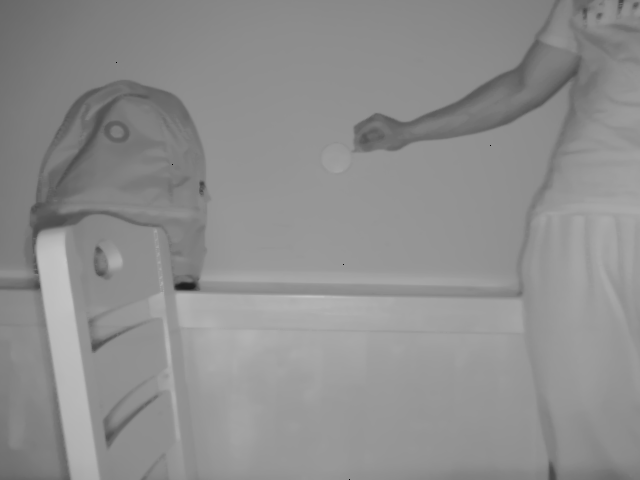

Supplement: Supplementary file 1 [file Data_Sheet_1.ZIP › The experimental data/grayscale image/tof640-20gm-22543413-0061-intensity.png]

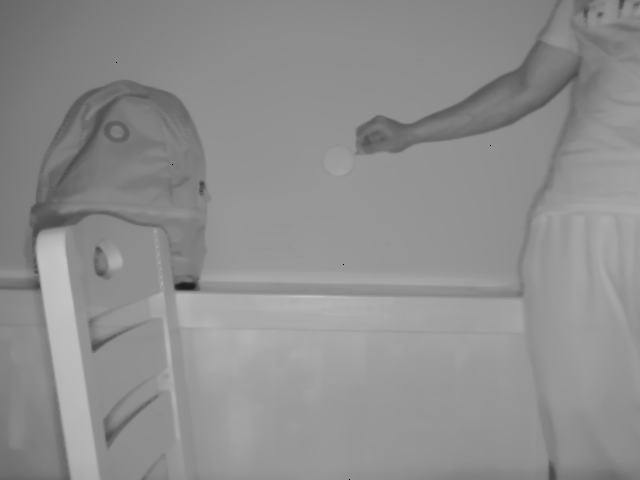

Supplement: Supplementary file 1 [file Data_Sheet_1.ZIP › The experimental data/grayscale image/tof640-20gm-22543413-0062-intensity.png]

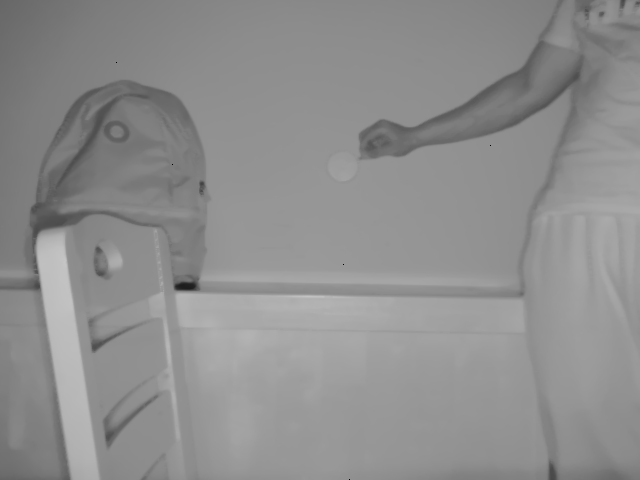

Supplement: Supplementary file 1 [file Data_Sheet_1.ZIP › The experimental data/grayscale image/tof640-20gm-22543413-0063-intensity.png]

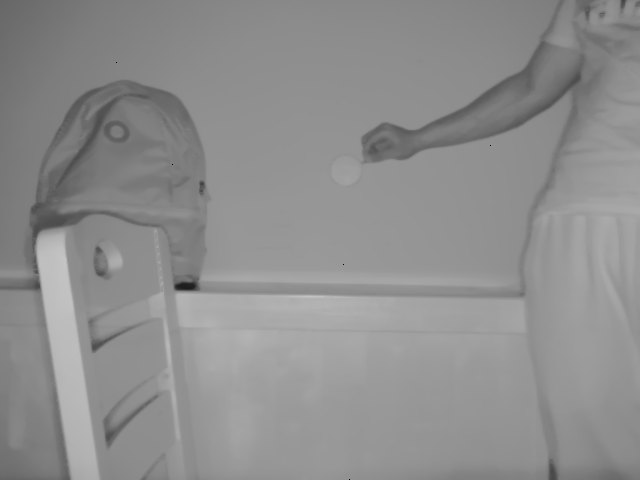

Supplement: Supplementary file 1 [file Data_Sheet_1.ZIP › The experimental data/grayscale image/tof640-20gm-22543413-0064-intensity.png]

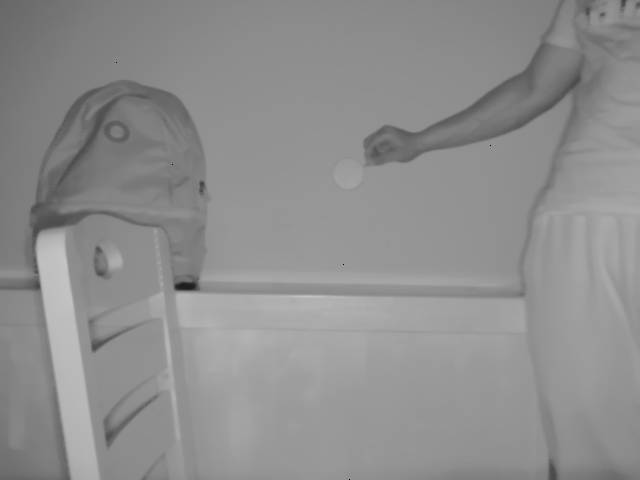

Supplement: Supplementary file 1 [file Data_Sheet_1.ZIP › The experimental data/grayscale image/tof640-20gm-22543413-0065-intensity.png]

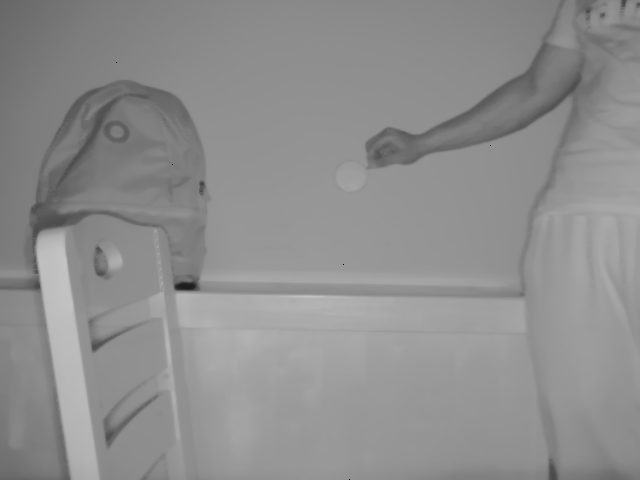

Supplement: Supplementary file 1 [file Data_Sheet_1.ZIP › The experimental data/grayscale image/tof640-20gm-22543413-0066-intensity.png]

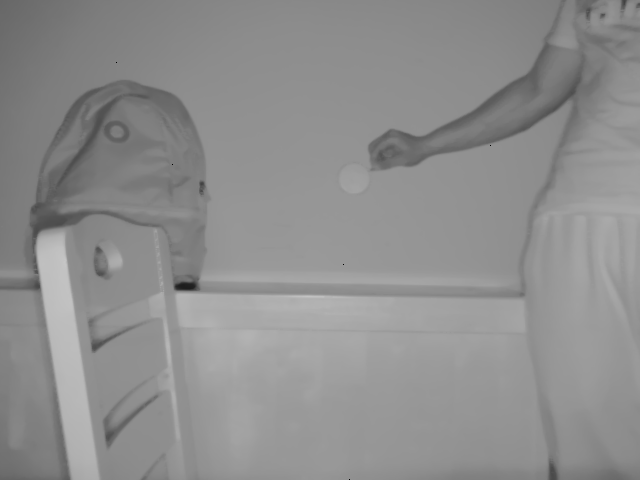

Supplement: Supplementary file 1 [file Data_Sheet_1.ZIP › The experimental data/grayscale image/tof640-20gm-22543413-0067-intensity.png]

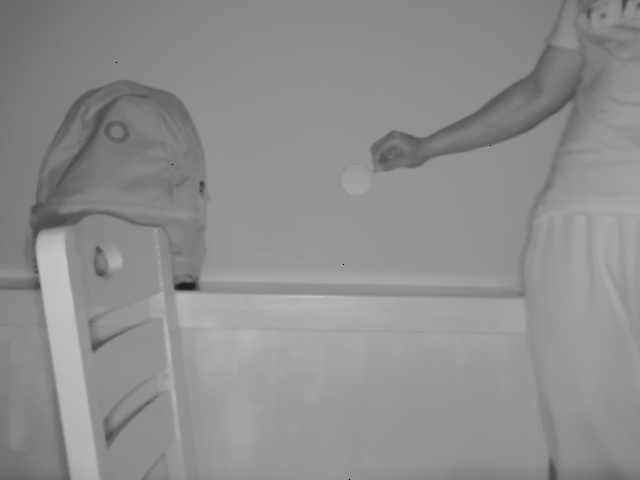

Supplement: Supplementary file 1 [file Data_Sheet_1.ZIP › The experimental data/grayscale image/tof640-20gm-22543413-0068-intensity.png]

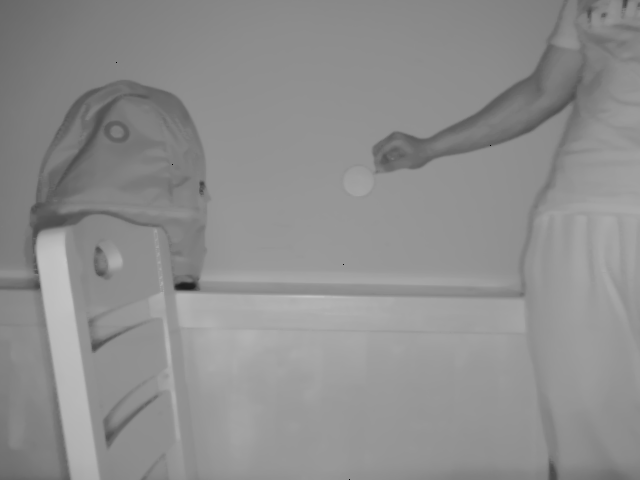

Supplement: Supplementary file 1 [file Data_Sheet_1.ZIP › The experimental data/grayscale image/tof640-20gm-22543413-0069-intensity.png]

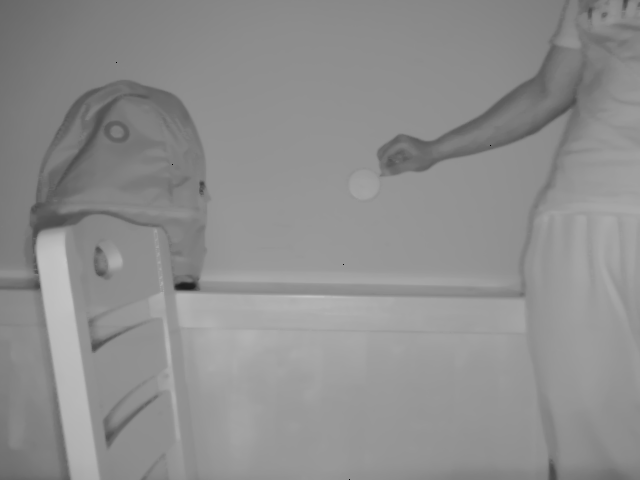

Supplement: Supplementary file 1 [file Data_Sheet_1.ZIP › The experimental data/grayscale image/tof640-20gm-22543413-0070-intensity.png]

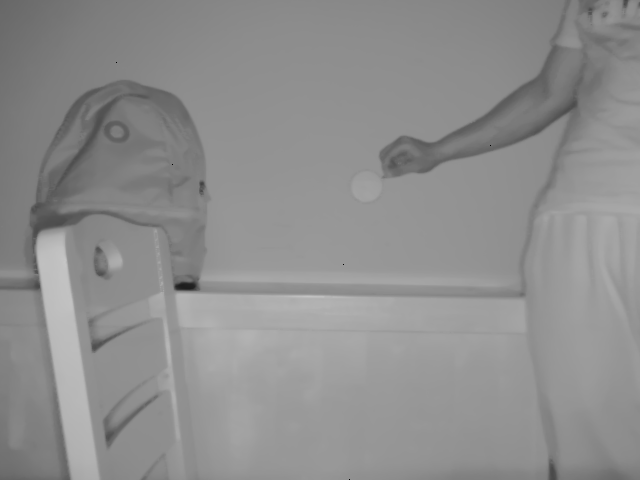

Supplement: Supplementary file 1 [file Data_Sheet_1.ZIP › The experimental data/grayscale image/tof640-20gm-22543413-0071-intensity.png]

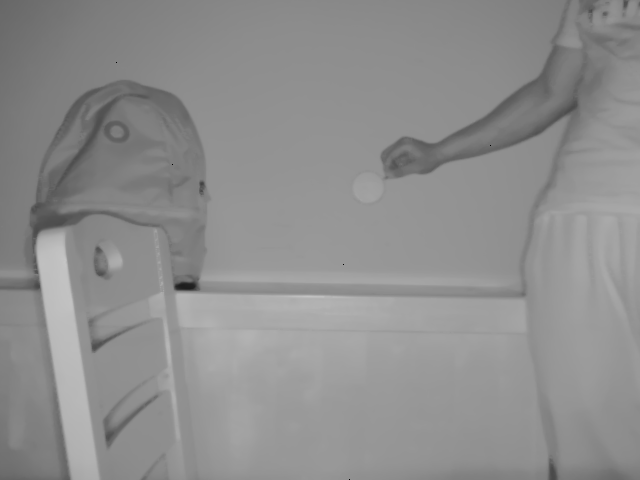

Supplement: Supplementary file 1 [file Data_Sheet_1.ZIP › The experimental data/grayscale image/tof640-20gm-22543413-0072-intensity.png]

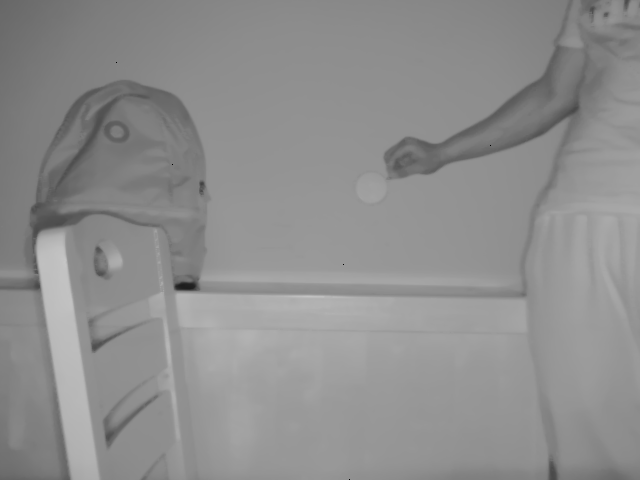

Supplement: Supplementary file 1 [file Data_Sheet_1.ZIP › The experimental data/grayscale image/tof640-20gm-22543413-0073-intensity.png]

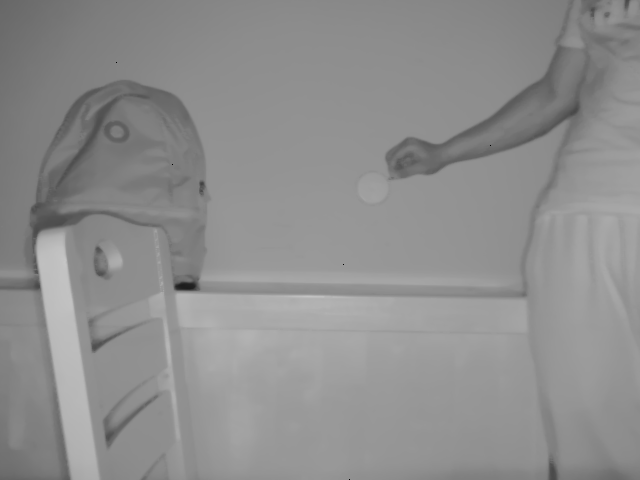

Supplement: Supplementary file 1 [file Data_Sheet_1.ZIP › The experimental data/grayscale image/tof640-20gm-22543413-0074-intensity.png]

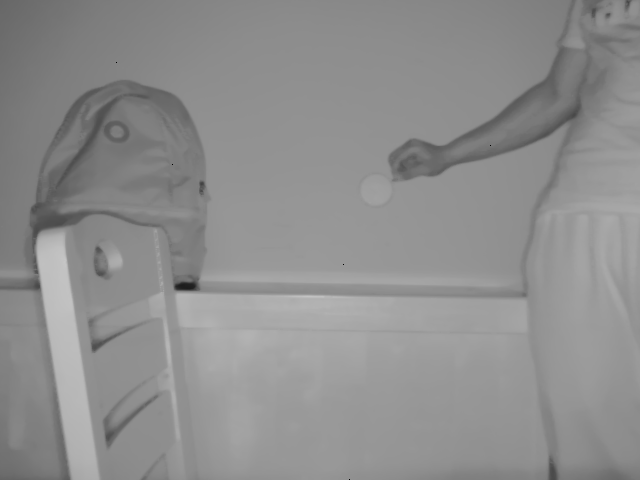

Supplement: Supplementary file 1 [file Data_Sheet_1.ZIP › The experimental data/grayscale image/tof640-20gm-22543413-0075-intensity.png]

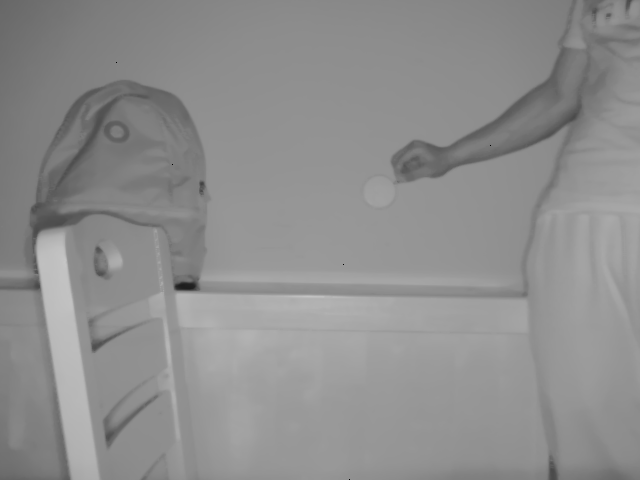

Supplement: Supplementary file 1 [file Data_Sheet_1.ZIP › The experimental data/grayscale image/tof640-20gm-22543413-0076-intensity.png]

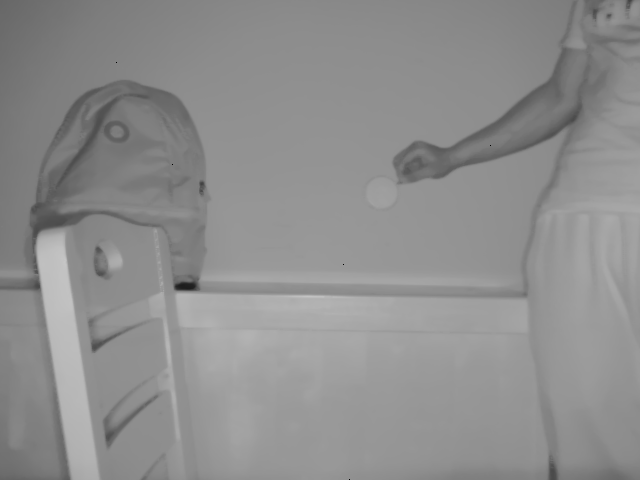

Supplement: Supplementary file 1 [file Data_Sheet_1.ZIP › The experimental data/grayscale image/tof640-20gm-22543413-0077-intensity.png]

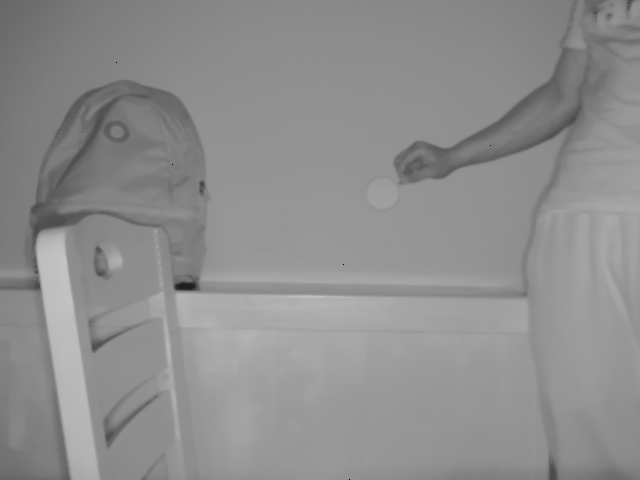

Supplement: Supplementary file 1 [file Data_Sheet_1.ZIP › The experimental data/grayscale image/tof640-20gm-22543413-0078-intensity.png]

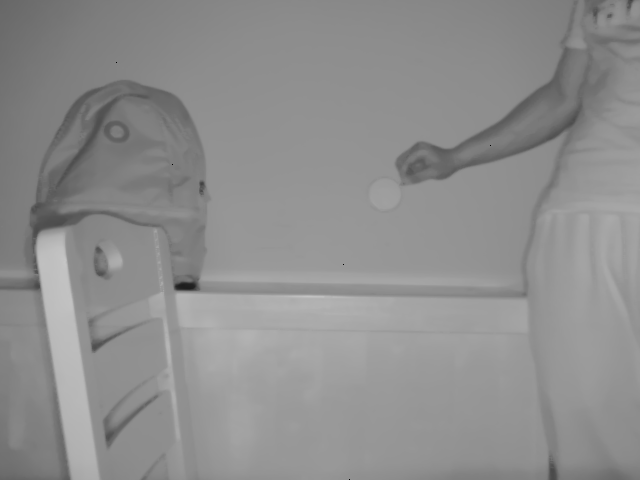

Supplement: Supplementary file 1 [file Data_Sheet_1.ZIP › The experimental data/grayscale image/tof640-20gm-22543413-0079-intensity.png]

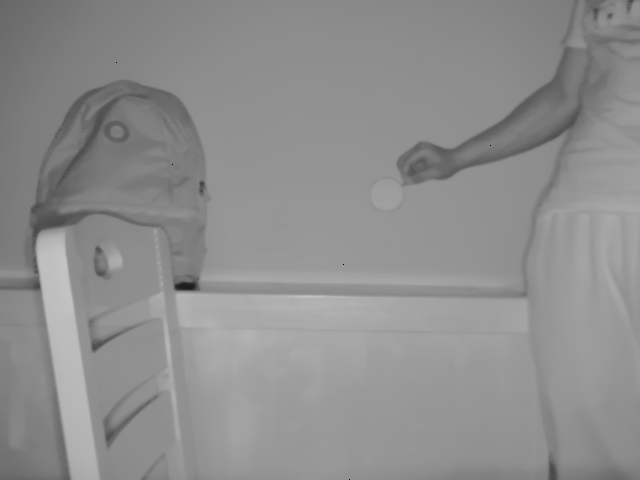

Supplement: Supplementary file 1 [file Data_Sheet_1.ZIP › The experimental data/grayscale image/tof640-20gm-22543413-0080-intensity.png]

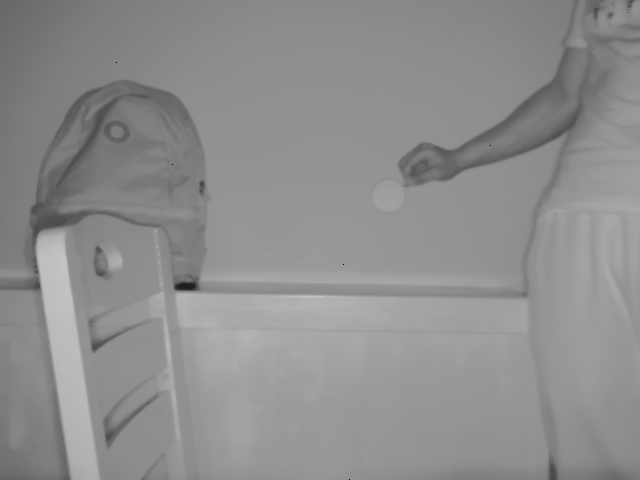

Supplement: Supplementary file 1 [file Data_Sheet_1.ZIP › The experimental data/grayscale image/tof640-20gm-22543413-0081-intensity.png]
