# Supplementary figures and images for: Introducing Depth Information Into Generative Target Tracking (part 1 of 2)
Source: Front Neurorobot. 2021 Sep 1;15:718681. doi: 10.3389/fnbot.2021.718681 (PMC8442731; doi:10.3389/fnbot.2021.718681)

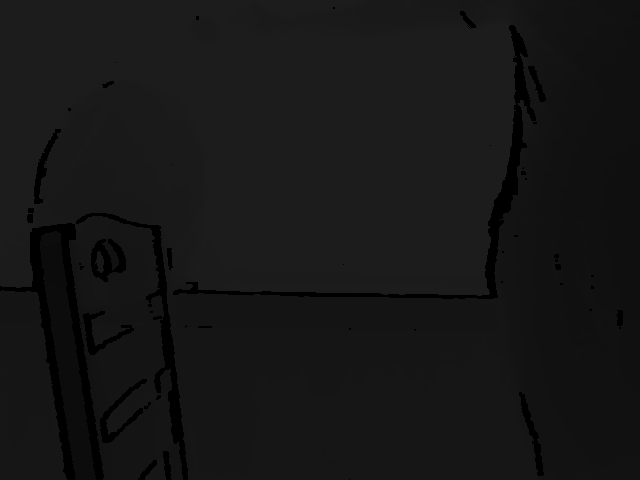

Supplement: Supplementary file 1 [file Data_Sheet_1.ZIP › The experimental data/corresponding depth image/tof640-20gm-22543413-0001-range_gray.png]

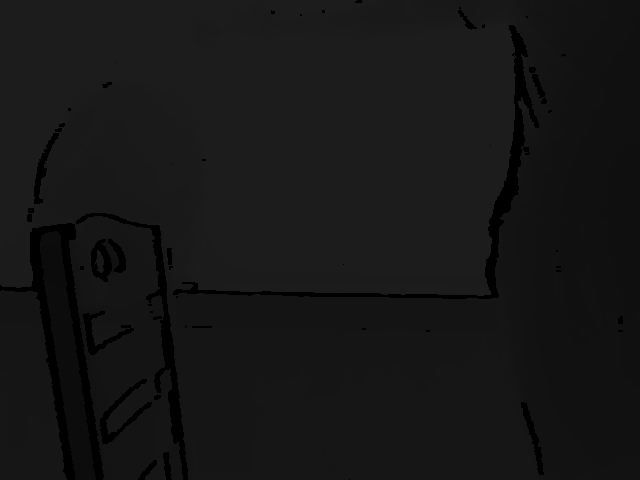

Supplement: Supplementary file 1 [file Data_Sheet_1.ZIP › The experimental data/corresponding depth image/tof640-20gm-22543413-0002-range_gray.png]

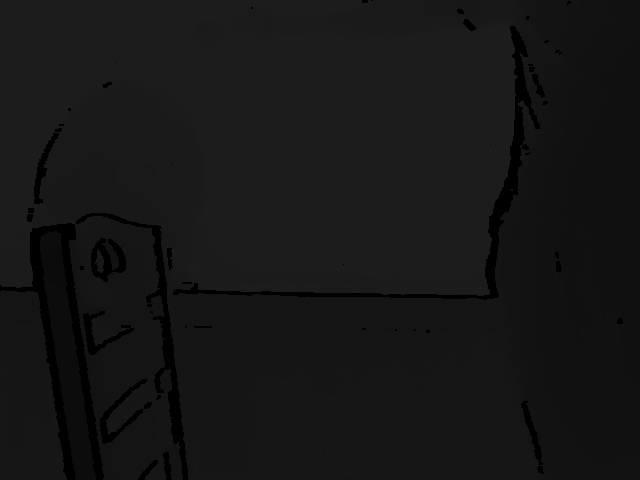

Supplement: Supplementary file 1 [file Data_Sheet_1.ZIP › The experimental data/corresponding depth image/tof640-20gm-22543413-0003-range_gray.png]

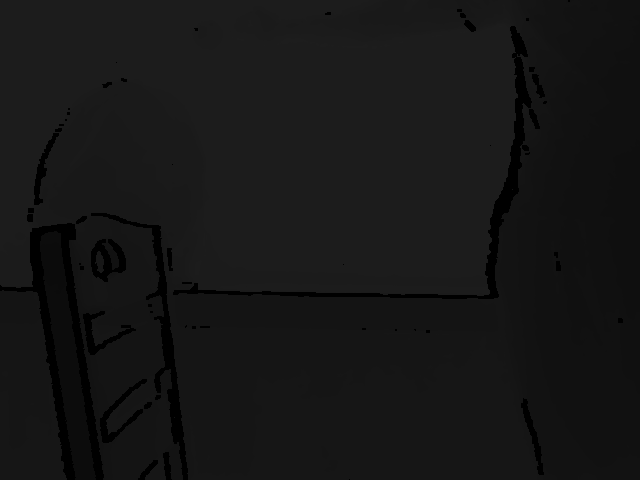

Supplement: Supplementary file 1 [file Data_Sheet_1.ZIP › The experimental data/corresponding depth image/tof640-20gm-22543413-0004-range_gray.png]

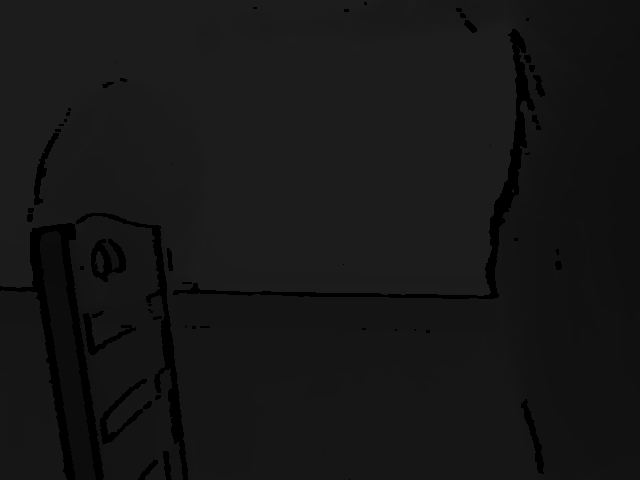

Supplement: Supplementary file 1 [file Data_Sheet_1.ZIP › The experimental data/corresponding depth image/tof640-20gm-22543413-0005-range_gray.png]

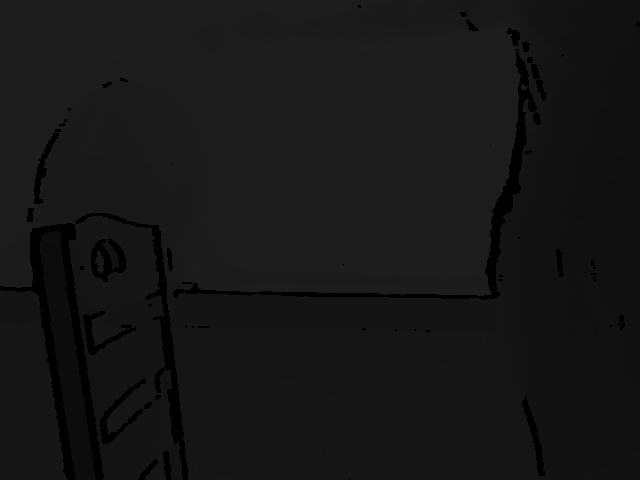

Supplement: Supplementary file 1 [file Data_Sheet_1.ZIP › The experimental data/corresponding depth image/tof640-20gm-22543413-0006-range_gray.png]

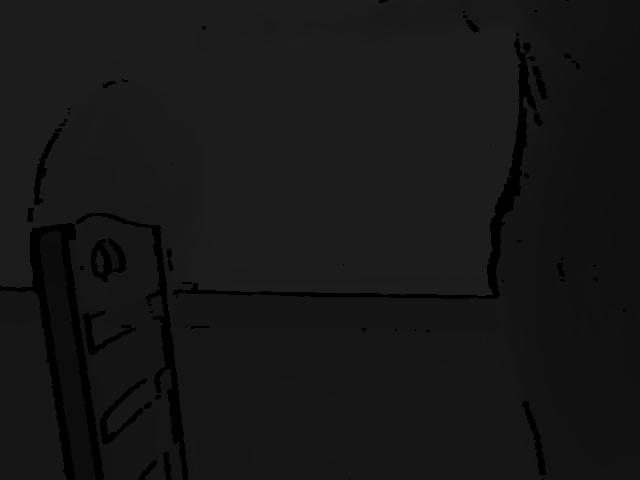

Supplement: Supplementary file 1 [file Data_Sheet_1.ZIP › The experimental data/corresponding depth image/tof640-20gm-22543413-0007-range_gray.png]

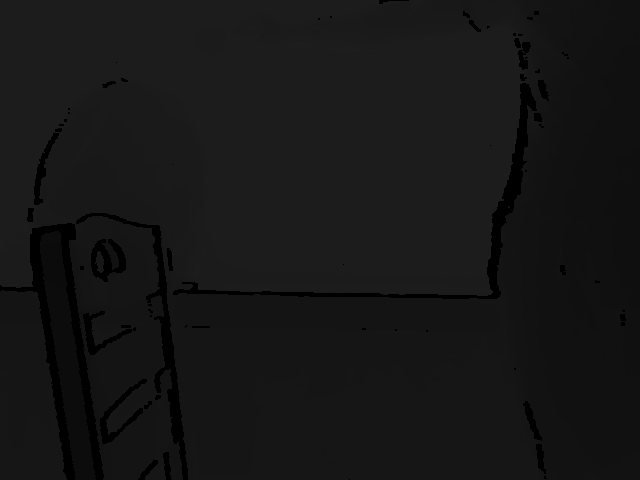

Supplement: Supplementary file 1 [file Data_Sheet_1.ZIP › The experimental data/corresponding depth image/tof640-20gm-22543413-0008-range_gray.png]

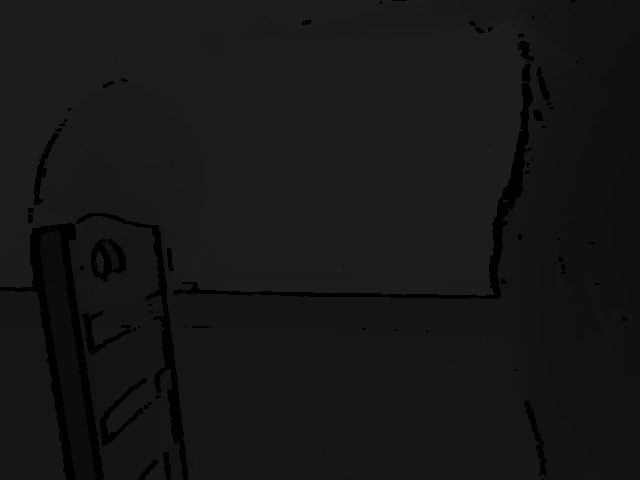

Supplement: Supplementary file 1 [file Data_Sheet_1.ZIP › The experimental data/corresponding depth image/tof640-20gm-22543413-0009-range_gray.png]

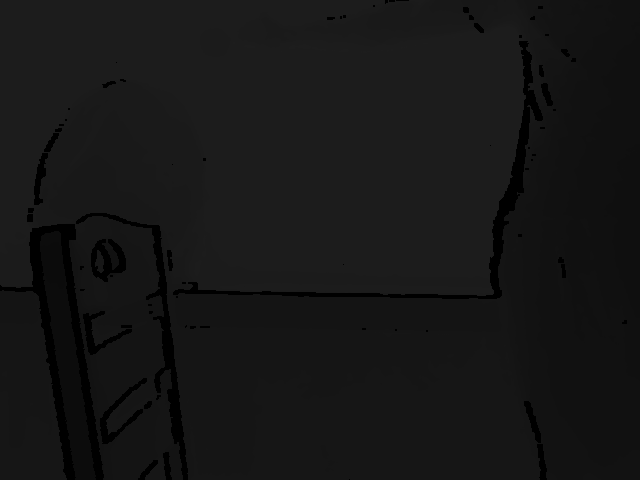

Supplement: Supplementary file 1 [file Data_Sheet_1.ZIP › The experimental data/corresponding depth image/tof640-20gm-22543413-0010-range_gray.png]

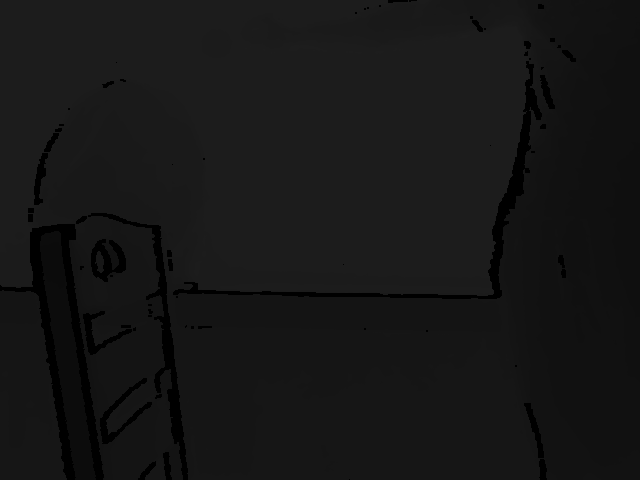

Supplement: Supplementary file 1 [file Data_Sheet_1.ZIP › The experimental data/corresponding depth image/tof640-20gm-22543413-0011-range_gray.png]

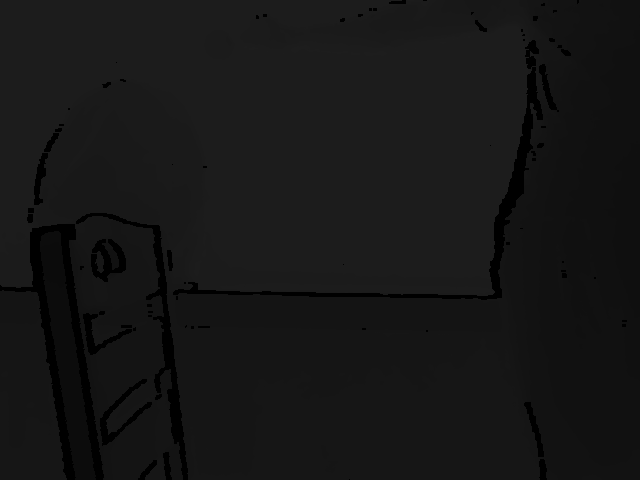

Supplement: Supplementary file 1 [file Data_Sheet_1.ZIP › The experimental data/corresponding depth image/tof640-20gm-22543413-0012-range_gray.png]

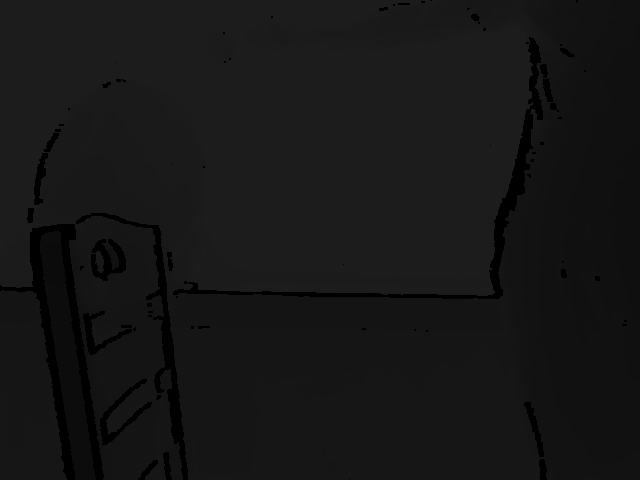

Supplement: Supplementary file 1 [file Data_Sheet_1.ZIP › The experimental data/corresponding depth image/tof640-20gm-22543413-0013-range_gray.png]

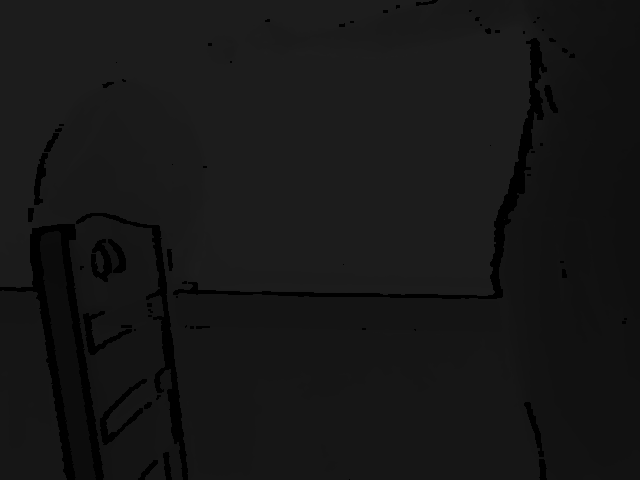

Supplement: Supplementary file 1 [file Data_Sheet_1.ZIP › The experimental data/corresponding depth image/tof640-20gm-22543413-0014-range_gray.png]

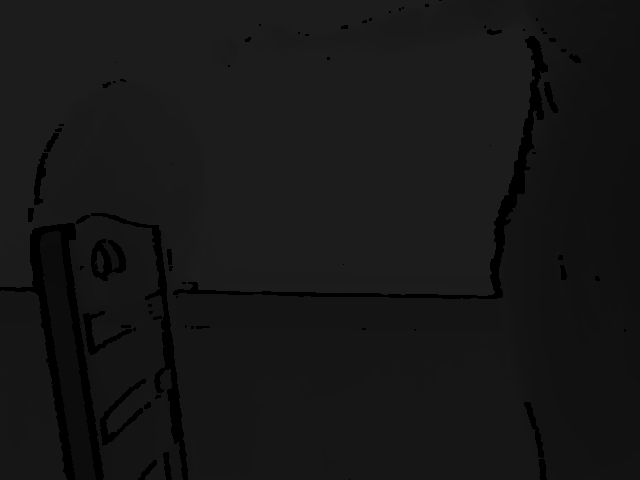

Supplement: Supplementary file 1 [file Data_Sheet_1.ZIP › The experimental data/corresponding depth image/tof640-20gm-22543413-0015-range_gray.png]

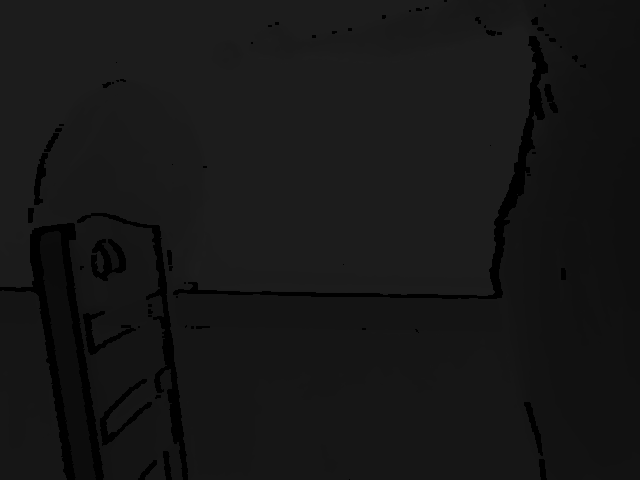

Supplement: Supplementary file 1 [file Data_Sheet_1.ZIP › The experimental data/corresponding depth image/tof640-20gm-22543413-0016-range_gray.png]

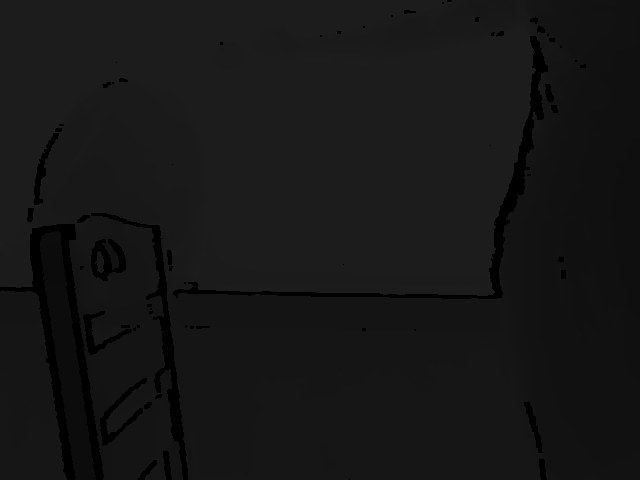

Supplement: Supplementary file 1 [file Data_Sheet_1.ZIP › The experimental data/corresponding depth image/tof640-20gm-22543413-0017-range_gray.png]

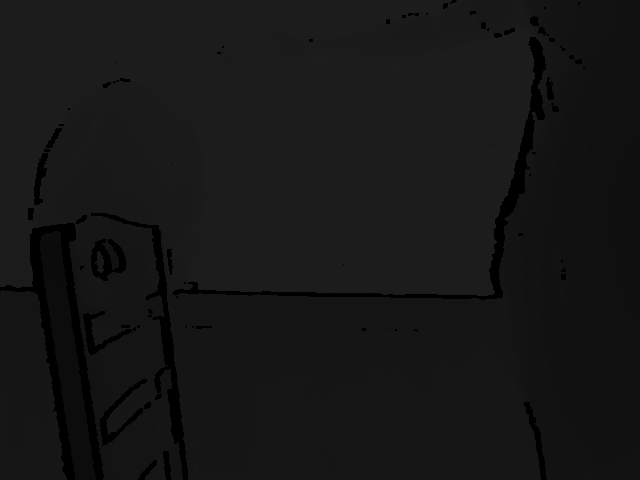

Supplement: Supplementary file 1 [file Data_Sheet_1.ZIP › The experimental data/corresponding depth image/tof640-20gm-22543413-0018-range_gray.png]

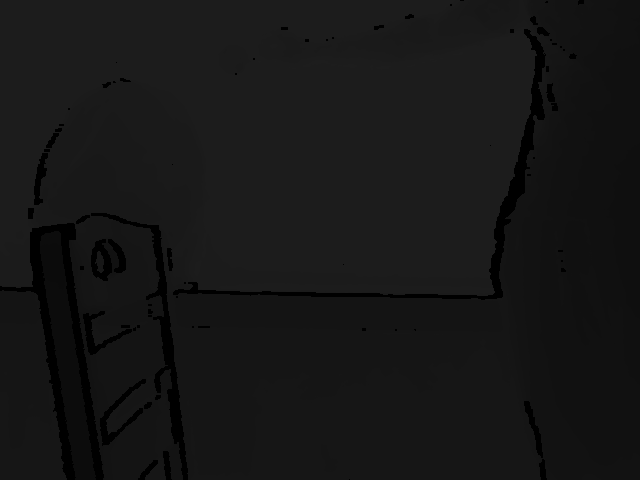

Supplement: Supplementary file 1 [file Data_Sheet_1.ZIP › The experimental data/corresponding depth image/tof640-20gm-22543413-0019-range_gray.png]

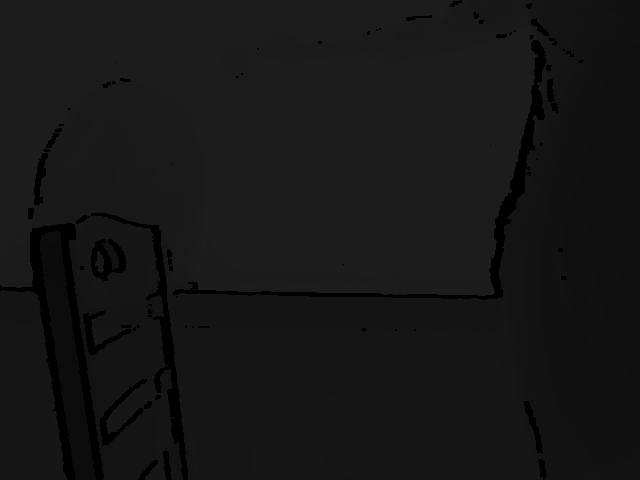

Supplement: Supplementary file 1 [file Data_Sheet_1.ZIP › The experimental data/corresponding depth image/tof640-20gm-22543413-0020-range_gray.png]

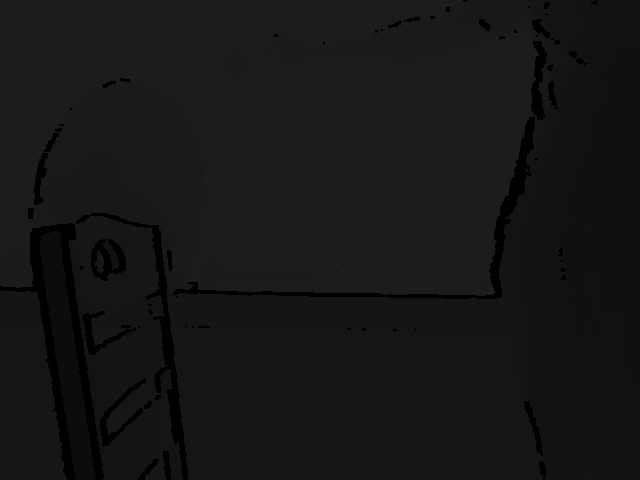

Supplement: Supplementary file 1 [file Data_Sheet_1.ZIP › The experimental data/corresponding depth image/tof640-20gm-22543413-0021-range_gray.png]

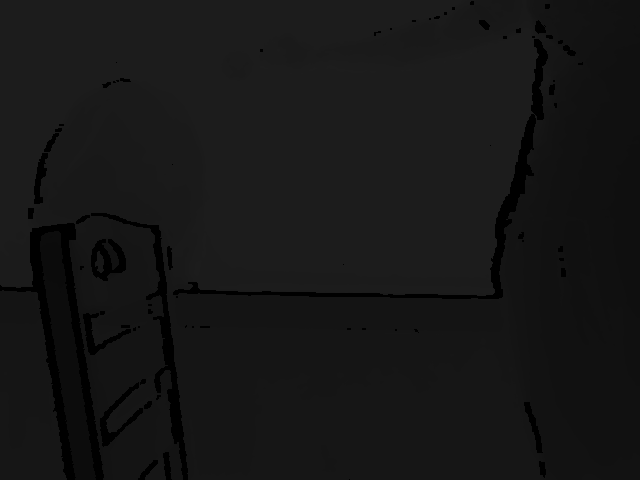

Supplement: Supplementary file 1 [file Data_Sheet_1.ZIP › The experimental data/corresponding depth image/tof640-20gm-22543413-0022-range_gray.png]

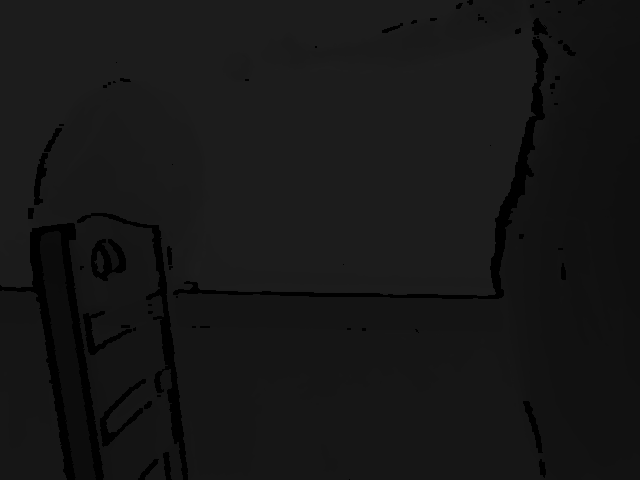

Supplement: Supplementary file 1 [file Data_Sheet_1.ZIP › The experimental data/corresponding depth image/tof640-20gm-22543413-0023-range_gray.png]

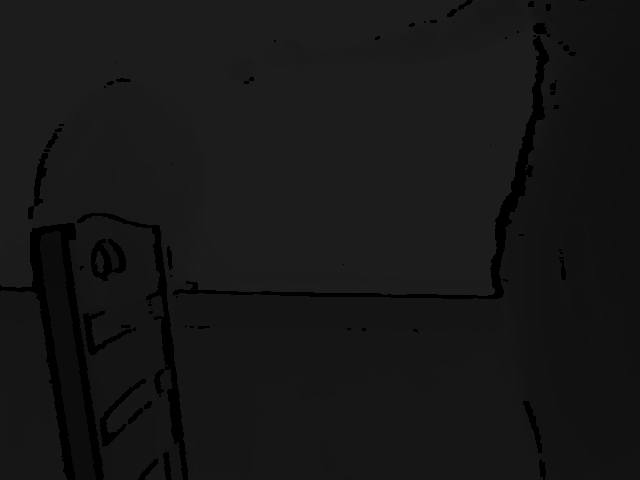

Supplement: Supplementary file 1 [file Data_Sheet_1.ZIP › The experimental data/corresponding depth image/tof640-20gm-22543413-0024-range_gray.png]

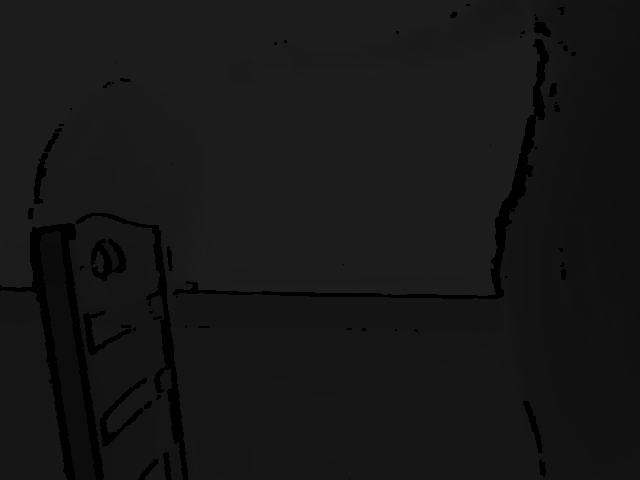

Supplement: Supplementary file 1 [file Data_Sheet_1.ZIP › The experimental data/corresponding depth image/tof640-20gm-22543413-0025-range_gray.png]

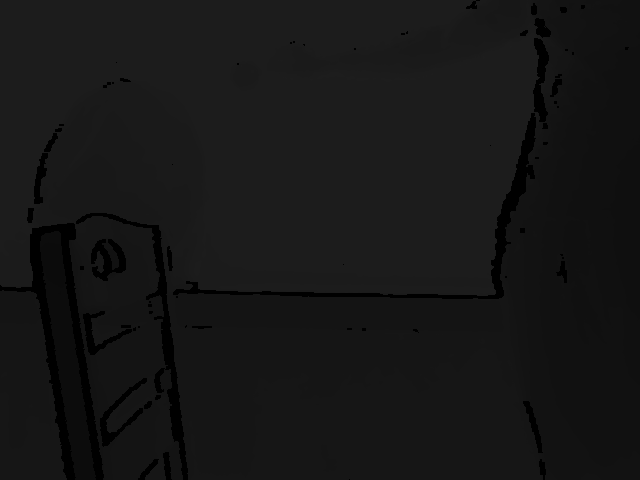

Supplement: Supplementary file 1 [file Data_Sheet_1.ZIP › The experimental data/corresponding depth image/tof640-20gm-22543413-0026-range_gray.png]

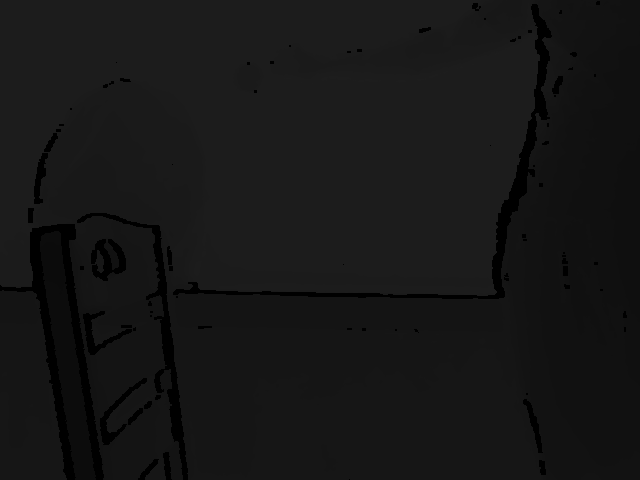

Supplement: Supplementary file 1 [file Data_Sheet_1.ZIP › The experimental data/corresponding depth image/tof640-20gm-22543413-0027-range_gray.png]

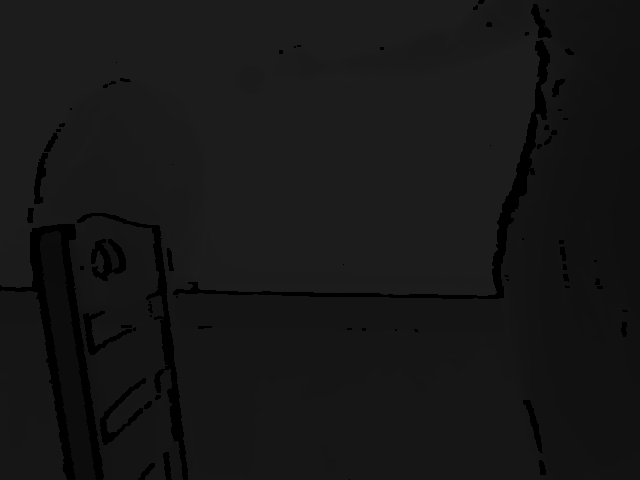

Supplement: Supplementary file 1 [file Data_Sheet_1.ZIP › The experimental data/corresponding depth image/tof640-20gm-22543413-0028-range_gray.png]

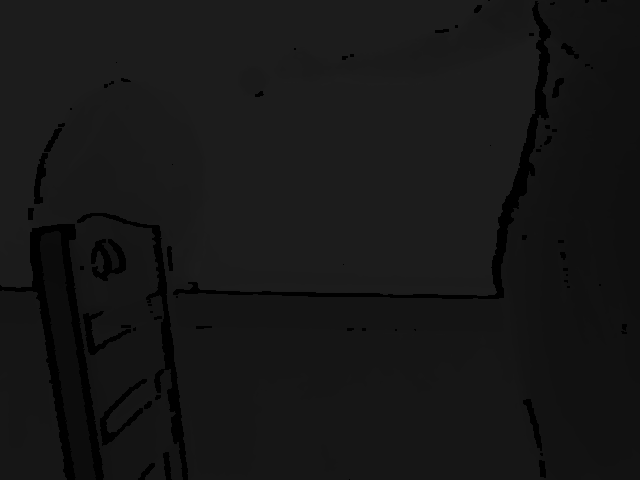

Supplement: Supplementary file 1 [file Data_Sheet_1.ZIP › The experimental data/corresponding depth image/tof640-20gm-22543413-0029-range_gray.png]

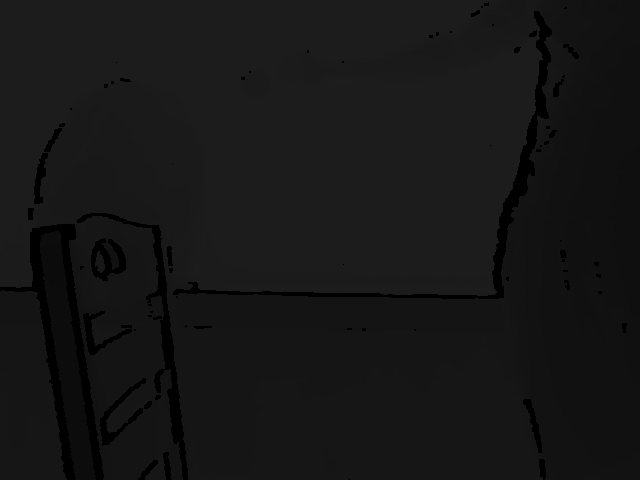

Supplement: Supplementary file 1 [file Data_Sheet_1.ZIP › The experimental data/corresponding depth image/tof640-20gm-22543413-0030-range_gray.png]

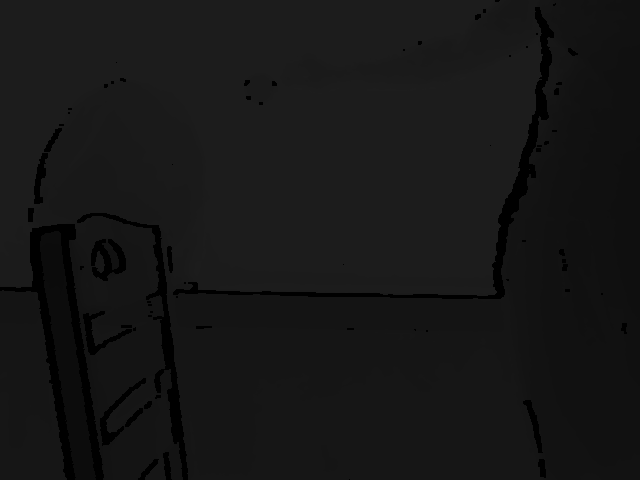

Supplement: Supplementary file 1 [file Data_Sheet_1.ZIP › The experimental data/corresponding depth image/tof640-20gm-22543413-0031-range_gray.png]

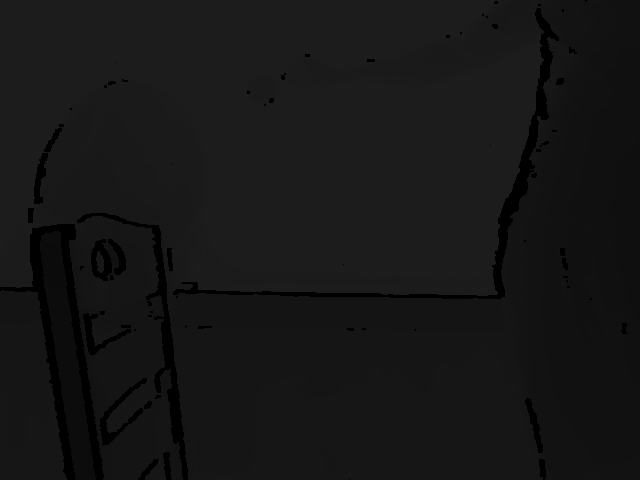

Supplement: Supplementary file 1 [file Data_Sheet_1.ZIP › The experimental data/corresponding depth image/tof640-20gm-22543413-0032-range_gray.png]

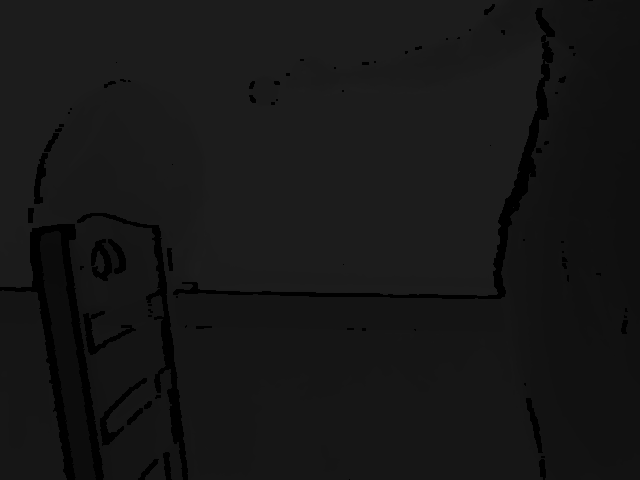

Supplement: Supplementary file 1 [file Data_Sheet_1.ZIP › The experimental data/corresponding depth image/tof640-20gm-22543413-0033-range_gray.png]

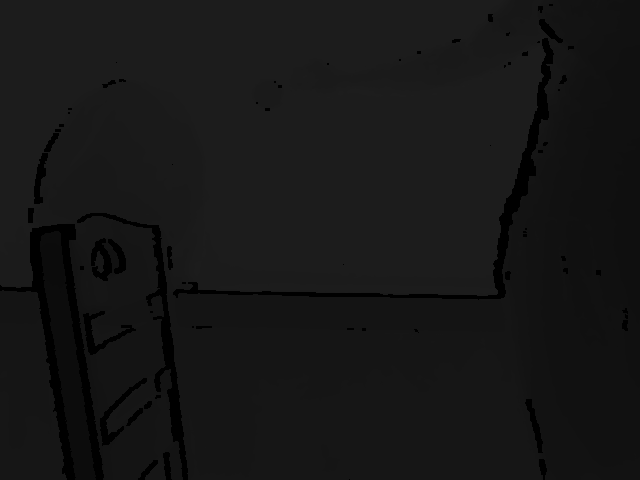

Supplement: Supplementary file 1 [file Data_Sheet_1.ZIP › The experimental data/corresponding depth image/tof640-20gm-22543413-0034-range_gray.png]

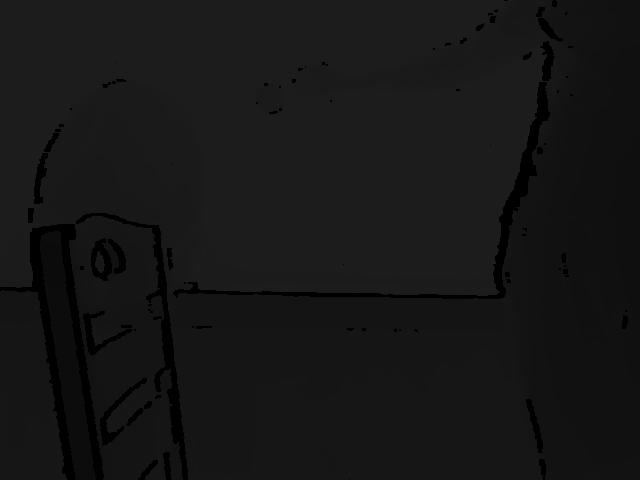

Supplement: Supplementary file 1 [file Data_Sheet_1.ZIP › The experimental data/corresponding depth image/tof640-20gm-22543413-0035-range_gray.png]

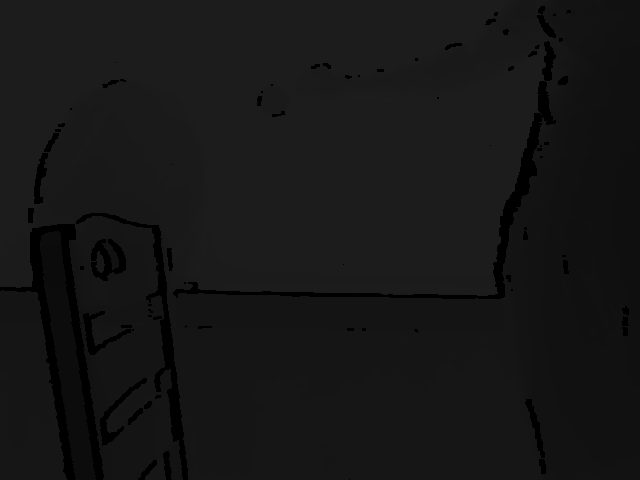

Supplement: Supplementary file 1 [file Data_Sheet_1.ZIP › The experimental data/corresponding depth image/tof640-20gm-22543413-0036-range_gray.png]

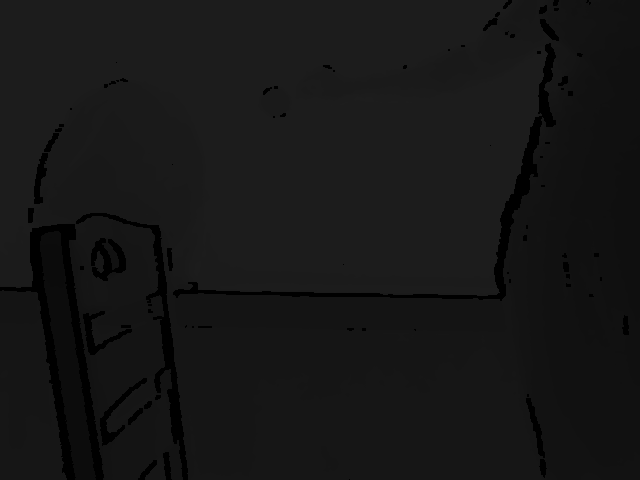

Supplement: Supplementary file 1 [file Data_Sheet_1.ZIP › The experimental data/corresponding depth image/tof640-20gm-22543413-0037-range_gray.png]

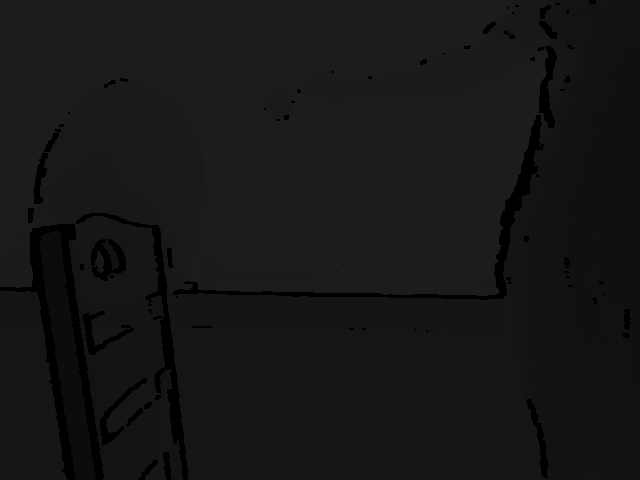

Supplement: Supplementary file 1 [file Data_Sheet_1.ZIP › The experimental data/corresponding depth image/tof640-20gm-22543413-0038-range_gray.png]

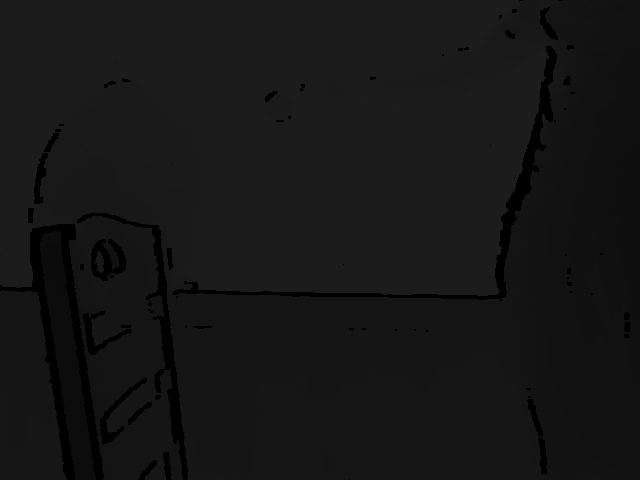

Supplement: Supplementary file 1 [file Data_Sheet_1.ZIP › The experimental data/corresponding depth image/tof640-20gm-22543413-0039-range_gray.png]

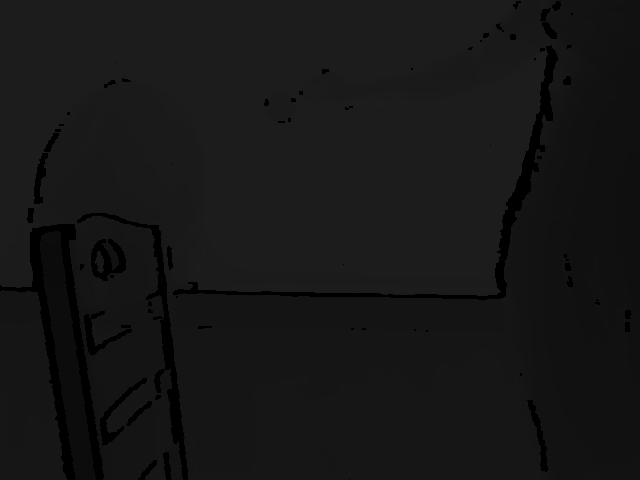

Supplement: Supplementary file 1 [file Data_Sheet_1.ZIP › The experimental data/corresponding depth image/tof640-20gm-22543413-0040-range_gray.png]

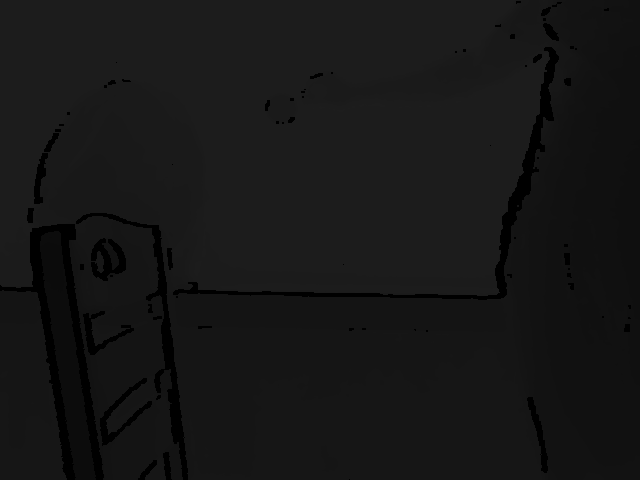

Supplement: Supplementary file 1 [file Data_Sheet_1.ZIP › The experimental data/corresponding depth image/tof640-20gm-22543413-0041-range_gray.png]

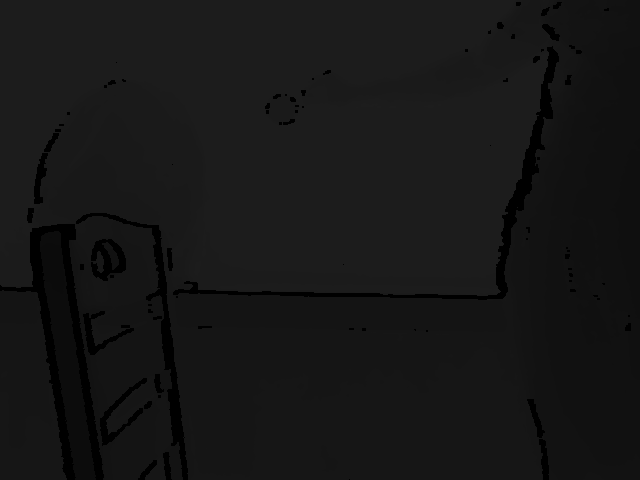

Supplement: Supplementary file 1 [file Data_Sheet_1.ZIP › The experimental data/corresponding depth image/tof640-20gm-22543413-0042-range_gray.png]

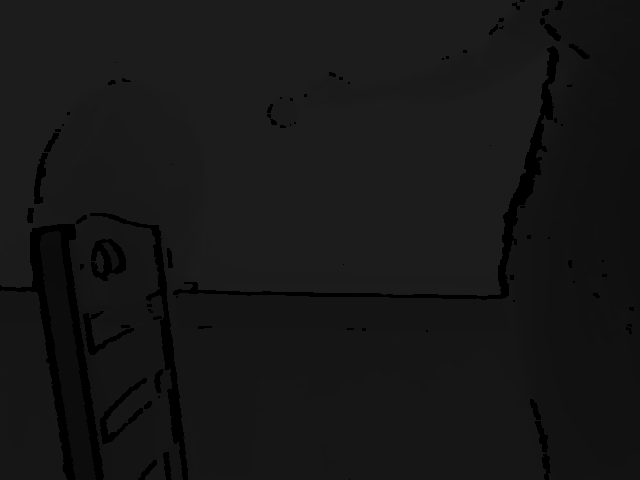

Supplement: Supplementary file 1 [file Data_Sheet_1.ZIP › The experimental data/corresponding depth image/tof640-20gm-22543413-0043-range_gray.png]

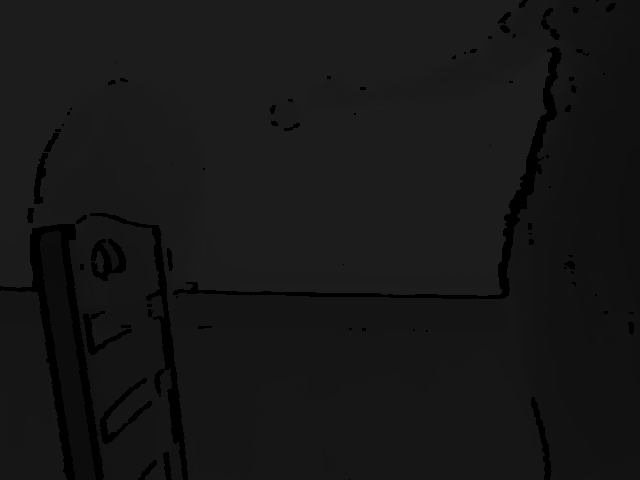

Supplement: Supplementary file 1 [file Data_Sheet_1.ZIP › The experimental data/corresponding depth image/tof640-20gm-22543413-0044-range_gray.png]

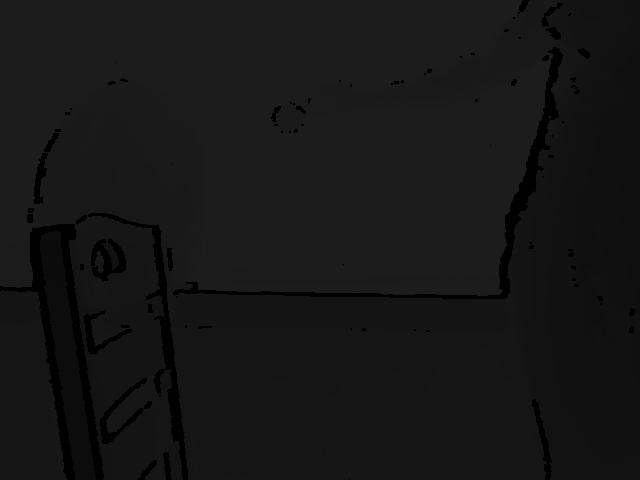

Supplement: Supplementary file 1 [file Data_Sheet_1.ZIP › The experimental data/corresponding depth image/tof640-20gm-22543413-0045-range_gray.png]

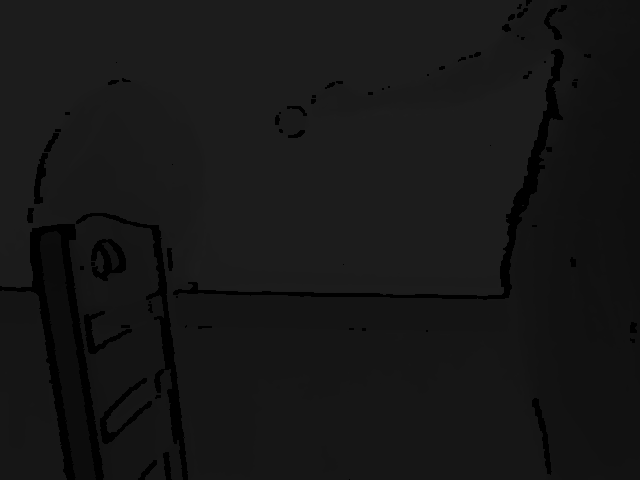

Supplement: Supplementary file 1 [file Data_Sheet_1.ZIP › The experimental data/corresponding depth image/tof640-20gm-22543413-0046-range_gray.png]

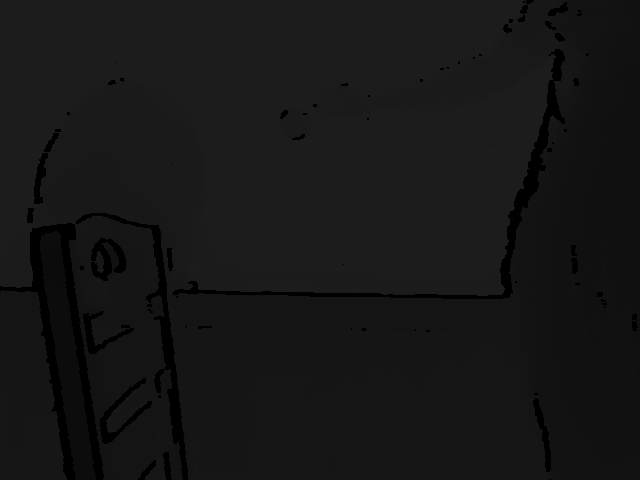

Supplement: Supplementary file 1 [file Data_Sheet_1.ZIP › The experimental data/corresponding depth image/tof640-20gm-22543413-0047-range_gray.png]

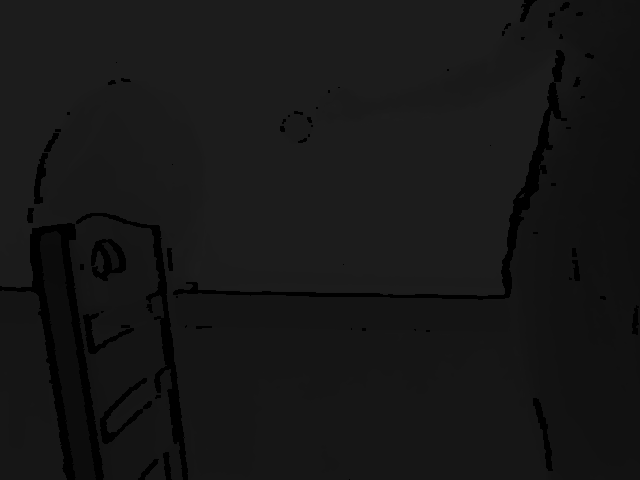

Supplement: Supplementary file 1 [file Data_Sheet_1.ZIP › The experimental data/corresponding depth image/tof640-20gm-22543413-0048-range_gray.png]

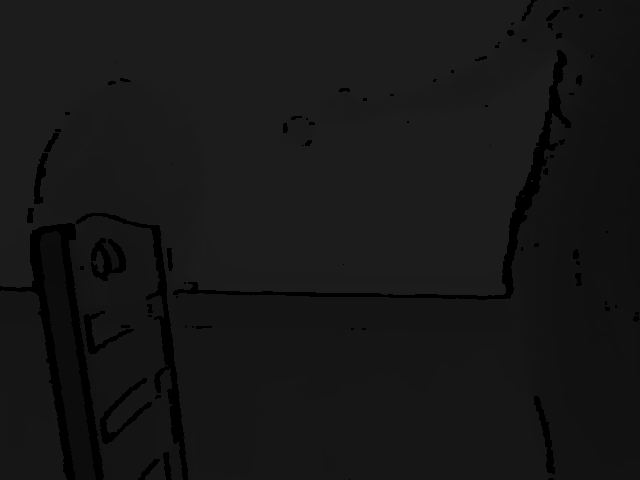

Supplement: Supplementary file 1 [file Data_Sheet_1.ZIP › The experimental data/corresponding depth image/tof640-20gm-22543413-0049-range_gray.png]

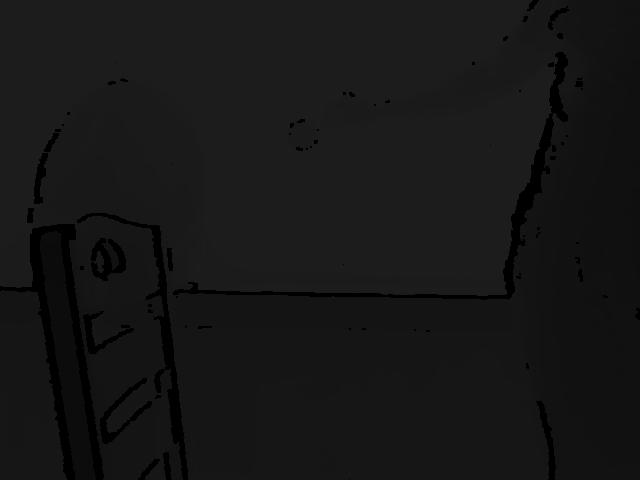

Supplement: Supplementary file 1 [file Data_Sheet_1.ZIP › The experimental data/corresponding depth image/tof640-20gm-22543413-0050-range_gray.png]

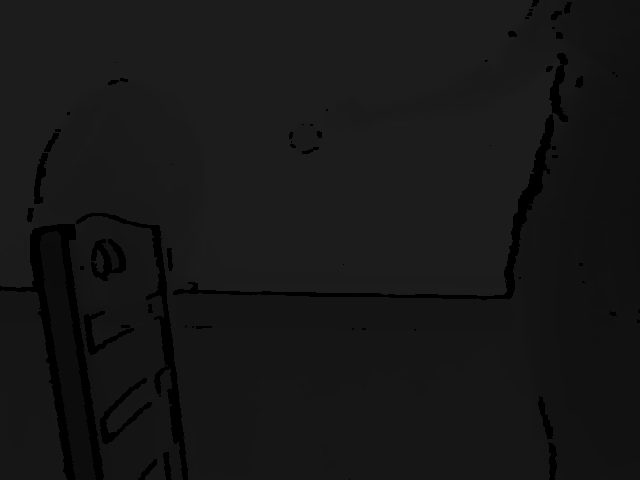

Supplement: Supplementary file 1 [file Data_Sheet_1.ZIP › The experimental data/corresponding depth image/tof640-20gm-22543413-0051-range_gray.png]

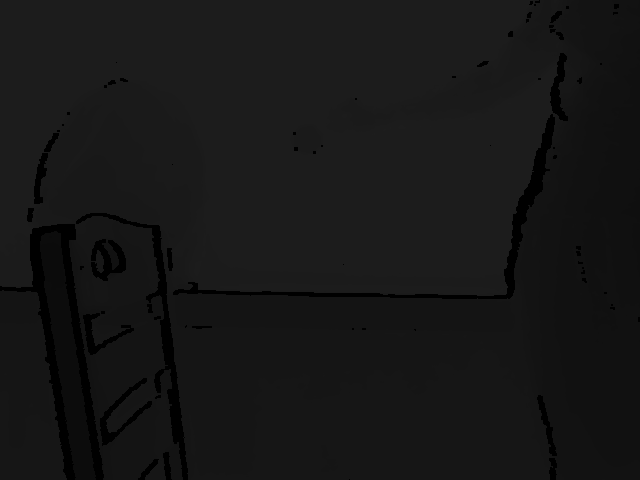

Supplement: Supplementary file 1 [file Data_Sheet_1.ZIP › The experimental data/corresponding depth image/tof640-20gm-22543413-0052-range_gray.png]

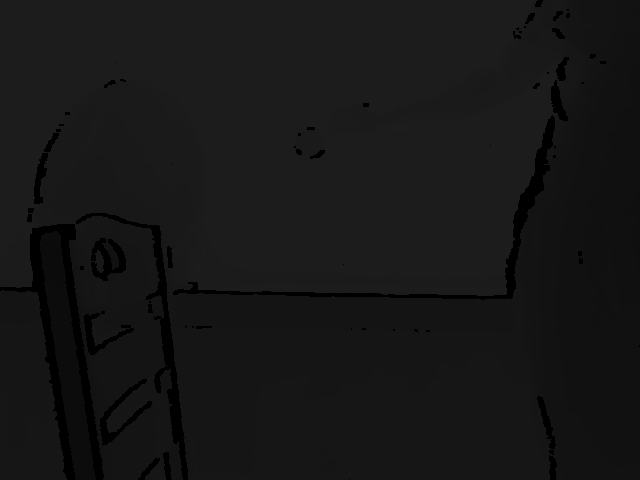

Supplement: Supplementary file 1 [file Data_Sheet_1.ZIP › The experimental data/corresponding depth image/tof640-20gm-22543413-0053-range_gray.png]

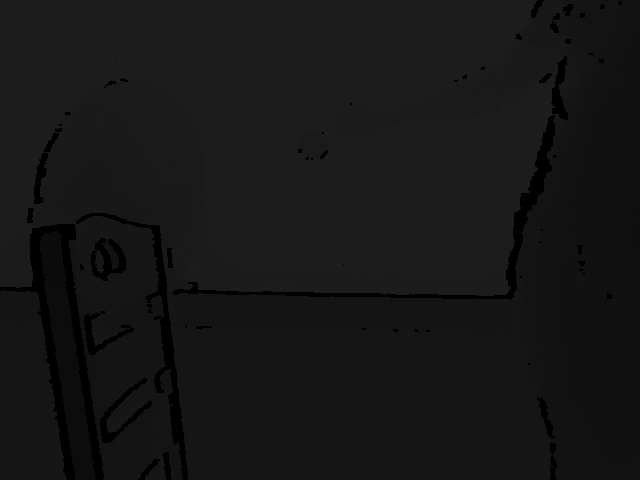

Supplement: Supplementary file 1 [file Data_Sheet_1.ZIP › The experimental data/corresponding depth image/tof640-20gm-22543413-0054-range_gray.png]

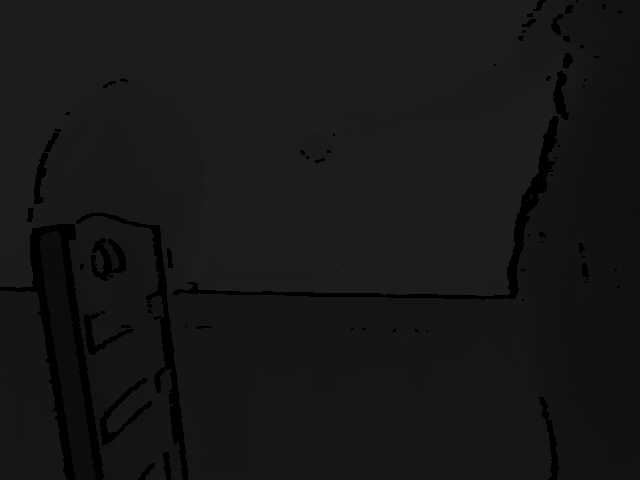

Supplement: Supplementary file 1 [file Data_Sheet_1.ZIP › The experimental data/corresponding depth image/tof640-20gm-22543413-0055-range_gray.png]

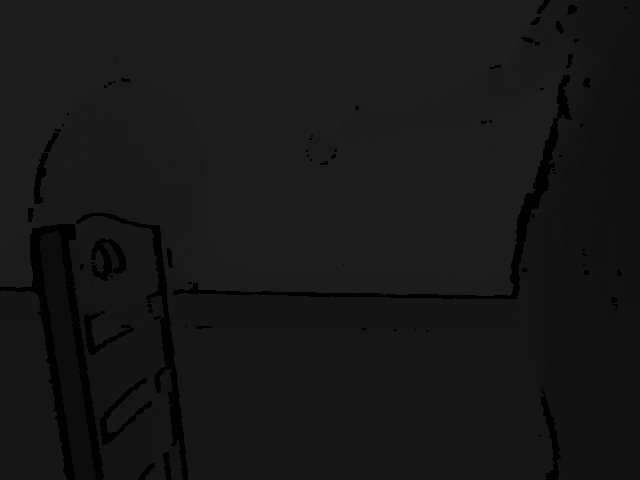

Supplement: Supplementary file 1 [file Data_Sheet_1.ZIP › The experimental data/corresponding depth image/tof640-20gm-22543413-0056-range_gray.png]

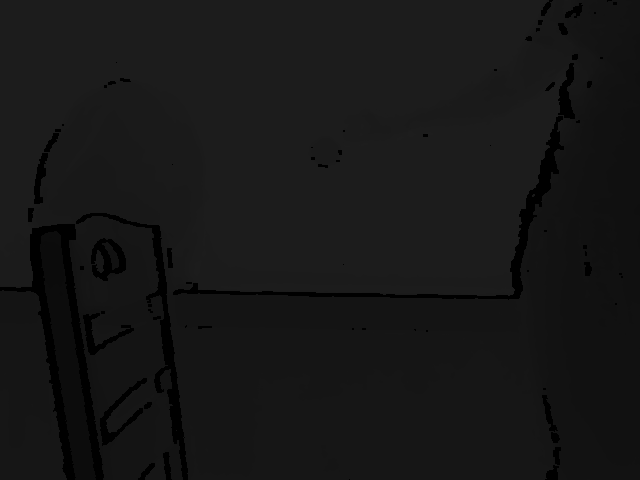

Supplement: Supplementary file 1 [file Data_Sheet_1.ZIP › The experimental data/corresponding depth image/tof640-20gm-22543413-0057-range_gray.png]

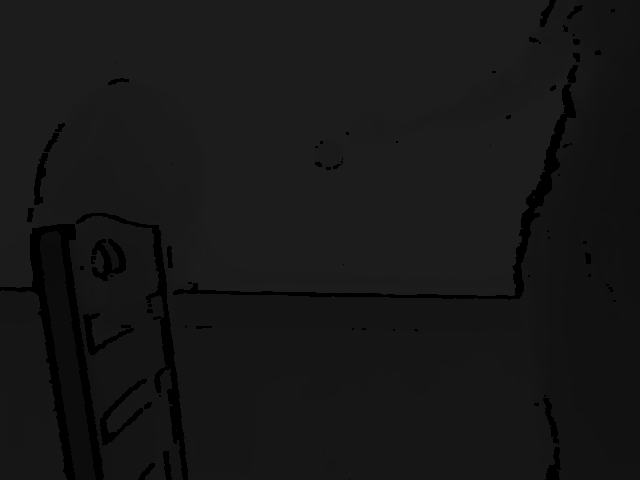

Supplement: Supplementary file 1 [file Data_Sheet_1.ZIP › The experimental data/corresponding depth image/tof640-20gm-22543413-0058-range_gray.png]

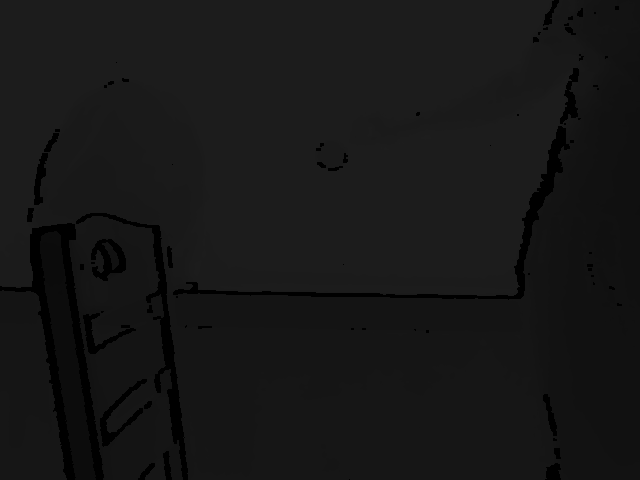

Supplement: Supplementary file 1 [file Data_Sheet_1.ZIP › The experimental data/corresponding depth image/tof640-20gm-22543413-0059-range_gray.png]

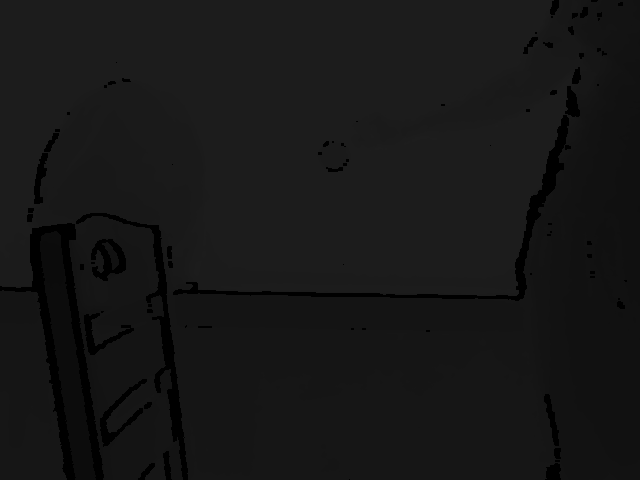

Supplement: Supplementary file 1 [file Data_Sheet_1.ZIP › The experimental data/corresponding depth image/tof640-20gm-22543413-0060-range_gray.png]

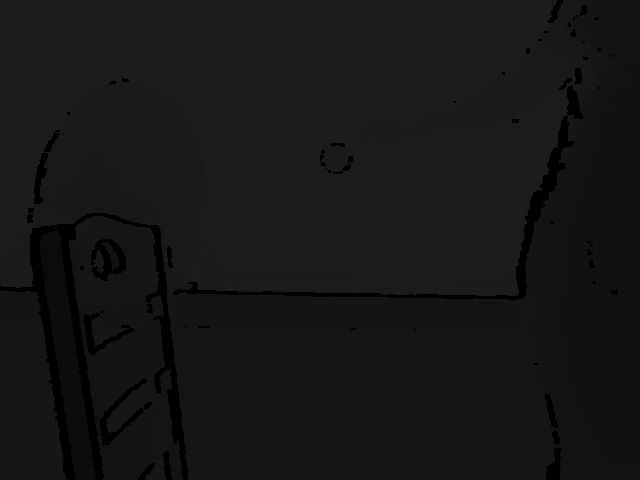

Supplement: Supplementary file 1 [file Data_Sheet_1.ZIP › The experimental data/corresponding depth image/tof640-20gm-22543413-0061-range_gray.png]

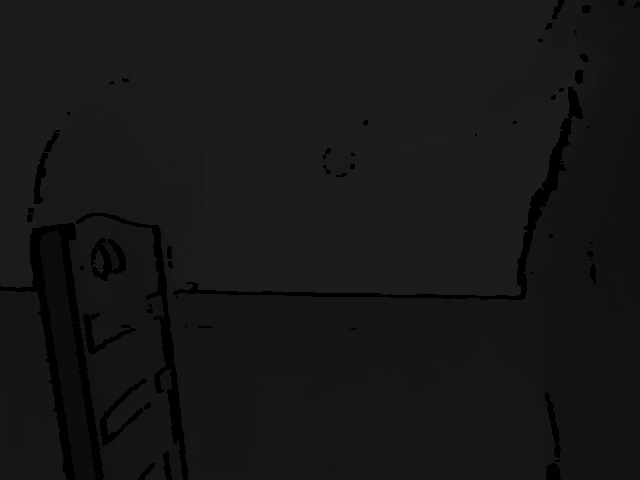

Supplement: Supplementary file 1 [file Data_Sheet_1.ZIP › The experimental data/corresponding depth image/tof640-20gm-22543413-0062-range_gray.png]

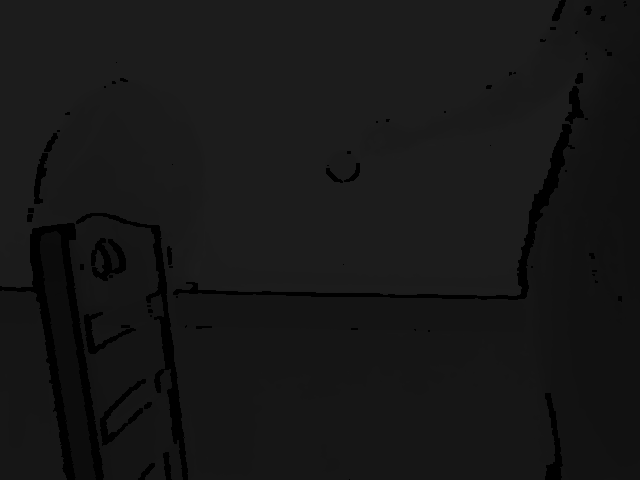

Supplement: Supplementary file 1 [file Data_Sheet_1.ZIP › The experimental data/corresponding depth image/tof640-20gm-22543413-0063-range_gray.png]

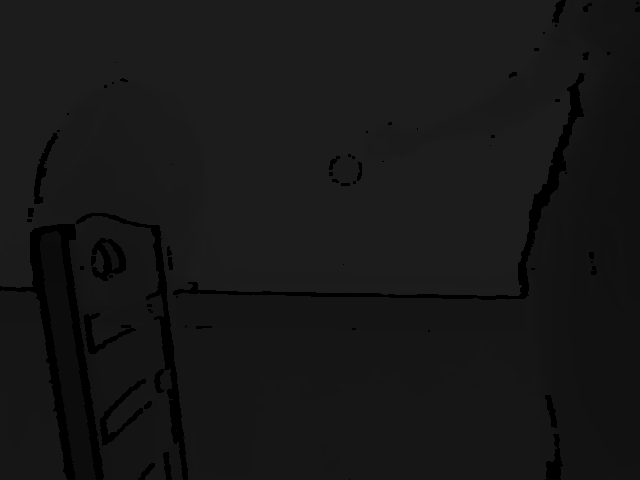

Supplement: Supplementary file 1 [file Data_Sheet_1.ZIP › The experimental data/corresponding depth image/tof640-20gm-22543413-0064-range_gray.png]

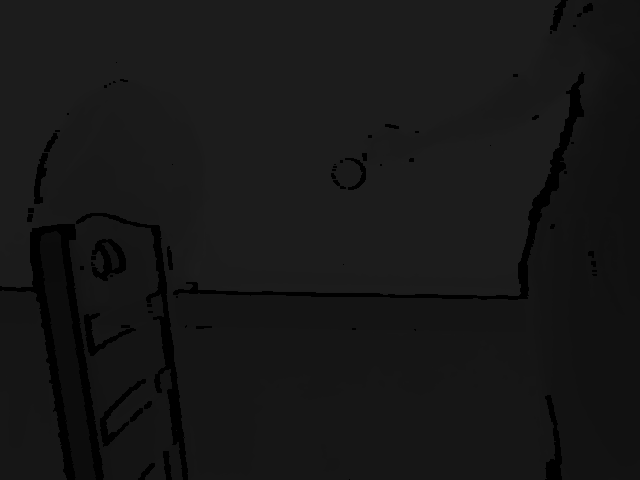

Supplement: Supplementary file 1 [file Data_Sheet_1.ZIP › The experimental data/corresponding depth image/tof640-20gm-22543413-0065-range_gray.png]

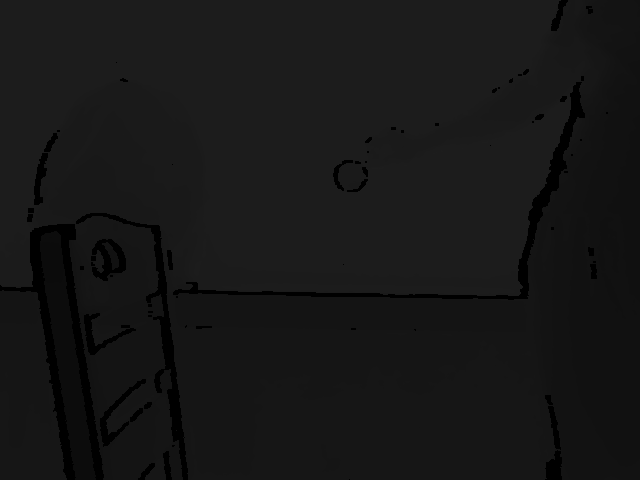

Supplement: Supplementary file 1 [file Data_Sheet_1.ZIP › The experimental data/corresponding depth image/tof640-20gm-22543413-0066-range_gray.png]

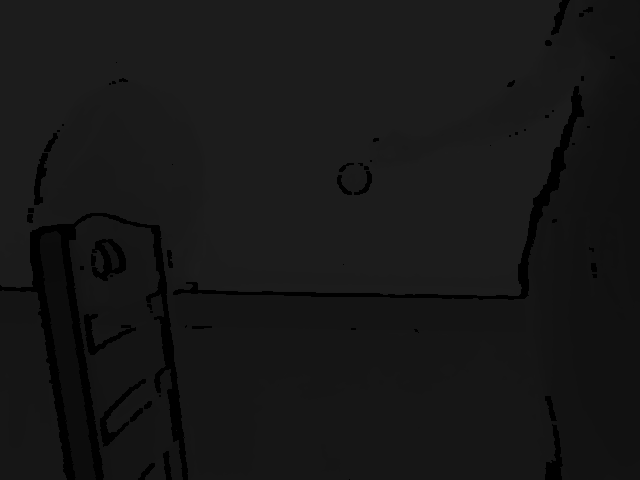

Supplement: Supplementary file 1 [file Data_Sheet_1.ZIP › The experimental data/corresponding depth image/tof640-20gm-22543413-0067-range_gray.png]

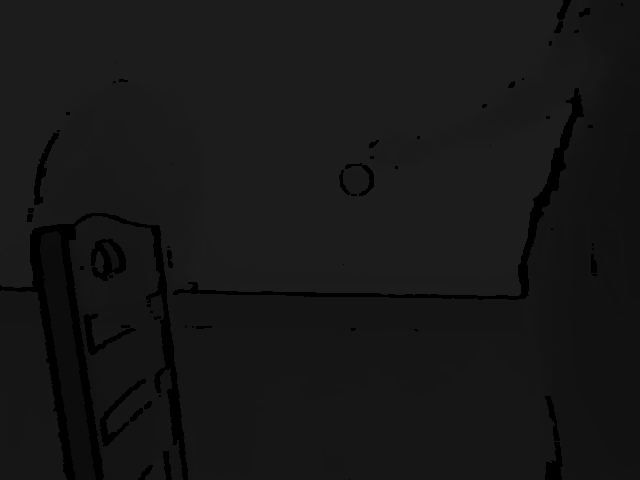

Supplement: Supplementary file 1 [file Data_Sheet_1.ZIP › The experimental data/corresponding depth image/tof640-20gm-22543413-0068-range_gray.png]

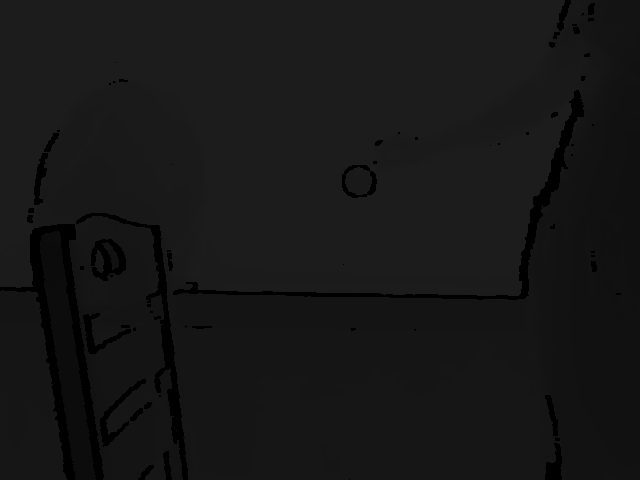

Supplement: Supplementary file 1 [file Data_Sheet_1.ZIP › The experimental data/corresponding depth image/tof640-20gm-22543413-0069-range_gray.png]

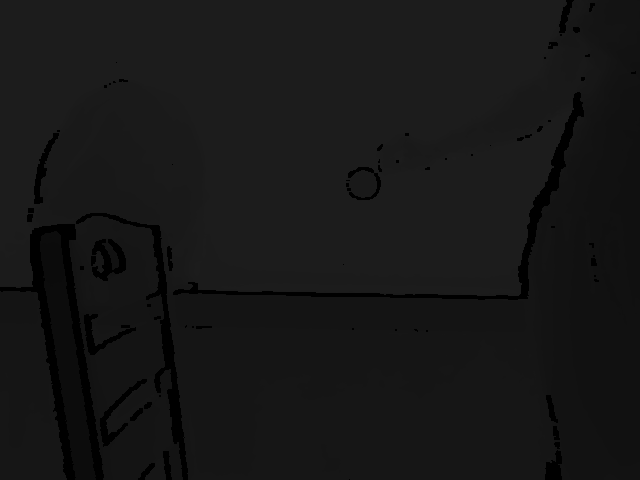

Supplement: Supplementary file 1 [file Data_Sheet_1.ZIP › The experimental data/corresponding depth image/tof640-20gm-22543413-0070-range_gray.png]

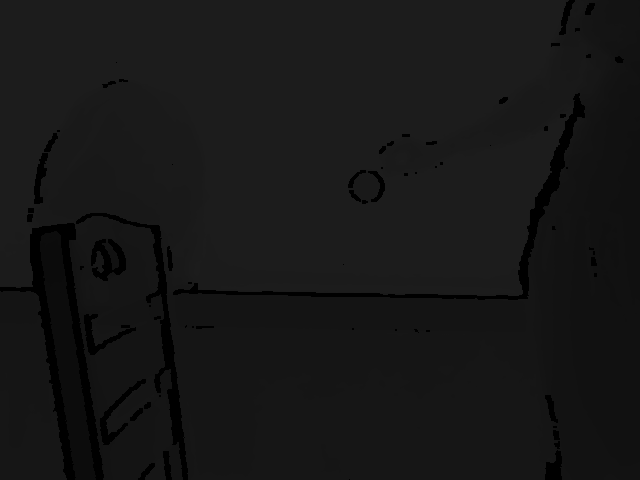

Supplement: Supplementary file 1 [file Data_Sheet_1.ZIP › The experimental data/corresponding depth image/tof640-20gm-22543413-0071-range_gray.png]

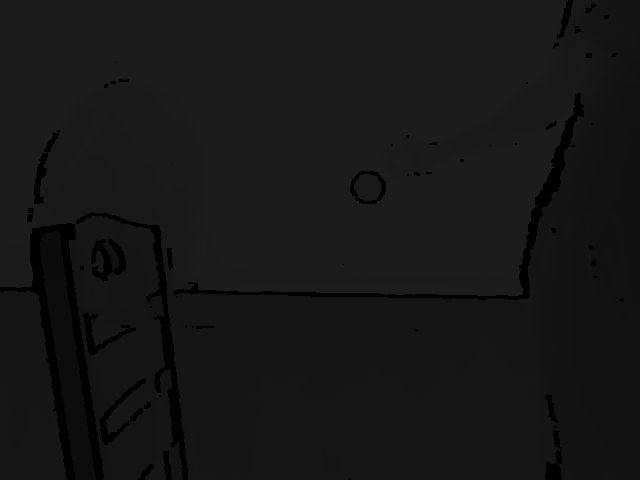

Supplement: Supplementary file 1 [file Data_Sheet_1.ZIP › The experimental data/corresponding depth image/tof640-20gm-22543413-0072-range_gray.png]

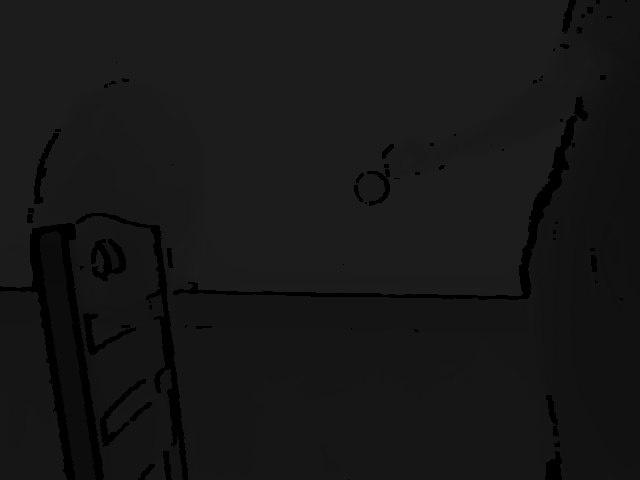

Supplement: Supplementary file 1 [file Data_Sheet_1.ZIP › The experimental data/corresponding depth image/tof640-20gm-22543413-0073-range_gray.png]

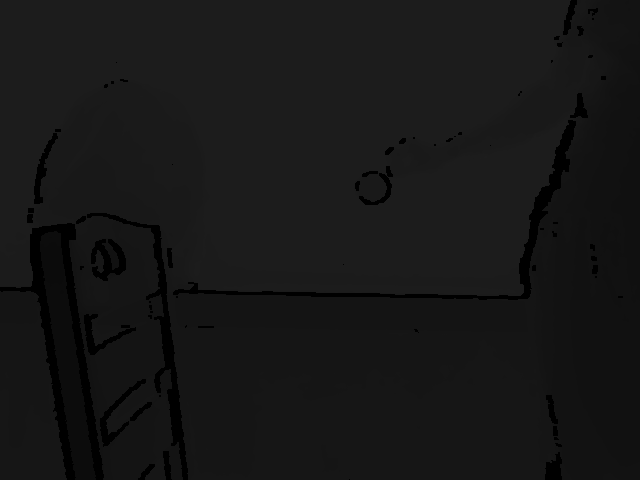

Supplement: Supplementary file 1 [file Data_Sheet_1.ZIP › The experimental data/corresponding depth image/tof640-20gm-22543413-0074-range_gray.png]

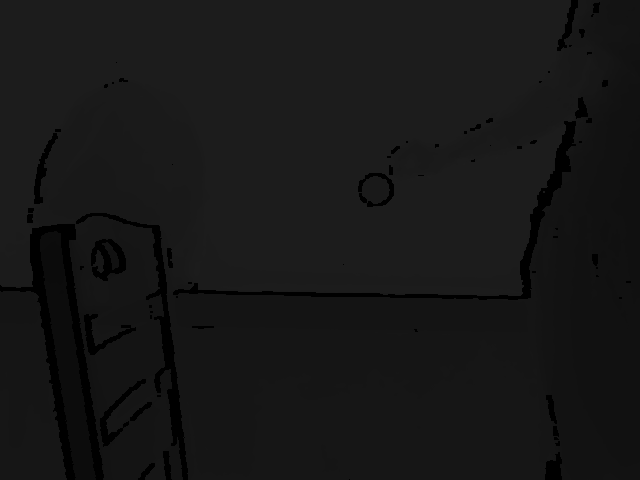

Supplement: Supplementary file 1 [file Data_Sheet_1.ZIP › The experimental data/corresponding depth image/tof640-20gm-22543413-0075-range_gray.png]

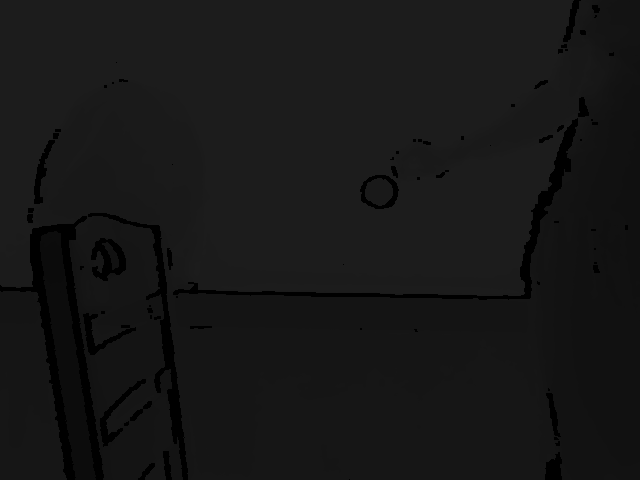

Supplement: Supplementary file 1 [file Data_Sheet_1.ZIP › The experimental data/corresponding depth image/tof640-20gm-22543413-0076-range_gray.png]

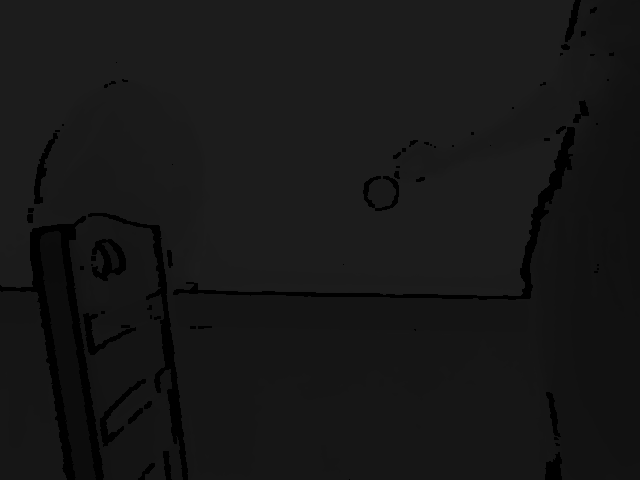

Supplement: Supplementary file 1 [file Data_Sheet_1.ZIP › The experimental data/corresponding depth image/tof640-20gm-22543413-0077-range_gray.png]

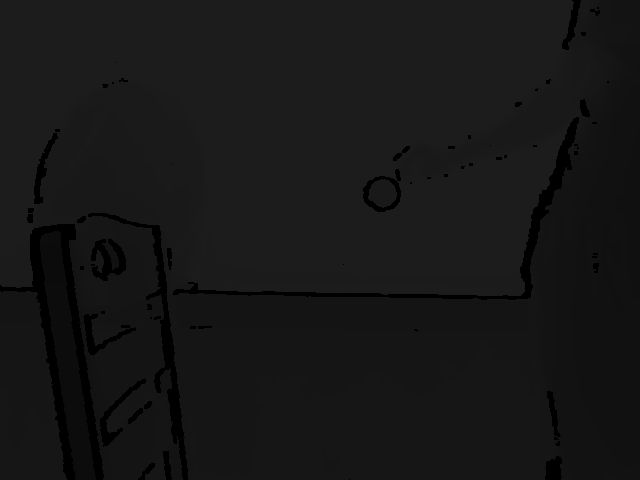

Supplement: Supplementary file 1 [file Data_Sheet_1.ZIP › The experimental data/corresponding depth image/tof640-20gm-22543413-0078-range_gray.png]

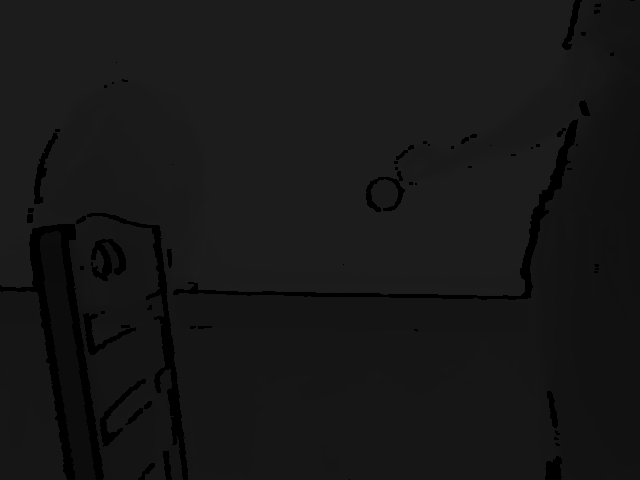

Supplement: Supplementary file 1 [file Data_Sheet_1.ZIP › The experimental data/corresponding depth image/tof640-20gm-22543413-0079-range_gray.png]

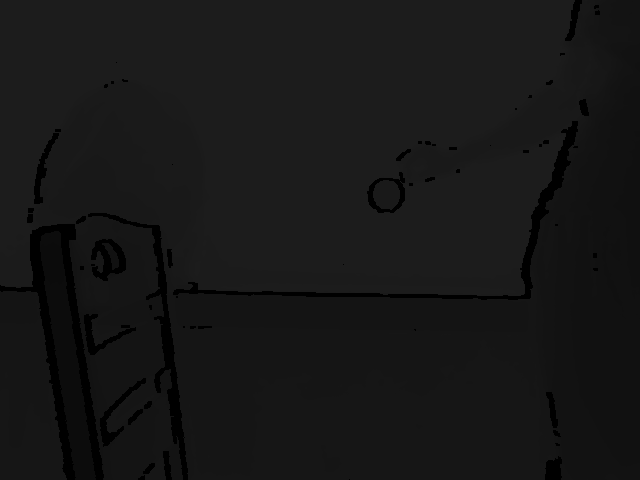

Supplement: Supplementary file 1 [file Data_Sheet_1.ZIP › The experimental data/corresponding depth image/tof640-20gm-22543413-0080-range_gray.png]

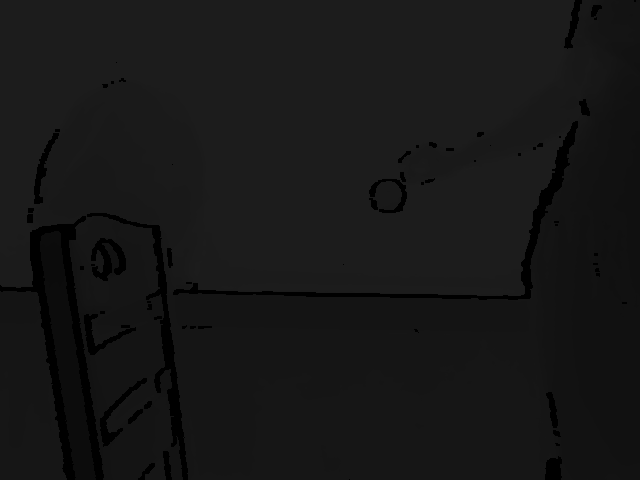

Supplement: Supplementary file 1 [file Data_Sheet_1.ZIP › The experimental data/corresponding depth image/tof640-20gm-22543413-0081-range_gray.png]

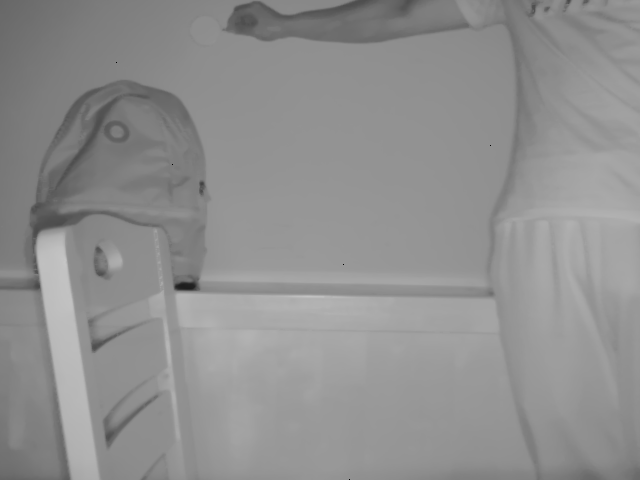

Supplement: Supplementary file 1 [file Data_Sheet_1.ZIP › The experimental data/grayscale image/tof640-20gm-22543413-0001-intensity.png]

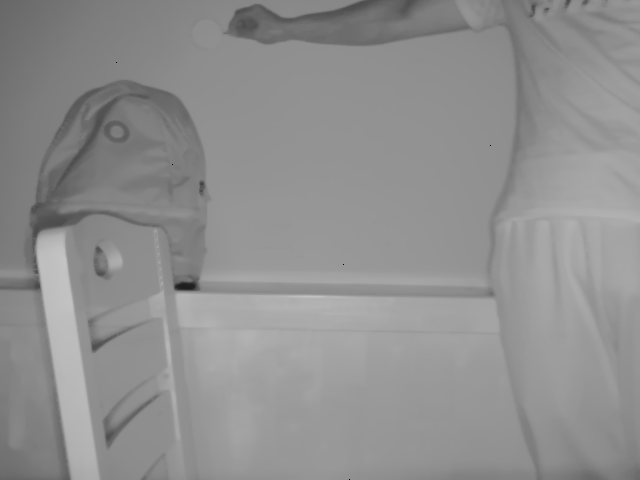

Supplement: Supplementary file 1 [file Data_Sheet_1.ZIP › The experimental data/grayscale image/tof640-20gm-22543413-0002-intensity.png]

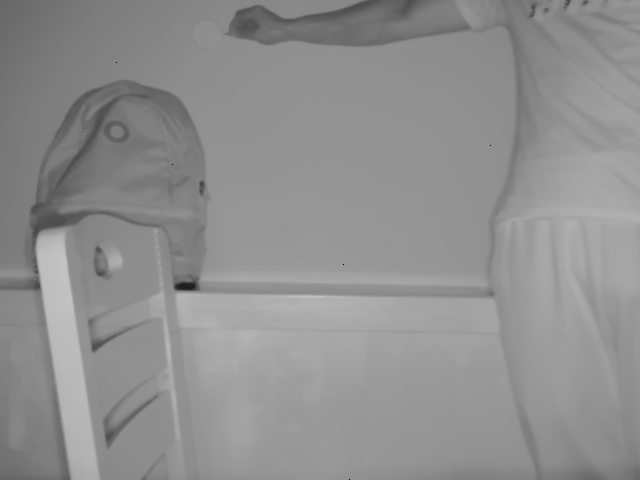

Supplement: Supplementary file 1 [file Data_Sheet_1.ZIP › The experimental data/grayscale image/tof640-20gm-22543413-0003-intensity.png]

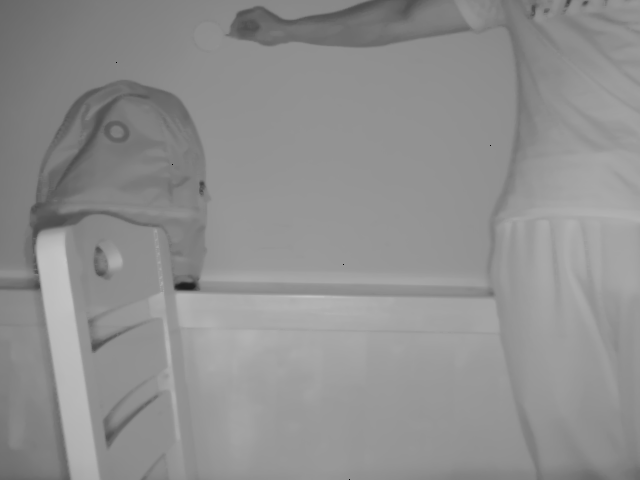

Supplement: Supplementary file 1 [file Data_Sheet_1.ZIP › The experimental data/grayscale image/tof640-20gm-22543413-0004-intensity.png]

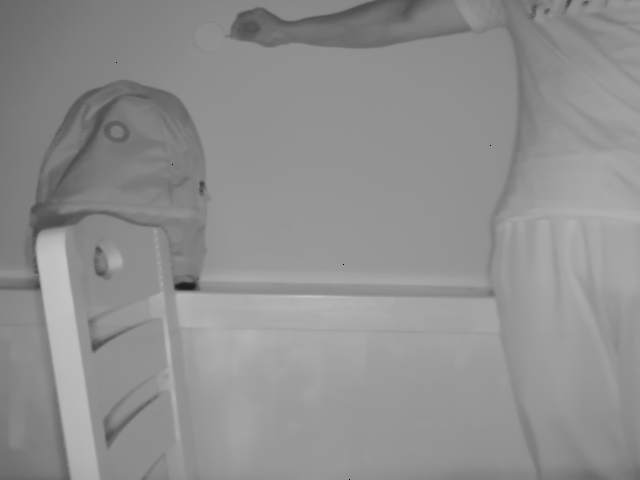

Supplement: Supplementary file 1 [file Data_Sheet_1.ZIP › The experimental data/grayscale image/tof640-20gm-22543413-0005-intensity.png]

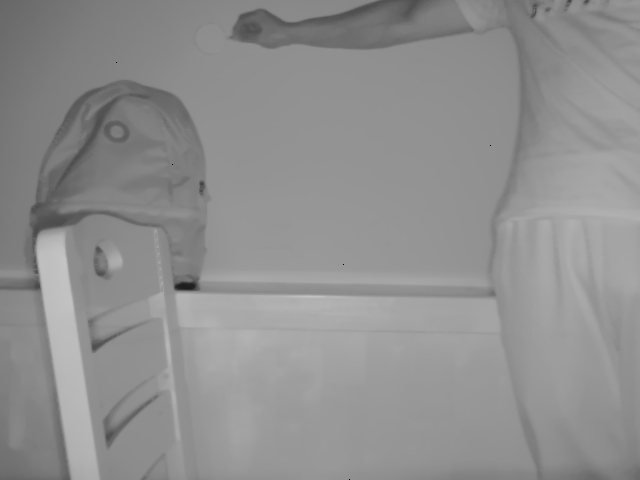

Supplement: Supplementary file 1 [file Data_Sheet_1.ZIP › The experimental data/grayscale image/tof640-20gm-22543413-0006-intensity.png]

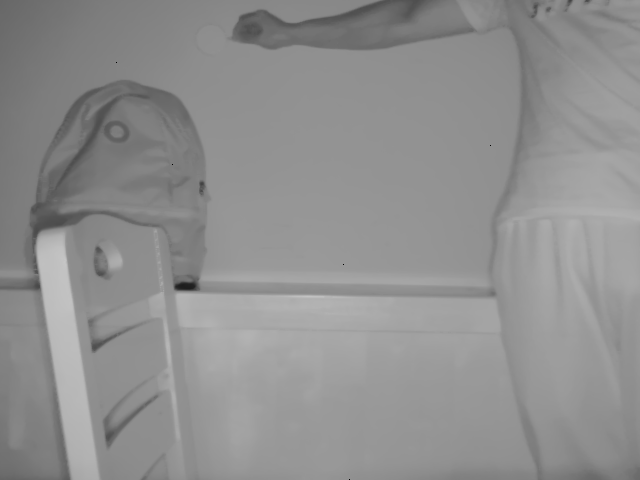

Supplement: Supplementary file 1 [file Data_Sheet_1.ZIP › The experimental data/grayscale image/tof640-20gm-22543413-0007-intensity.png]

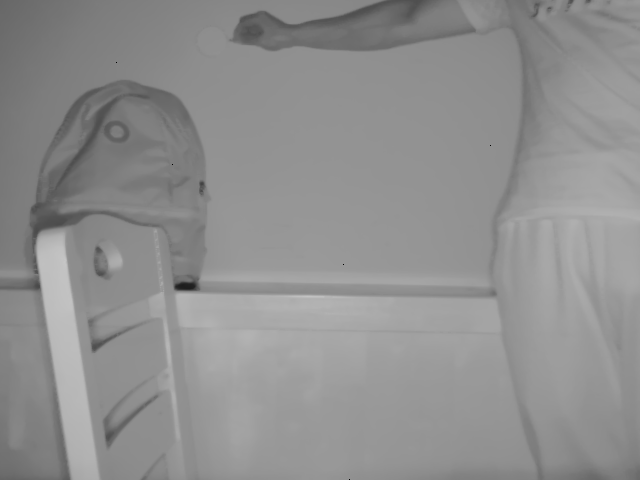

Supplement: Supplementary file 1 [file Data_Sheet_1.ZIP › The experimental data/grayscale image/tof640-20gm-22543413-0008-intensity.png]

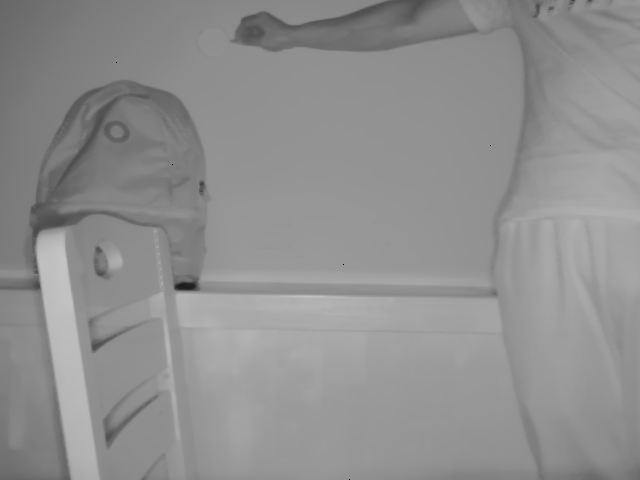

Supplement: Supplementary file 1 [file Data_Sheet_1.ZIP › The experimental data/grayscale image/tof640-20gm-22543413-0009-intensity.png]

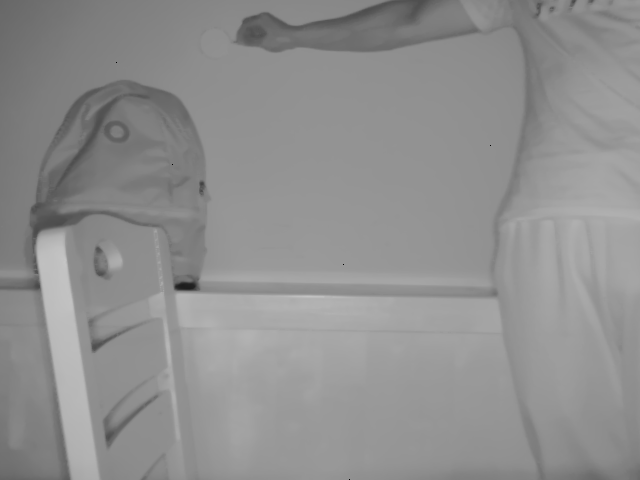

Supplement: Supplementary file 1 [file Data_Sheet_1.ZIP › The experimental data/grayscale image/tof640-20gm-22543413-0010-intensity.png]

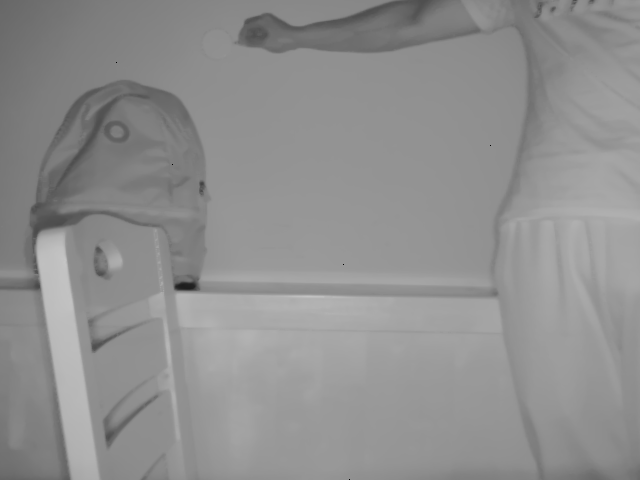

Supplement: Supplementary file 1 [file Data_Sheet_1.ZIP › The experimental data/grayscale image/tof640-20gm-22543413-0011-intensity.png]

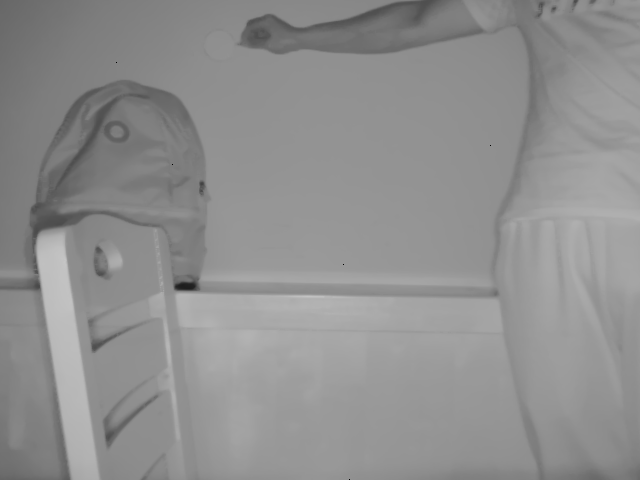

Supplement: Supplementary file 1 [file Data_Sheet_1.ZIP › The experimental data/grayscale image/tof640-20gm-22543413-0012-intensity.png]

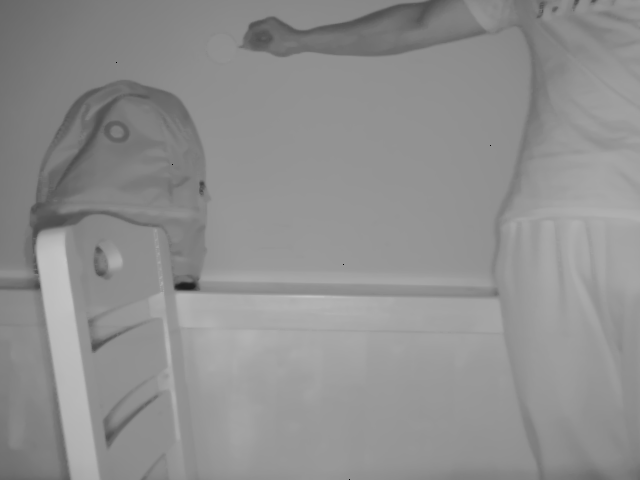

Supplement: Supplementary file 1 [file Data_Sheet_1.ZIP › The experimental data/grayscale image/tof640-20gm-22543413-0013-intensity.png]

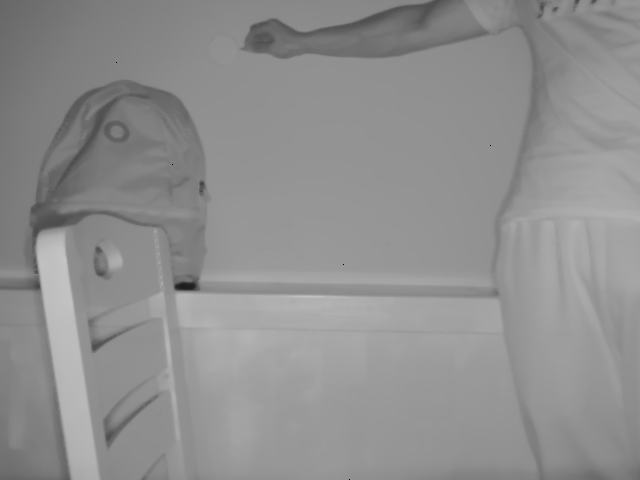

Supplement: Supplementary file 1 [file Data_Sheet_1.ZIP › The experimental data/grayscale image/tof640-20gm-22543413-0014-intensity.png]

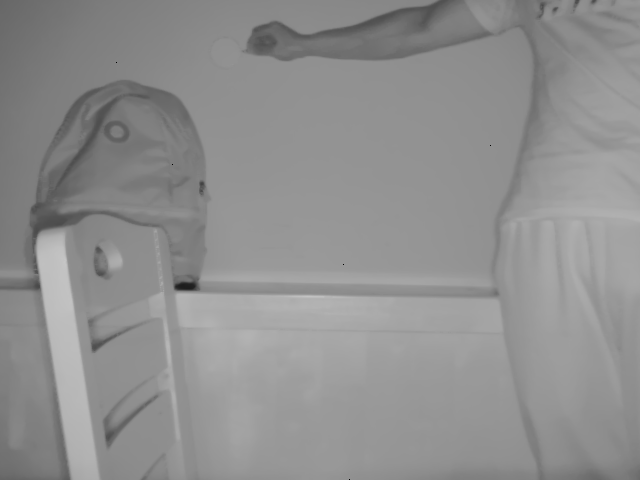

Supplement: Supplementary file 1 [file Data_Sheet_1.ZIP › The experimental data/grayscale image/tof640-20gm-22543413-0015-intensity.png]

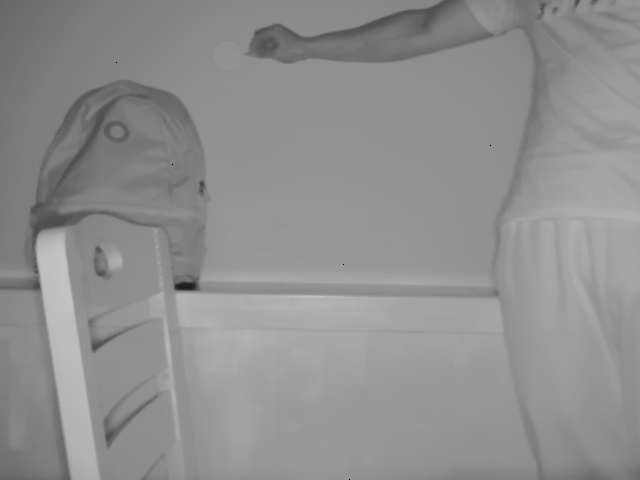

Supplement: Supplementary file 1 [file Data_Sheet_1.ZIP › The experimental data/grayscale image/tof640-20gm-22543413-0016-intensity.png]

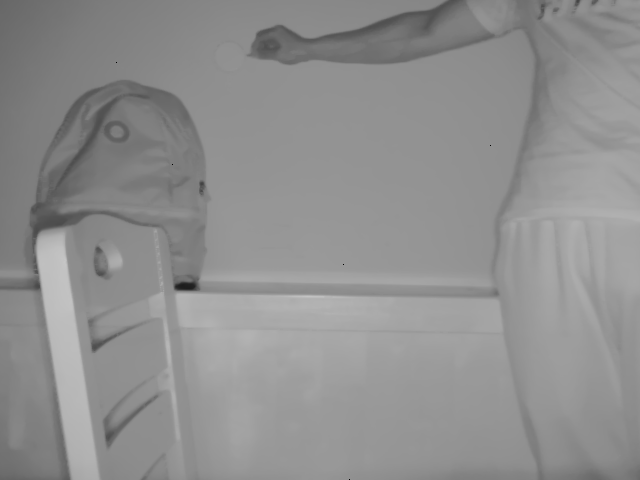

Supplement: Supplementary file 1 [file Data_Sheet_1.ZIP › The experimental data/grayscale image/tof640-20gm-22543413-0017-intensity.png]

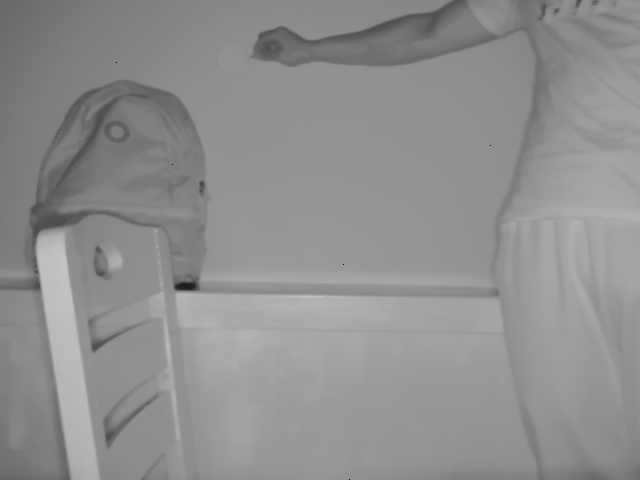

Supplement: Supplementary file 1 [file Data_Sheet_1.ZIP › The experimental data/grayscale image/tof640-20gm-22543413-0018-intensity.png]

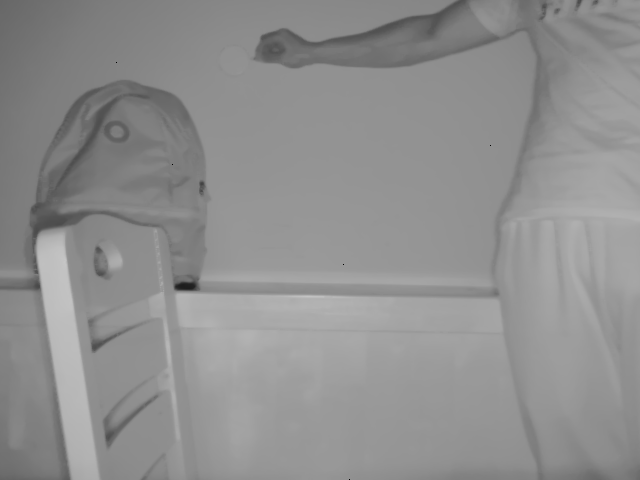

Supplement: Supplementary file 1 [file Data_Sheet_1.ZIP › The experimental data/grayscale image/tof640-20gm-22543413-0019-intensity.png]
